# CRISPR/Cas9-deaminase enables robust base editing in *Rhodobacter sphaeroides* 2.4.1

**DOI:** 10.1186/s12934-020-01345-w

**Published:** 2020-04-25

**Authors:** Yufeng Luo, Mei Ge, Bolun Wang, Changhong Sun, Junyi Wang, Yuyang Dong, Jianzhong Jeff Xi

**Affiliations:** 1 State Key Laboratory of Biomembrane and Membrane Biotechnology, Institute of Molecular Medicine, Peking University, Beijing, 100871 China; 2 Shanghai Laiyi Center for Biopharmaceutical R&D, 800 Dongchuan Road, Shanghai, 200240 China; 3 Department of Biomedical Engineering, State Key Laboratory of Natural and Biomimetic Drugs, College of Engineering, Peking University, Beijing, 100871 China; 4 Beijing Viewsolid Biotech Co. Ltd, Beijing, 100071 China; 5 Wallace H. Coulter Department of Biomedical Engineering, Georgia Institute of Technology and Emory University, 313 Ferst Drive NW, Atlanta, GA 30332 USA

**Keywords:** CRISPR/Cas9, Cytosine base editors, Adenine base editors, *Rhodobacter sphaeroides*, Coenzyme Q10

## Abstract

**Background:**

CRISPR/Cas9 systems have been repurposed as canonical genome editing tools in a variety of species, but no application for the model strain *Rhodobacter sphaeroides* 2.4.1 was unveiled.

**Results:**

Here we showed two kinds of programmable base editing systems, cytosine base editors (CBEs) and adenine base editors (ABEs), generated by fusing endonuclease Cas9 variant to cytosine deaminase PmCDA1 or heterodimer adenine deaminase TadA–TadA*, respectively. Using CBEs, we were able to obtain C-to-T mutation of single and double targets following the first induction step, with the efficiency of up to 97% and 43%; while the second induction step was needed in the case of triple target, with the screening rate of 47%. Using ABEs, we were only able to gain A-to-G mutation of single target after the second induction step, with the screening rate of 30%. Additionally, we performed a knockout analysis to identify the genes responsible for coenzyme Q10 biosynthesis and found that *ubiF*, *ubiA*, *ubiG*, and *ubiX* to be the most crucial ones.

**Conclusions:**

Together, CBEs and ABEs serve as alternative methods for genetic manipulation in *Rhodobacter sphaeroides* and will shed light on the fundamental research of other bacteria that are hard to be directly edited by Cas9-sgRNA.

## Background

Clustered regularly interspaced short palindromic repeats (CRISPRs) are adaptive immunity systems present in about 45% bacteria and 87% archaea [1, 2], which allow host organisms to prevent the invasion of foreign genetic elements such as viruses and plasmids [3, 4]. Type-II CRISPR associated protein 9 (Cas9) recognizes and cuts target DNA with the help of a chimeric single guide RNA (sgRNA), facilitating the double strand break (DSB) in a protospacer adjacent motif (PAM)-dependent manner [5–8].

Generally, DSBs are repaired either by error-prone non-homologous end joining (NHEJ) or precise homologous recombination (HR) [9]. As a simple and versatile device, Cas9-sgRNA complex has been widely utilized in eukaryotes [10]. However, relevant work is less reported in bacteria, mainly due to the lack of intrinsic NHEJ and the low efficiency of endogenous HR, which fails to repair the DSB in time and causes a quick death of the strains [11, 12].

*Rhodobacter sphaeroides* 2.4.1, originally discovered in 1989, is a gram negative and purple non-sulfur photosynthetic bacterium belonging to the α-3 subgroup of *Proteobacteria* [13]. As a talented producer of antioxidant coenzyme Q10 (CoQ10), it acts as the chassis microbe for industrial fermentation, demonstrating tremendous medical and commercial value. Early mutagenesis strategies usually relied on physical (UV, Ar, Co_60_, etc.) or chemical (LiCl, NTG, NaN_3_, etc.) substrates to breed it for a higher titer of CoQ10, which induced random mutations that could not be easily traced [14]. Current gene knockout (KO) techniques, including Tn5 transposon and double crossover, were relatively accurate but still time-consuming, labor-intensive, inefficient, and coverage or throughput-restricted, which hampered the large-scale functional gene study [15, 16].

Therefore, new approaches based on the CRISPR/Cas9 systems were urged to be developed. The genomic sequence of *Rhodobacter sphaeroides* 2.4.1 is comprised of two chromosomes (CI and CII) and five plasmids (A, B, C, D and E), which is GC-rich (68.8%) and suitable for searching a 5′-NGG-3′ PAM [17].

Cytosine deaminase creates uracil (U) through the deamination of cytosine (C), which is then read as thymine (T) in the process of DNA replication, ultimately leading to a C-to-T (C–T) substitution. APOBEC1 (BE3, derived from *Rat*) and PmCDA1 (CDA1, derived from *Sea lamprey*) are two natural and state-of-the-art enzymes that have been extensively used to obtain this goal [18, 19]. Furthermore, uracil DNA glycosylase inhibitor (UGI, derived from *Bacillus phage*) prevents the removal of U and promotes the occurrence of C-T. Similarly, adenine deaminase yields inosine (I) via the deamination of adenine (A), which is then treated like guanine (G) and eventually generating an A-to-G (A-G) substitution. The seventh version heterodimer ABE7.10 (TadA–TadA*), continuously evolved from *Escherichia coli* tRNA adenine deaminase (TadA), is the one and only enzyme that can elicit A-G in DNA until now [20, 21].

In this paper, we constructed cytosine base editors (CBEs) by ligating CDA1 and UGI to the carboxy terminus of dCas9 or nCas9D10A (named as dCBE or nCBE, respectively) and constructed adenine base editors (ABEs) by linking a codon-optimized TadA–TadA*(opt) to the amino terminus of dCas9 or nCas9D10A (named as dABE or nABE, respectively). We demonstrated that CBEs and ABEs were robust base editing systems for *Rhodobacter sphaeroides* 2.4.1 that allowed the efficient modification of multiplex genes in a stringent and chemically inducible manner. To our knowledge, this is the first instance of introducing C-T and A-G base conversion in the genus of *Rhodobacter sphaeroides*. Importantly, we also performed a proof-of-concept functional screening in this model strain and identified key genes in CoQ10 biosynthesis regulation.

## Materials and methods

### Strains, plasmids, and primers

The strains and plasmids used in this study are listed (Table 1). The primers used for molecular cloning (Additional file 1: Table S1) and Sanger sequencing (Additional file 1: Table S2) are also listed.

**Table 1:** Bacterial strains and plasmids used in this study

Strains/plasmids	Characteristics	Source
*Rhodobacter sphaeroides* 2.4.1	Wild type	ATCC 17023
*Rhodobacter sphaeroides* KD131	Wild type	This lab
*Escherichia coli* Trans1-T1	F^−^φ80(*lacZ*)ΔM15Δ*lac*X74*hsd*R(r_k_^−^, m_k_^+^)Δ*rec*A1398*end*A1*ton*A	Transgene CD501-01
pACYC-Cas9(dCas9/nCas9D10A)	Derive from pACYCDuet-1, Cm^R^, insert Cas9 or its variant in MCS1	This lab
pSCI_dCas9-CDA1-UL	Expression plasmid, Cm^R^, contain dCas9, CDA1, UGI, and LVA tag	Gift from Prof. Satomi Banno et al.
pgRNA-bacteria	Derive from pUC19, Amp^R^, contain sgRNA scaffold	Addgene 44251
pMV-Tada-TadA*(opt)	Gene synthetic plasmid, Amp^R^, contain codon-optimized TadA–TadA*(opt)	Wuxi Qinglan Biotechnology Inc.
pIND4	Expression plasmid, pMB1 ori, LacI, Kana^R^	Gift from Prof. Judith P.Artimage
pK18mobSacB	Suicide plasmid, pMB1 ori, SacB, Kana^R^	This lab
pIND4-SacB-MCS	Derive from pIND4, insert multiple cloning site(MCS)and SacB	This study
pIND4-Cas9-sgRNA	Derive from pIND4-SacB-MCS, insert Cas9 and sgRNA	This study
pIND4-Cas9-sgRNA-up-dw	Derive from pIND4-SacB-MCS, insert Cas9, sgRNA, and homologous arms	This study
pIND4-dCas9(nCas9D10A)-CDA1-UL-sgRNA	Derive from pIND4-SacB-MCS, abbreviated as d/nCBE-sgRNA	This study
pIND4-TadA–TadA*(opt)-dCas9(nCas9D10A)-sgRNA	Derive from pIND4-SacB-MCS, abbreviated as d/nABE-sgRNA	This study

### Target design

For CBEs, if the coding strand of a gene contained a 5′–3′ direction sequence “C#NGG” or “CCN#G”, with a gap# = 13–19 bp, we defined it as mutable. When this sequence matched one of the following conditions, we further defined it as knockout ready: (a) “CGA#NGG” or “CAG#NGG” or “CAA#NGG”, of which “CGA” or “CAG” or “CAA” was in frame of the start codon, with a gap# = 11–17 bp; (b) “CCN#TGG”, of which “TGG” was in frame of the start codon, with a gap# = 11–18 bp; (c) “CCN#NTG”, of which “NTG” was the start codon, with a gap# = 11–17 bp.

For ABEs, if the coding strand of a gene contained a 5′–3′ direction sequence “A#NGG” or “CCN#T”, with a gap# = 13–15 bp, we defined it as mutable. When this sequence was “CCN#NTG”, of which “NTG” was the start codon, with a gap# = 12–14 bp, we further defined it as knockout ready.

### Plasmid construction

As there were no multiple cloning sites (MCS) in pIND4, we first digested it with NcoI and HindIII, then annealed two pairs of primers containing some custom restriction endonucleases to acquire pIND4-MCS. Next, we used MscI to cut pIND4-MCS and inserted SacB amplified from pK18mobSacB to get the intermediate pIND4-SacB-MCS.

We took pgRNA-bacteria as the template for the amplification of a sgRNA scaffold. To edit a single target, we amplified one sgRNA; to edit a double target, we amplified two sgRNAs and then joined them with gene splicing by overlap extension PCR (SOE PCR); to edit a multiplex target, we first amplified each sgRNA individually, then linked every two sgRNAs with SOE PCR, and finally connected them with Gibson assembly kit. For the purposes of unbiased transcription, each sgRNA was driven by its own promotor and terminator.

To construct the Cas9-sgRNA system, we digested pIND4-SacB-MCS with AgeI and SpeI, then linked Cas9 to obtain pIND4-Cas9. We further cleaved it with SalI and BglII, and ligated a sgRNA cassette to acquire pIND4-Cas9-sgRNA. Next, it was cut with NotI, and the homologous upstream and downstream arms flanking the target region were amplified and inserted to form pIND4-Cas9-sgRNA-up-dw.

To construct the CBEs, we digested pIND4-SacB-MCS with AgeI and SpeI. We further linked the dCas9 or nCas9D10A to the CDA1 and UGI, which were amplified from pSCI_dCas9-CDA1-UL or pACYC-nCas9D10A and sequentially fused by Gibson assembly kit, generating pIND4-dCas9-CDA1-UL and pIND4-nCas9D10A-CDA1-UL (abbreviated here on as dCBE and nCBE, respectively). We cleaved dCBE or nCBE with SpeI and SalI, then ligated them with a sgRNA cassette to form the all-in-one C-T base editing plasmids, namely dCBE-sgRNA or nCBE-sgRNA.

To construct the ABEs, we digested pIND4-SacB-MCS with NcoI and AgeI. We further linked the TadA–TadA*(opt) amplified from pMV-TadA–TadA*(opt) to obtain pIND4-TadA–TadA*(opt). Next, it was linearized with AgeI and Spe, and we inserted dCas9 or nCas9D10A to generate pIND4-TadA–TadA*(opt)-dCas9 and pIND4-TadA–TadA*(opt)-nCas9D10A (abbreviated here on as dABE and nABE, respectively). Likewise, we cleaved dABE or nABE by BglII, then ligated them with a sgRNA cassette to form the all-in-one A-G base editing plasmids, namely dABE-sgRNA or nABE-sgRNA.

### Strain culture

For *Escherichia coli* Trans1-T1, Luria–Bertani (LB) liquid medium (10 g/L Tryptone, 5 g/L Yeast extract, 10 g/L NaCl, PH adjusted to 7.0) was used and incubated at 37 °C. For *Rhodobacter sphaeroides* 2.4.1 and KD131, PYG liquid medium (10 g/L Tryptone, 5 g/L Yeast extract, 1 g/L Glucose, PH adjusted to 6.8–7.0) was used and incubated at 30 °C. 1.5–2% agar was added when LB and PYG solid plates were used. The CoQ10 fermentation liquid medium (65 g/L Glucose, 19 g/L MgSO_4_·7H_2_O, 5 g/L Yeast extract, 12 g/L Glutamate, 5 g/L KH_2_PO_4_, 5 g/L Na_2_HPO_4_, 10 g/L (NH_4_)_2_SO_4_, 1.7 g/L FeSO_4_·7H_2_O, 8 ml/L simplified trace element) was used and incubated at 34 °C.

The working concentrations of chemical substrates used in this study were as follows: Isopropyl-β-d-thiogalactopyranoside (IPTG) (0.5 mM), Kanamycin (25 μg/mL), Glycerol (10% V/V), Sucrose (10% W/V).

### Electrocompetent cell preparation and transformation

Electrocompetent cells are prepared with the following protocol: (1) inoculate a single colony in a 10 mL tube for overnight growth; (2) transfer 1% V/V strains into a 500 mL shake flask with 100 mL PYG liquid medium; (3) when optical density at 600 nm (OD_600_) of the bacterial culture reaches 0.4-0.6, place it on ice and chill for 30 min; (4) spin in centrifuge at 5000 rpm for 10 min, discard the supernatant, and add 40 ml ddH_2_0 to wash the cell pellet; (5) spin in centrifuge at 6000 rpm for 10 min, discard the supernatant, and add 40 ml 10% V/V glycerol to wash the pellet; (6) repeat step (5) with 20 ml then 10 ml 10% V/V glycerol, successively; (7) spin in centrifuge at 6000 rpm for 10 min, discard the supernatant, and add 5 ml 10% V/V glycerol to resuspend the pellet; (8) distribute the final dilutions (100 μl each) into the sterile 1.5 ml tubes, freeze it immediately with liquid nitrogen and store at − 80 °C.

About 1 μg of plasmid was pipetted into 100 μl thawed electrocompetent cells, then we transferred it to a pre-cooled 1 mm cuvette (Bio-Rad, 1.8 kV, 100 Ω, 25 μF). After audible pulse, we added 900 μL pre-warmed PYG liquid medium (containing 0.5 mM IPTG if necessary) and recovered it in tube at 30 °C for 3–4 h (hrs). Finally, we spread this culture onto PYG agar plate (containing 25 μg/ml Kanamycin and 0.5 mM IPTG if necessary) and incubated it at 30 °C for 5–7 days until the colonies were visible. The transformation efficiency was calculated as the number of colony forming units per 1 μg of plasmid (cfu/μg).

### Western blot

First, we electroporated the control sgRNA plasmids of CBEs and ABEs (dCBE-ctrl, nCBE-ctrl, dABE-ctrl, and nABE-ctrl) into the strain *Rhodobacter sphaeroides* 2.4.1. Next, we inoculated the transformants in 10 mL tubes and added 0 mM or 0.5 mM IPTG to the bacterial culture when OD_600_ reached 0.6-0.8. Following a 12 h induction step at 16 °C, the cell pellets were harvested and boiled for 10 min.

Then, we got the total protein samples and ran the SDS-PAGE for 1.5 h (5% stacking gel at 80 V for 30 min and 8% separation gel at 120 V for 1 h, successively). After that, we excised and shifted the protein bands to the PVDF membrane at 250 mA for 2 h. We used “Anti-CRISPR-Cas9 [7A9-3A3] (ab202580)” binding to the amino terminus of Cas9 as the primary antibody (advisedly diluted at 1:2000) and “Goat anti mouse IgG H&L (ab205719)” as the secondary antibody (advisedly diluted at 1:3000).

We predicted that the size of the CBEs fusion protein (d/nCas9 + CDA1 + UGI) to be 207KD and that of the ABEs fusion protein (TadA–TadA*(opt) + d/nCas9) to be 201KD. To parallel the aforementioned base editing system plasmids, the Cas9-sgRNA system plasmid pIND4-Cas9-ctrl was also tested and the size of Cas9 was 158KD.

### Editing efficiency and activity window

In order to evaluate the editing efficiency, we selected ten single colonies from each plate. We resuspended each colony in 10 μL ddH_2_0, took 1 μL of this solution as the PCR template, and then designed primer set (about 1 Kb upstream and downstream flanking the spacer) for amplification (30 cycles). After agarose gel electrophoresis, we excised the approximate 2 Kb band of the above tested colonies, sent it for Sanger sequencing. The band of the wild type (WT) strain was used as the negative control. At the same time, we spread 2 μL of the corresponding bacterial liquid onto PYG antibiotic-free plates for phenotype observation and culture preservation.

We divided the tested colonies first into two categories (WT and mutant). For CBEs, when targeting a spacer that may contain several Cs, if no C was mutated to T, we classified it as a WT; if one C was partially or totally mutated to T, we classified it as a mutant. Similarly, for ABEs, when targeting a spacer that may contain several As, if no A was mutated to G, we classified it as a WT; if one A was partially or totally mutated to G, we classified it as a mutant.

We divided these mutants further into three genotypes based on the peak area of the DNA nucleotides: C versus T for CBEs; A versus G for ABEs. For mutants generated using CBEs, if one C was totally mutated to T, we considered it as a good (T) mutant, which was very easily to be isolated for obtaining pure colonies with a screening rate of nearly 100% (data not shown); if the areas under the C peaks were equal to or smaller than that of the T peaks, we considered it as a moderate (T≥C) mutant, which was not difficult to be isolated, with the screening rate of more than 50% (data not shown); if the areas under the C peaks were all greater than that of the T peaks, we considered it as a bad (T<C) mutant, with a screening rate of less than 50% (data not shown). Similarly, for mutants generated using ABEs, if one A was totally mutated to G, we considered it as a good (G) mutant; if the areas under the A peaks were equal to or smaller than that of the G peaks, we considered it as a moderate (G≥A) mutant; if the areas under the A peaks were all greater than that of the G peaks, we considered it as a bad (G<A) mutant.

For both CBEs and ABEs, we calculated the final editing efficiency as (the number of good and moderate mutants/the number of tested colonies), which was shown as (average ± standard deviation) in the figures.

For a certain target, Cs or As at different positions varied in mutation frequency. We nominated the position where the peak of C or A was equal to or smaller than that of T or G as a hot position and counted the mutation frequency of each hot position individually. It should be noted that we defined the activity window as the spectrum of all hot positions.

We measured the percentage of three mutation subtypes for non-specific mutation analysis: C-T, C-G, and C-A for CBEs; A-G, A-C, and A-T for ABEs. For every position with a C or an A, we measured the percentage of these mutation subtypes independently.

### Plasmid curing and iterative editing

We streaked the mutant (contained plasmid) on PYG agar plate (10% W/V sucrose) to get the mutant without the plasmid. Three pairs of primers binding to the *Kana*, *Cas9*, and *rpoZ* genes were designed to validate the removal of plasmid. Compared with the bright bands of positive control (WT with plasmid), the streaked ∆appA1, ∆crtB1, and WT displayed no band after PCR with primers for *Kana* and *Cas9* (25 cycles), which were located on the plasmid. Meanwhile, all tested colonies showed bright bands after PCR with the primers for *rpoZ* (25 cycles), which was located on the chromosome. Moreover, the streaked ∆appA1 and ∆crtB1 strains did not grow on the PYG agar plate (25 μg/mL kanamycin), further proving that the plasmid was removed completely in the strains.

We prepared the above streaked and plasmid cured ∆appA1 and ∆crtB1 strains as fresh competent cells, and then electroporated a new plasmid carrying another sgRNA into them. Following the previously described procedures, we successfully obtained the latter colonies harboring a new target mutation based on the genetic background of former single target mutant, with an efficacy equivalent to that of the WT, indicating that iterative editing was feasible.

### CoQ10 fermentation and quantification

We inoculated the candidate CoQ10 production strain in tube, with 190 rpm and 32 °C for 24 h of growth. Next, we transferred it into a 25 ml shake flask, with 190 rpm and 34 °C for 5 days of fermentation. Afterwards, we took 1 ml of the bacterial culture (1:3 dilution) to extract CoQ10. Then, we passed the resulting sample through HPLC, using a standard CoQ10 setting as the positive control. The CoQ10 concentration of each sample was calculated as [(the peak area of sample/the peak area of standard) × the concentration of standard × 3] (mg/L). The CoQ10 content of each sample was normalized to [(the CoQ10 concentration of each sample/the packed cell volume) (mg/L PCV).

## Results

### Construction and evaluation of the IPTG-inducible CBEs

We chose pIND4, a shuttle vector propagated in *Escherichia coli* and *Rhodobacter sphaeroides*, as the backbone [22]. First, we employed the wild type *Streptococcus pyogenes* Cas9 (driven by the inducible promotor pLac) along with sgRNA (driven by the constitutive promotor pJ23119) in this strain (Additional file 1: Figure S1A, B). For a proof-of-concept experiment, we selected the sgRNA appA2 (which targeted *appA* (*RSP_1565*) gene and displayed high efficiency with CBEs) for simply testing Cas9-sgRNA system, while the sgRNA ctrl (which targeted *sfGFP* gene) setting as the negative control (Additional file 1: Figure S1C). Although the annotated Ku (*RSP_0524*) and LigD (*RSP_2679*) genes existed in its genome, fewer than ten colonies survived and no insertion or deletion (indel) mutation was observed, suggesting that the NHEJ machinery was either inactive or dysfunctional (Additional file 1: Figure S1D, E). Next, we extra supplied the donor of linear single strand DNA (ssDNA) or circular double strand DNA (dsDNA) (Additional file 1: Figure S1F, G). However, ~ 90nt length oligo (5′ and 3′ terminal modified by phosphonothioate bond) failed to help acquire the point mutant, implying that the simultaneous heterologous expression of a recombineering system (such as RecE/T or λ-Red) may be required; and only with the plasmid (containing the homologous 1 Kb upstream and downstream arms) were we able to gain the gene middle fragment deletion mutants, indicating the weak capacity of HR (Additional file 1: Figure S1H-I). In short, Cas9-sgRNA mediated genome editing did not practically improve the repairing efficiency, resulted in severe lethality, and was essentially a counter-selection module by killing the unmutated strains [23].

**Fig. 1:**
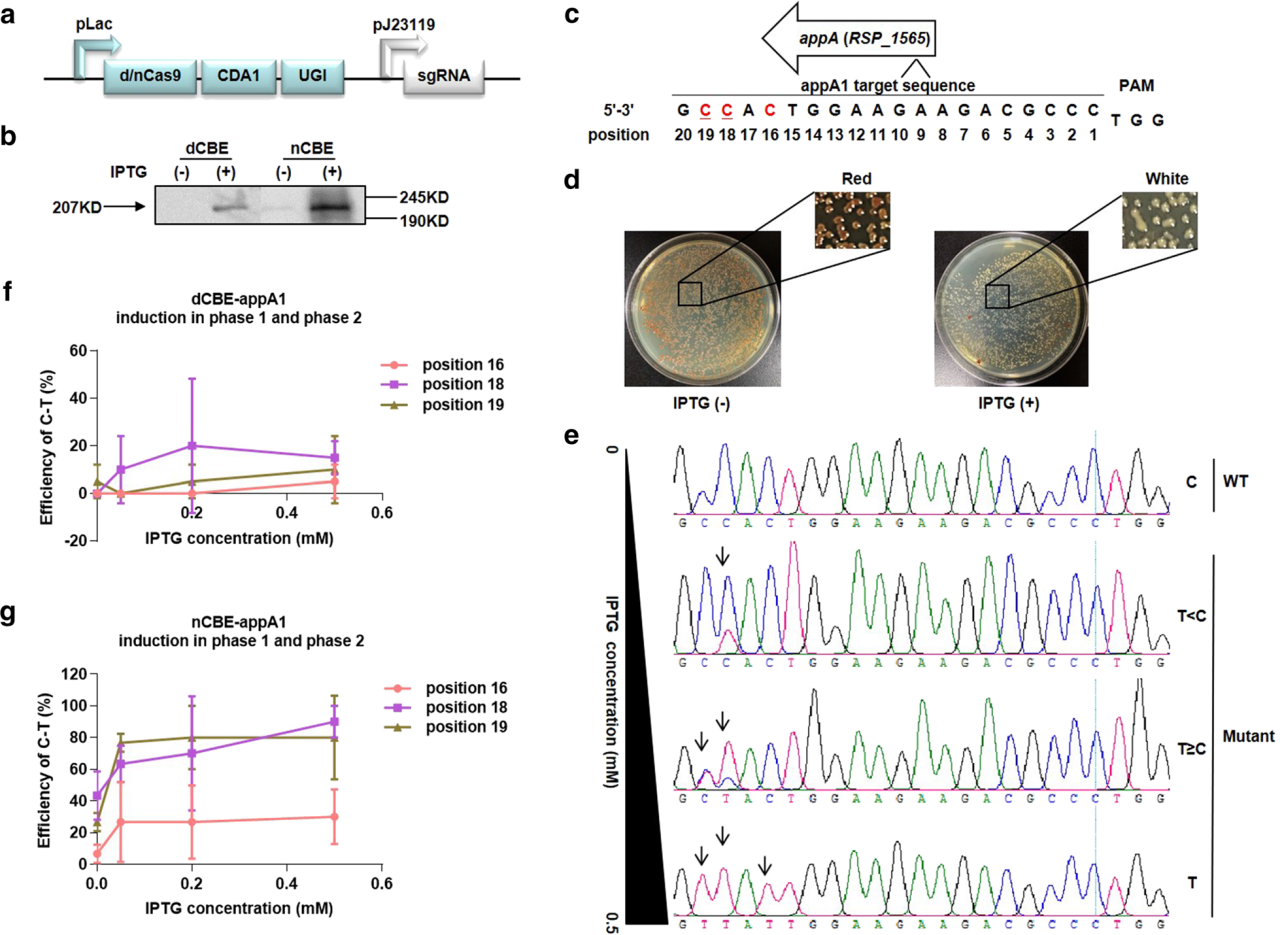
The IPTG-inducible CBEs enabled efficient C-T mutation. **a** The diagrammatic sketch of main components in CBEs. **b** The western blot of dCBE and nCBE, each with 0 mM or 0.5 mM IPTG induction, respectively. **c** The sgRNA appA1 target sequence (20 bp spacer) was located in the non-template strand of gene *appA*, 5′ upstream from the PAM “TGG”. The position of each nucleotide (1–20) was defined as follows: the 3′ end closest to the PAM nominated as 1, while the 5′ end farthest nominated as 20. The red colored Cs meant the editable Cs in this target, and the underlined Cs were the key nucleotides that could be mutated to produce stop codon. **d** Two PYG agar plates (left with 0 mM IPTG, right with 0.5 mM IPTG) were displayed, where the majorities of colonies were red and white, respectively. **e** Four typical genotypes (C, T<C, T≥C, and T) were listed with the increase of IPTG. Black arrows indicated the position where the mutation peaks of T appeared. **f**,** g** After the plasmid dCBE-appA1 or nCBE-appA1 was electroporated, the efficiencies of C-T at position 16, 18, and 19 in appA1 target sequence were individually calculated for dCBE-appA1 and nCBE-appA1, with the IPTG induction of constant concentration 0.5 mM in phase 1 and various concentration ranging from 0 to 0.5 mM in phase 2

So, we sought to bypass the DSB with the combination of CRISPR/Cas9 and deaminase. Cas9 variant (dCas9 or nCas9D10A) coupling with CDA1 and UGI as a whole was driven by the inducible promotor pLac, whereas the sgRNA itself was driven by the constitutive promotor pJ23119 (Fig. 1a). We first confirmed the translation of a fusion protein dCas9-CDA1-UGI or nCas9D10A-CDA1-UGI (referred to as dCBE or nCBE, respectively): western blot analysis reflected that the hybridization band could be just seen for dCBE and much thicker for nCBE in the presence of IPTG (Fig. 1b).

Next, we tested whether dCBE or nCBE was effective in vivo under the guide of a target sgRNA. Similar to Cas9-sgRNA system, we selected the gene *appA* (*RSP_1565*) as the reportor. In contrast to the red pigment of wild type (WT), ΔappA turned white [24]. Then, we designed a sgRNA (termed appA1) containing the 20 bp spacer 5′-GCCACTGGAAGAAGACGCCC-3′ to knock out *appA* (Fig. 1c). The spacer had a “CCA” anticodon (underlined for clarity) in the non-template strand, corresponding to a “TGG” codon in the template strand; when one or both Cs of “CCA” were mutated to Ts (the Gs on the opposite were singly or doubly converted to As), a premature stop codon (“TGA” or “TAG” or “TAA”) would be formed and subsequently produce a loss-of-function of *appA*.

Instead of tedious and laborious bi-parental conjugation, we optimized a facile and valid electric pulse-delivery protocol on the basis of previous described method, to transform strains with plasmids, especially those of large sizes (> 10 Kb) [25]. Normally, the transformation consists of two growth stages: the recovery phase 1 in a tube and the incubation phase 2 on a plate (Additional file 1: Figure S2A). Once we electroporated the plasmid dCBE-appA1 or nCBE-appA1 into the strain *Rhodobacter sphaeroides* 2.4.1, we added various concentration of IPTG to the PYG agar plate (25 μg/ml Kanamycin). White colonies appeared and occupied the majorities with the increase of IPTG, indicating that the appA1 target sequence may have been rewritten as expected (Fig. 1d). To confirm that DNA mutation rather than RNA interference was responsible for the pigment phenotype, we picked ten colonies from each plate, amplified the target region, and sent the PCR product for Sanger sequencing.

We initially profiled Cs at position 1, 2, 3, 5, 16, 18, and 19 in appA1 target to estimate the efficiency of C-T. According to the sequencing data (Additional file 1: Table S3), we found that Cs at positions 16, 18, and 19 were more likely to be mutated to Ts while other Cs remained unchanged, in accordance with previous literature stating the activity window of CDA1 was around “16-20” [20]. For example, four typical genotypes (C, T<C, T≥C, and T) were listed as a representative of each group (0 mM, 0.05 mM, 0.2 mM, and 0.5 mM), suggesting a dose-dependent mutation pattern with the increase of IPTG (Fig. 1e).

It was clear that a concentration of 0.5 mM IPTG performed the best in phase 2. For dCBE-appA1, with the concentration of IPTG increasing from 0 mM to 0.5 mM, although the efficiency of C-T at position 16 was still 0%, the efficiencies of C-T at position 18 and 19 were elevated from 0% to 10% and 0% to 5%, respectively (Additional file 1: Figure S2B). Similarly, for nCBE-appA1, with the concentration of IPTG increasing from 0 mM to 0.5 mM, the efficiencies of C-T at position 16, 18, and 19 were elevated from 0% to 30%, 3.3% to 90%, and 3.3% to 90%, respectively (Additional file 1: Figure S2C). Based on this observation, we added 0.5 mM IPTG in phase 1 as well. After that, the efficiencies of C-T at these three positions were either boosted to a higher degree or maintained at the level of saturation (Fig. 1f, g).

Despite the larger proportions of C-T mutation subtype, excessive induction by IPTG tended to cause non-specific C-G and C-A mutation subtypes for nCBE-appA1 but not for dCBE-appA1 (Additional file 1: Figure S2D–F), whose mechanism still remained elusive.

Here, we described that CBEs (dCBE and nCBE) were stringently IPTG-inducible with low basal leakage. If not stated, 0.5 mM IPTG was added both in phase 1 and phase 2.

### CBEs mediated single and double target editing

In view of the fact that nCBE-appA1 possessed not only a higher editing efficiency but also a broader activity window than dCBE-appA1, albeit at the sacrifice of byproduct C-G and C-A mutation subtypes. To prove if this was the common feature, we next systematically investigated dCBE and nCBE series with more single and double targets.

For single target editing, we tested the other sgRNA (termed appA2) of *appA*, which contained the 20 bp spacer 5′-CCATCCCGCAAAGCGGCGCT-3′. Like appA1, appA2 also had a “CCA” anticodon (underlined for clarity) and could cause loss-of-function with the introduction of a premature stop codon. We also assessed another gene *ppsR* (*RSP_0282*), which was correlated with *appA* and played an important role in the modulation of light and redox signaling [26]. In contrast to the red pigment of WT, ΔppsR turned dark red. Two sgRNAs (termed ppsR1 and ppsR2) were deliberately designed to knock out *ppsR*, containing the 20 bp spacer 5′-CGACTCACCACCGACTTCGC-3′ and 5′-CCAGGTGGCCGAGATCTCTG-3, respectively. When the C of “CGA” or “CAG” codon (underlined for clarity) was mutated to T, a premature stop codon (“TGA” for ppsR1 or “TAG” for ppsR2) was generated and *ppsR* was inactivated. For double target editing, we took appA1-appA2 (two sgRNAs located in one gene) and appA1-ppsR2 (two sgRNAs located in two genes) as an illustration (Additional file 1: Figure S3A). In this situation, only when both targets harbored the C-T did we consider it as a successful mutation event.

**Fig. 2:**
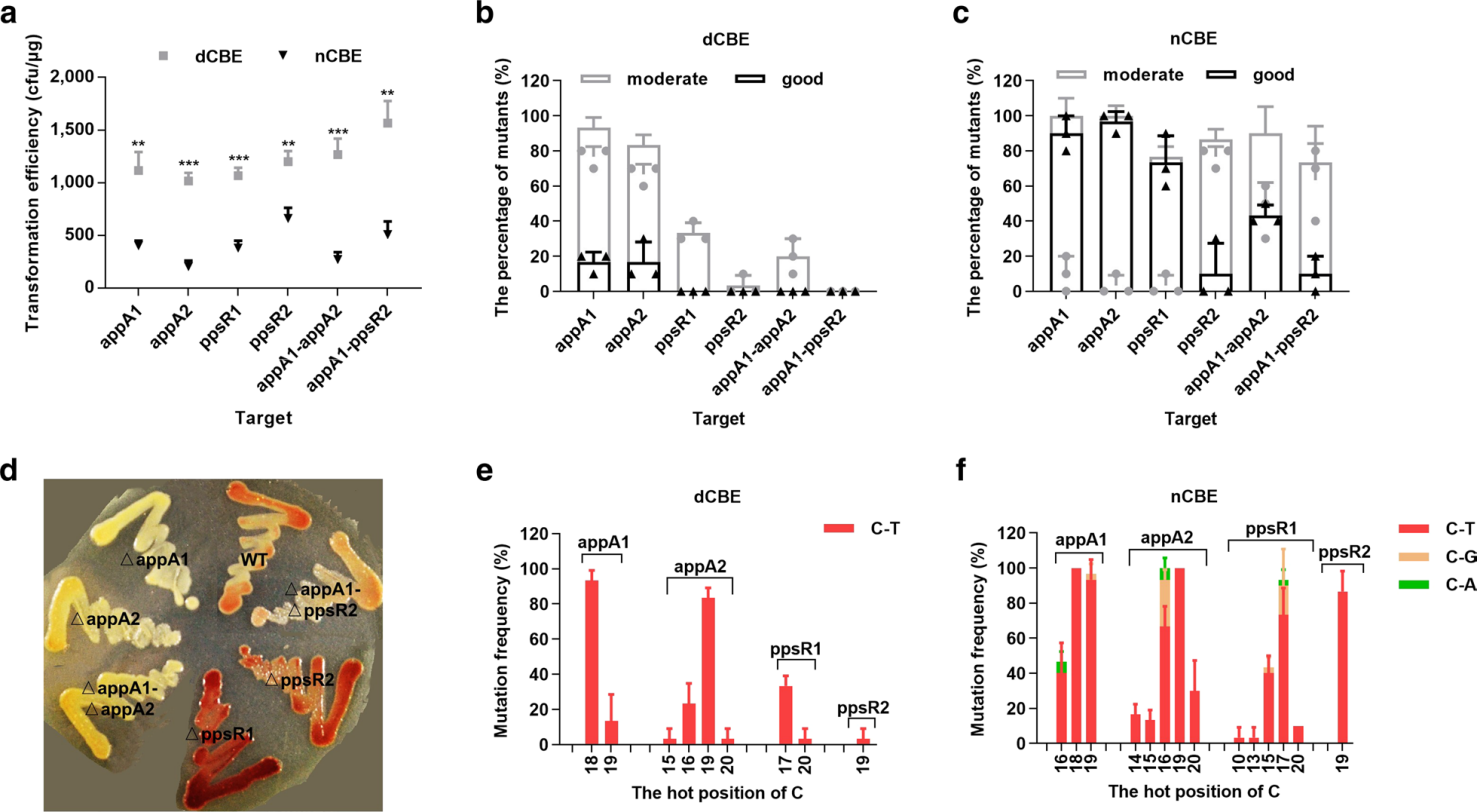
dCBE versus nCBE series in the aspects of transformation, editing efficiency and activity window. **a** The transformation efficiencies of dCBE and nCBE series targets. The significant analysis of t-test was shown as (*p < 0.05; **p < 0.01; ***p < 0.001). **b**, **c** The percentages of good (T) and moderate (T≥C) mutants for dCBE or nCBE series targets, respectively. **d** The pigment phenotype of WT and KO mutants for each target. **e**, **f** The hot position of C and its mutation frequency were shown for dCBE or nCBE series single target, respectively

We then constructed and electroporated dCBE or nCBE series plasmids with above sgRNAs into the strain *Rhodobacter sphaeroides* 2.4.1. When compared to the control sgRNA plates, each target sgRNA plate of dCBE or nCBE series showed modest or significant changes in pigment (Additional file 1: Figure S3B). Moreover, we also saw fewer colonies in nCBE series target sgRNA plates than those of dCBE series, a phenomenon likely attributed to the cleavage of the non-template strand (Fig. 2a).

According to the sequencing data (Additional file 1: Table S4), when editing a single target by dCBE series, we acquired good (T) mutants with an efficiency of 16.7% for appA1 and 16.7% for appA2 (Fig. 2b), while the percentages of KO mutant ΔappA1 and ΔappA2 were 16.7% and 13.3%, respectively. When editing a single or double target by nCBE series, we acquired good (T) mutants with an efficiency of 90% for appA1, 96.7% for appA2, 73.3% for ppsR1, 10% for ppsR2, 43.3% for appA1-appA2, and 10% for appA1-ppsR2 (Fig. 2c), while the percentages of KO mutant ΔappA1, ΔappA2, ΔppsR1, ΔppsR2, ΔappA1-ΔappA2, and ΔappA1-ΔppsR2 were 90%, 93.3%, 10%, 10%, 33.3%, and 10%, respectively. Moreover, we observed the subsequent change in pigment phenotype of KO mutants ΔappA1 (white), ΔappA2 (white), ΔppsR1 (dark red), ΔppsR2 (dark red), ΔappA1-ΔappA2 (white), and ΔappA1-ΔppsR2 (light red) by visual inspection (Fig. 2d).

The Cs at different positions in the same target showed diverse potential to be mutated. For the isolation of a pure C-T mutant, we defined positions where the peak areas of C were equal to or smaller than T as hot positions. We identified the hot positions of dCBE series single targets to be 18–19 for appA1, 15–20 for appA2, 17–20 for ppsR1, and 19 for ppsR2; the mutation subtype was limited to C-T (Fig. 2e). While we identified the hot positions of nCBE series single targets to be 16–19 for appA1, 14–20 for appA2, 10–20 for ppsR1, and 19 for ppsR2; the dominant mutation subtype was C-T, followed by some C-G and few C-A (Fig. 2f).

Remarkably, we also observed reduced hot positions in double target editing with both dCBE and nCBE series, which demonstrated a narrower activity window compared to single target editing (Additional file 1: Figure S3C, D). Among all above hot positions, we suggested that 14-20 were advisable for a custom target design, and 16-19 were the most preferential since they owned the stably higher mutation frequency.

Lastly, as a case study, we also repeated CBEs with single and double target editing in a non-model microorganism *Rhodobacter sphaeroides* KD131 [27]. According to the sequencing data (Additional file 1: Table S5), it performed well after the first induction, showing the compatibility of pIND4 backbone derived plasmids in other strains.

### nCBE mediated multiplex target editing

**Fig. 3:**
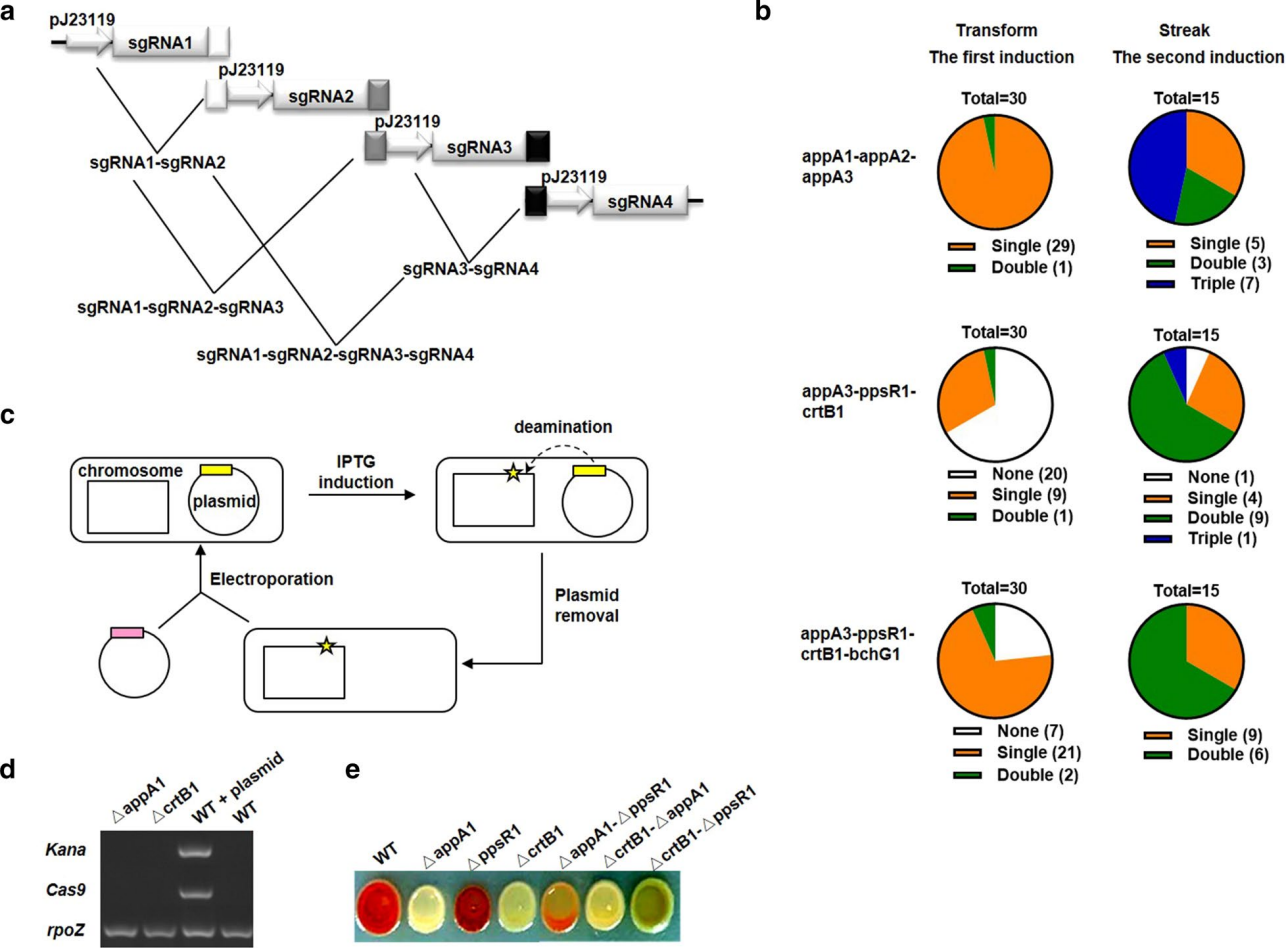
nCBE mediated multiplex and iterative editing. **a** The diagrammatic sketch of multiple sgRNA assembly. White, grey, and black colored box represented the overlap sequence between every two sgRNAs. **b** The pie charts of the screening results for editing triple and quadruple target. Blank, orange, green, and blue colored sector represented the none, single, double, and triple target mutants, respectively. The number in the parentheses meant the matched colonies in each group. **c** The flow chart of plasmid curing. Rectangle, square, and circle represented strain, chromosome, and plasmid, respectively. The yellow and pink colored box on the plasmid represented the sgRNA cassette. Star mark indicated the potential deamination sites. **d** The colony PCR (25 cycles) using the primers binding to the plasmid (*Kana* and *Cas9*) or chromosome (*rpoZ*). **e** The pigment phenotype of the WT, single target mutants (∆appA1, ∆ppsR1, and ∆crtB1), and double target mutants (∆appA1-∆ppsR1, ∆crtB1-∆appA1, and ∆crtB1-∆ppsR1) basing on the genetic background of streaked single target mutant (the plasmid cured ∆appA1 or ∆crtB1 strains)

Given that nCBE could achieve highly efficient single and double target editing where only one-step transformation was needed, we attempted to edit more targets simultaneously. In addition to the well-studied genes *appA* and *ppsR*, we were also able to target a phytoene synthase gene *crtB* (*RSP_0270*) and a chlorophyll synthase gene *bchG* (*RSP_0279*) with high editing efficiency (Additional file 1: Figure S4A) and broad activity window (Additional file 1: Figure S4B). Multiplex target was designed as follows: triple target containing three sgRNAs (appA1–appA2–appA3) located in one gene *appA*; triple target containing three sgRNAs (appA3-ppsR1-crtB1) located in three genes *appA*, *ppsR*, and *crtB*; quadruple target containing four sgRNAs (appA3-ppsR1-crtB1-bchG1) located in four genes *appA*, *ppsR*, *crtB*, and *bchG*. We joined multiple sgRNAs into a fragment with each sgRNA transcribed independently by its own promotor and terminator (Fig. 3a). When editing double target, despite the existence of one-locus mutants (50% for appA1-appA2 and 53.3% for appA1-ppsR2), we also obtained sufficient two-loci mutants (43.3% for appA1-appA2 and 10% for appA1-ppsR2) (Additional file 1: Figure S4C). However, according to the sequencing data (Additional file 1: Table S6), we were only able to obtain a small percentage of two-loci mutants for triple or quadruple target following the first induction step (3.3% for appA1-appA2-appA3, 3.3% for appA3-ppsR1-crtB1, and 6.6% for appA3-ppsR1-crtB1-bchG1). Therefore, we further streaked the one-locus or two-loci mutated colony on a new PYG agar plate (25 μg/ml Kanamycin and 0.5 mM IPTG), to possibly induce all-loci mutations (Additional file 1: Figure S4D).

For triple target appA1-appA2-appA3 and appA3-ppsR1-crtB1, we were able to acquire three-loci mutants after the second induction, with the screening rate of 46.7% and 6.7%, respectively (Fig. 3b). While we were still unable to obtain three-loci or four-loci mutants for quadruple target appA3-ppsR1-crtB1-bchG1 after the second induction, with most colonies (66.7%) just harbored two-loci mutations. As the number of targets adding from zero to four, the transformation efficiency declined dramatically (Additional file 1: Figure S4E). Simultaneous mutation of four or more targets was quite a challenge as very few colonies survived and additional streaking steps required. We recommended iterative editing in order to obtain multiplex target mutated colonies, which relied on the curing of plasmid (Fig. 3c).

In order to verify that the plasmid was removed completely, we designed the primers binding to either plasmid or chromosome for colony PCR. The streaked ∆appA1 and ∆crtB1 strains, similar to the WT strain with no plasmid, displayed no band using primers binding to the plasmid and showed bright band using primers binding to the chromosome (Fig. 3d). We then electroporated a new target plasmid into the above streaked and plasmid cured ∆appA1 and ∆crtB1 strains, generating ∆appA1-∆ppsR1, ∆crtB1-∆appA1, and ∆crtB1-∆ppsR1 strains. Moreover, the pigments of these double target mutants were exactly the mix colors for each single target mutant (Fig. 3e).

Similar to the previously described double target editing processes, the amount of hot positions in triple target mutants decreased, but the hottest positions persisted. This may be explained as the limiting resource competition of base-editor molecules d/nCas9-CDA1-UL among each target. For example, before we planned to edit the triple target appA3-ppsR1-crtB1, we could predict the outcome with hot positions identified for each single target. The hot positions for appA3, ppsR1, and crtB1 were 19, 17 > 15 > 20 > 13 = 10, and 19 > 16, respectively, therefore, we were more likely to get an appA3 (19 C-T)-ppsR1 (17 C-T)-crtB1 (19 C-T) mutant when these three targets were jointed.

### ABEs mediated single target editing

**Fig. 4:**
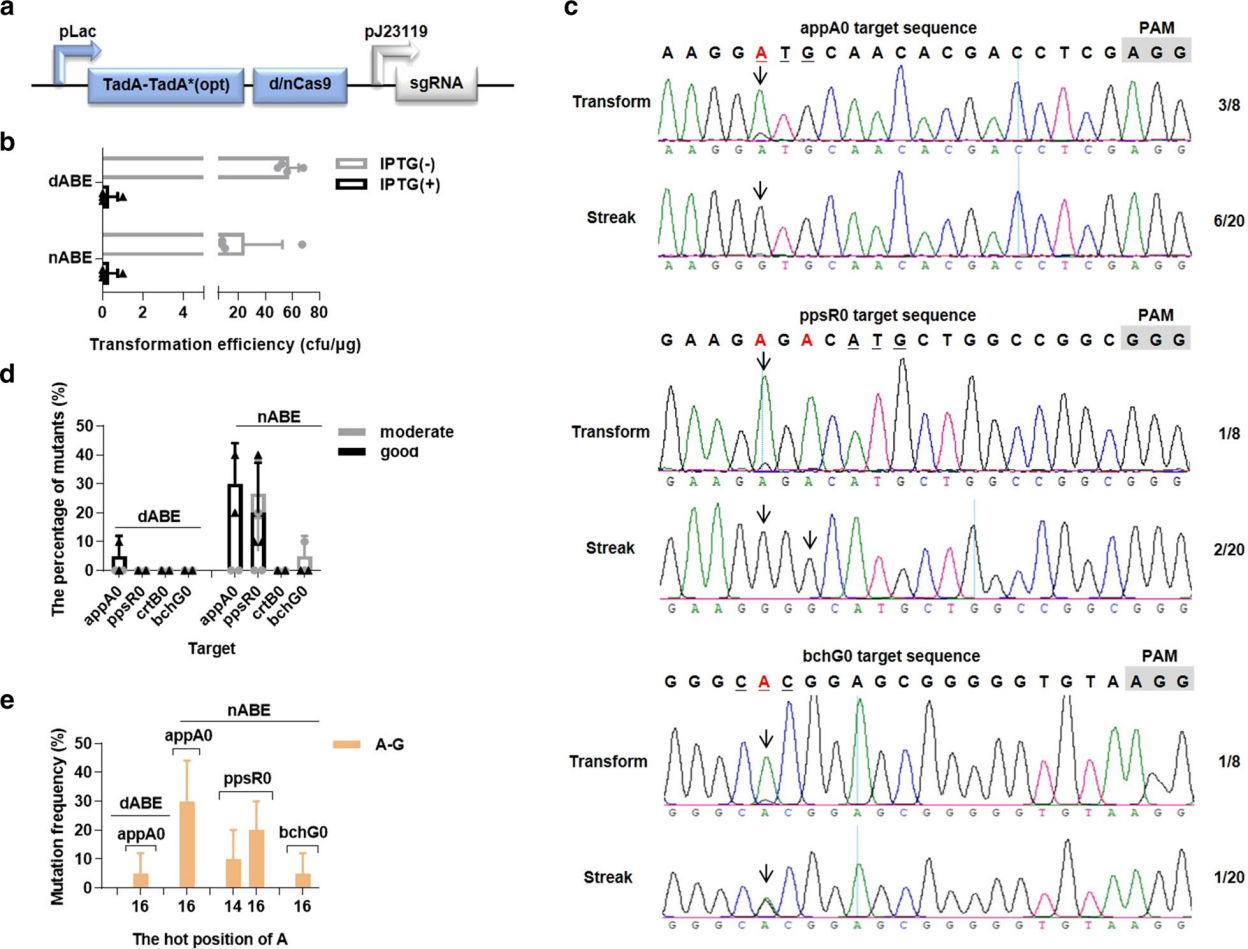
ABEs were lowly or mediumly efficient and caused specific A-G mutation after streak. **a** The diagrammatic sketch of main components in ABEs. **b** The transformation efficiencies of dABE and nABE series single targets, each with 0 mM or 0.5 mM IPTG induction, respectively. **c** The sequencing map of appA0 (upper panel), ppsR0 (middle panel), and bchG0 (below panel) target, with the first induction (transform) and the second induction (streak). The red colored As meant the editable As in the target, and the underlined triplet nucleotides were the start codon for each gene. Black arrows indicated the position where the mutation peaks of A appeared, and the fractional number on the right was the corresponding screening rate. **d** The percentage of good (G) and moderate (G≥A) mutants for dABE or nABE series single targets, respectively. **e** The hot position of A and its mutation frequency for dABE or nABE series single targets, respectively

CRISPR-Cas9 mediated ABEs have successfully been adopted in mammalian and plant cells [28, 29]. However, there have been few finished studies on the induction of A-G in bacteria so far [30, 31]. First, we optimized the nucleotide sequence of the eukaryotic version TadA–TadA* based on the *Rhodobacter sphaeroides* codon-usage table [32]. TadA–TadA* (opt) linked to Cas9 variant (dCas9 or nCas9D10A) as a whole was driven by the inducible promotor pLac, whereas the sgRNA itself was driven by the constitutive promotor pJ23119 (Fig. 4a).

Next, we individually designed a single sgRNA for genes *appA*, *ppsR*, *crtB*, and *bchG* (termed appA0, ppsR0, crtB0, and bchG0, respectively), each located across the start codon. The conversion of either the A in the “ATG” codon or the A opposite the T in the “NTG” codon (underlined letter) to G, would alter the translation level or block the translation initiation of the corresponding genes. For both dABE and nABE series single target, compared to the ~ 100 colonies in control group without IPTG, no more than 10 colonies were survived following the first induction (transform), which indicated a severe threat to the bacterial growth (Fig. 4b).

Then, we picked eight colonies to measure the efficiency of A-G. According to the sequencing data (Additional file 1: Table S7), we failed to get any good (G) or moderate (G≥A) mutant with either dABE or nABE series. But we did see a small proportion of G peaks with nABE series and the percentages of these bad (G<A) mutants were 37.5% for appA0, 12.5% for ppsR0, and 12.5% for bchG0 (Fig. 4c). We then selected two colonies per target for the second induction (streak). After that, we obtained *appA* mutants with the screening rate of 5% using dABE series, and obtained *appA*, *ppsR*, and *bchG* mutants with the screening rate of 30%, 20%, and 5% using nABE series (Fig. 4d).

We saw a narrow activity window of dABE and nABE series at 16 and 14-16, respectively (Fig. 4e). Neither dABE nor nABE series could achieve A-G for crtB0 because the positions of As in this target (3, 11, 12, and 22) were all outside of 14-16. In spite of non-specific mutation subtypes (C-G and C-A) that inevitably occurred by nCBE series, we gained only A-G mutants by both dABE and nABE series, showing their high fidelity.

### Metabolic engineering of the CoQ10 pathway

**Fig. 5:**
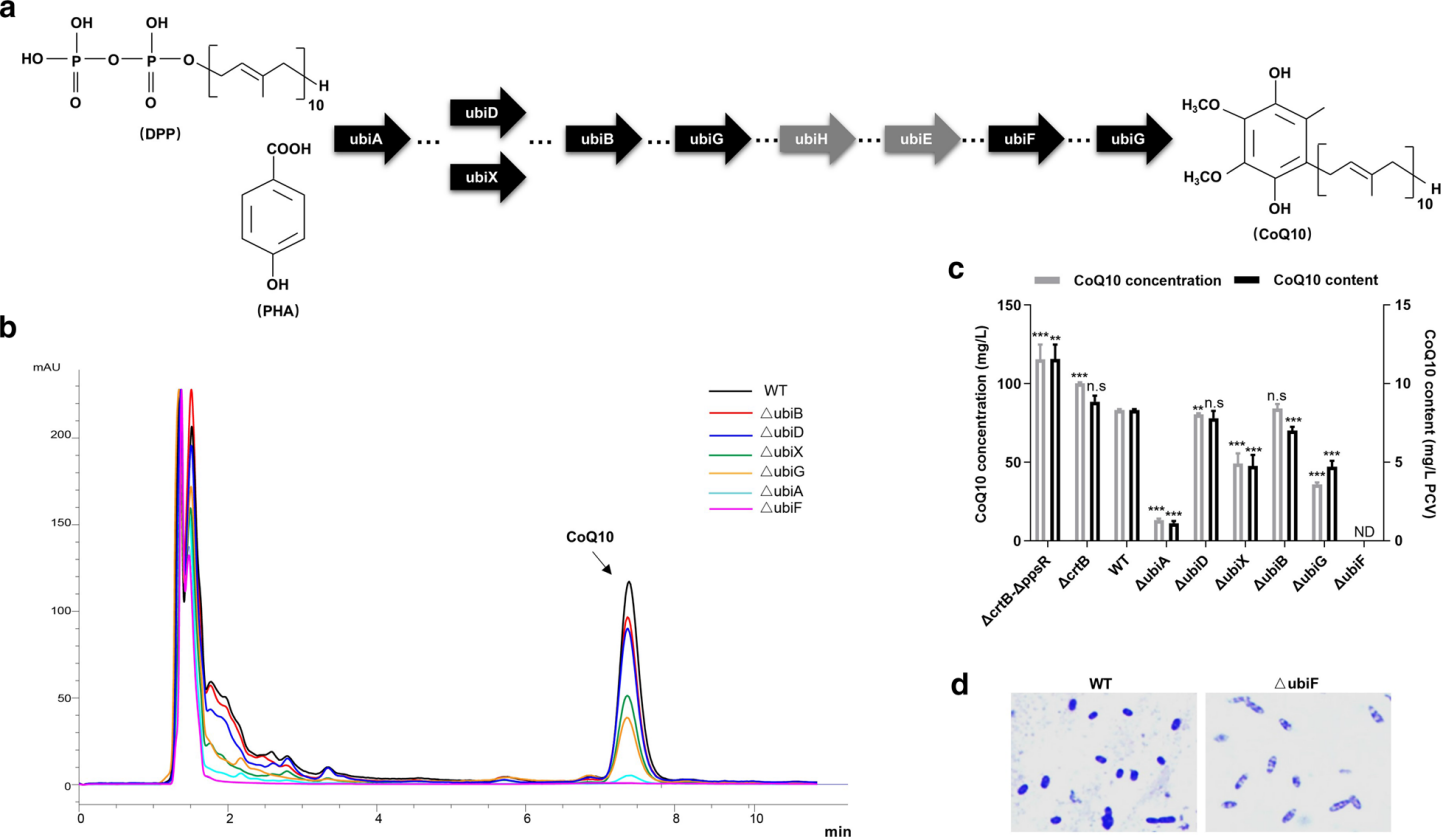
Metabolic engineering of CoQ10 pathway by nCBE series. **a** The biosynthetic route of ubiquinone in *Rhodobacter sphaeroides* 2.4.1, from the precursor decaprenyl diphosphate (DPP) and polyhydroxyalkanoates (PHA) to the final product CoQ10. Black arrows indicated the genes that were successfully knocked out and grey arrows indicated the genes that were failed to be knocked out. **b** The HPLC graphs of the WT and ubiquinone series genes KO mutants. The peaks at the retention time 7.37 min were definitely CoQ10, and the areas of these peaks quantitatively reflected the concentration of each sample. **c** The CoQ10 concentration and content of the tested strains after 5 days’ fermentation. All mutants were compared with the WT and the significant analysis of t-test was shown as (*p < 0.05; **p < 0.01; ***p < 0.001). ND meant none detected and n.s meant no significance. **d** The microscopic pictures of WT and ∆ubiF. The depth of dyeing qualitatively reflected the content of CoQ10

Based on the previous target design criteria, in the whole-genome scale (4287 genes annotated), the coverage of mutable target was 99.8% (4279/4287) or 99.3% (4256/4287) for nCBE or nABE series, respectively; and the coverage of knockout ready target was 96.6% (4140/4287) or 4.2% (178/4287) for nCBE or nABE series, respectively (Additional file 1: Figure S5A). We then attempted to disrupt the related genes of CoQ10 biosynthesis using nCBE series. From the KEGG website, we learned that *ubiA* (*RSP_1008*), *ubiB* (*RSP_1337*), *ubiD* (*RSP_0467*), *ubiE* (*RSP_1338*), *ubiF* (*RSP_1492*), *ubiG* (*RSP_1175*), *ubiH* (*RSP_1869*), and *ubiX* (*RSP_0468*) genes all participated in CoQ10 biosynthesis (Fig. 5a) [33]. Hence, we designed appropriate targets for the above genes, mostly locating in the front part of the gene. To doing so, we obtained KO mutants ΔubiA, ΔubiB, ΔubiD, ΔubiF, ΔubiG, and ΔubiX following the first induction (Additional file 1: Figure S5B). However, we were unable to obtain KO mutants of *ubiE* and *ubiH* genes after many tries, but we did obtain other missense mutation, indicating that these two genes may be essential for strain growth (Additional file 1: Figure S5C).

In order to exclude the influence of the plasmid, we streaked the above ubiquinone series KO mutants onto the PYG agar plate (10% W/V sucrose) before conducting the CoQ10 quantification assay. After 5 days of fermentation, we produced HPLC graphs of all tested samples (Fig. 5b) and determined the order of importance as *ubiF *> *ubiA *> *ubiG *≥ *ubiX *> *ubiD *≥ *ubiB* (putative essential genes *ubiE* and *ubiH* were not listed). The contents of CoQ10 for each KO mutants were also reduced, in particular for ΔubiF, ΔubiA, ΔubiG, and ΔubiX, which dropped to 0%, 13.6%, 56.6%, and 57.2%, respectively (Fig. 5c). Meanwhile, ΔcrtB and ΔcrtB-ΔppsR were set as the positive control, which rose to 106% and 139%, respectively (Additional file 1: Table S8). The microscopic images of WT and each KO mutants were also displayed. Compared to WT, the shape of ΔubiF was long and rod-shaped in the two sides but very empty in the middle (Fig. 5d).

## Discussion

In this study, for the first time, we established and characterized two types of base editors (CBEs and ABEs) in the model strain *Rhodobacter sphaeroides* 2.4.1. These editors were independent, deliverable with electric-pulse, IPTG-inducible, and expressed by all-in-one plasmids. With CBEs, following the first induction step (transform), dCBE enabled mediumly efficient but specific C-T mutation of single target; while nCBE enabled highly efficient C-T mutation of both single or double target, accompanying by non-specific C-G and C-A mutations. After the second induction step (streak), nCBE was even capable of implementing C-T mutation of triple target. The expression of the fusion protein dCas9-CDA1-UL (dCBE) was lower than that of nCas9-CDA1-UL (nCBE), although the detailed mechanism remained to be uncovered, the “expression-dosage” effect might be part of the reason why nCBE series showed higher efficiency than dCBE series. Besides, previous study explained that nCas9 nicked the non-mutated strand during DNA replication, thus accumulated the mutated C-T strand in the next generation strains, which also contributed to the better performance of nCBE series [19]. With ABEs, only after the second induction step (streak) could dABE or nABE enable lowly or mediumly efficient but specific A-G mutation of single target. Compared to the traditional double crossover method, these novel base editors greatly shortened the process duration (from 2 weeks/gene to 1 week/gene), elevated the positive screening rate (from variable 0–40% to stable ~ 100%), increased the throughput (from single gene/round to three genes/round), and eliminated the potential polar effect (from antibiotic marker integration to no-scar left). During the submission of this manuscript, an efficient Cas9-sgRNA based genome editing toolbox was published [34], which enabled knock-out, knock-in, and single nucleotide substitutions in *Rhodobacter sphaeroides* 265-9c (derivative of ATCC 35053). However, the efficiency of this system was fluctuant and spacer-dependent, and it had limited throughout because the cloning of different homologous upstream and downstream arm was required for each target.

Although CoQ10 have been native in *Rhodobacter sphaeroides* 2.4.1 over three decades, its speed-limiting genes are poorly understood [35–38]. With nCBE, we disrupted the ubiquinone series genes involving in CoQ10 biosynthesis and emphasized the importance of *ubiF*, *ubiA*, *ubiG*, and *ubiX*, especially for *ubiF*: CoQ10 content of its KO mutants ΔubiF was diminished to absolutely zero. In addition to simple gene inactivation, it can also be used to optimize gene expression as a new way of metabolic engineering. For example, we can mutate the amino acid sequence of the critical protein based on known conserved active sites; once the translation of these proteins is enhanced, the production of CoQ10 will be increased. It’s also convenient for us to build a genome-scale library and discover other genes related with CoQ10 biosynthetic route (if its KO increases CoQ10 production, it is an inhibitor gene, and vice versa). The genes can be quickly identified by sequencing the sgRNA fragment on the plasmid; for the next round of screening, we just need to add 10% W/V sucrose to remove the plasmid and prepare a new batch of competent cells (Additional file 1: Figure S5D). As single gene may have limited effect on CoQ10 biosynthesis, thereby we hope to see increased productivity in multiple gene mutants. For instance, if we wanted to mutate sextuple target, we could construct two plasmids (each carrying a triple target). We can electroporate the first plasmid into the WT, obtain the three-loci mutants with IPTG induction, streak it under the pressure of 10% W/V sucrose to remove the plasmid. We can then electroporate the second plasmid into the above three-loci mutants and eventually gain the six-loci mutants.

To our knowledge, several papers have reported that CBEs (mostly nCBE) could introduce C-T in bacteria [39–47]. In addition to the C-T triggered by dCBE and nCBE series, we also acquired undesired C-G and C-A using nCBE series. The clear theory of this phenomenon why C-G and C-A will appear is still unknown. According to the Ref. [48], we knew that when the Cs of the target were deaminated to U by CDA1, the uracil-DNA glycosylase (UDG) came to excise it and caused an apurinic/apyrimidinic (AP) sites. At this time, some translesion synthesis (TLS), like Rev1, inserted the C opposite U and AP sites. This might be the probable reason that C-G would happen. Besides, during this process, if other unknown repair enzyme brought T instead of C opposite U and AP sites, the mutation result would be C-A. So, in this study, we rarely got C-G and C-A with dCBE series, we speculated that there was no break on the DNA strand, thus there was very limited space for the enzyme to insert C or T. For dABE and nABE series, no other mutation subtypes (A-T or A-C) aside from A-G was observed, indicating a higher editing specificity. Although ABEs worked well in mammalian cells and plants, there have been few applications in prokaryotes. We supposed that artificially evolved TadA–TadA* maybe toxic to lower organisms (like bacteria), which was short of relevant tolerance mechanism. In the future, we plan to focus on more precisely output of the editing results of CBEs and improving the editing efficiency of ABEs.

## Conclusion

In this work, we developed robust base editing systems by fusing a Cas9 variant with a deaminase. Compared with traditional gene deletion methods, such as Tn5 transposon and double crossover, these CRISPR/Cas9-deaminase based approaches allowed an efficient modification of multiplex targets in a stringent and chemically inducible manner. These CRISPR/Cas9-deaminase based approaches can induce C-T mutation at a single target site and three separate target sites with an editing efficiency of 97% and 47%, and elicit A-G mutation at a single target site with the screening rate of up to 30%, respectively.

According to our best knowledge, it was the first time to achieve C-T and A-G conversion in the model strain *Rhodobacter sphaeroides* 2.4.1 with high efficiency. We also determined *ubiF*, *ubiA*, *ubiG*, and *ubiX* to be the most important genes in CoQ10 biosynthetic pathway using these novel base editors. Together, these results suggested that CBEs and ABEs were powerful genetic manipulation tools in *Rhodobacter sphaeroides*. We believe that CBEs and ABEs can aide in functional gene studies for other strains.

## Supplementary information

**Additional file 1.** Additional figures and tables.

## Data Availability

The datasets supporting the conclusions of this article are included within the article and its additional file.

## References

[1] Barrangou R, Fremaux C, Deveau H, Richards M, Boyaval P, Moineau S, Romero DA, Horvath P (2007). CRISPR provides acquired resistance against viruses in prokaryotes. Science.

[2] Grissa I, Vergnaud G, Pourcel C (2007). The CRISPRdb database and tools to display CRISPRs and to generate dictionaries of spacers and repeats. BMC Bioinf.

[3] Horvath P, Barrangou R (2010). CRISPR/Cas, the immune system of bacteria and archaea. Science.

[4] Sampson TR, Saroj SD, Llewellyn AC, Tzeng YL, Weiss DS (2013). A CRISPR/Cas system mediates bacterial innate immune evasion and virulence. Nature.

[5] Jinek M, Chylinski K, Fonfara I, Hauer M, Doudna JA, Charpentier E (2012). A programmable dual-RNA–guided DNA endonuclease in adaptive bacterial immunity. Science.

[6] Cong L, Ran FA, Cox D, Lin S, Barretto R, Habib N, Hsu PD, Wu X, Jiang W, Marraffini LA, Zhang F (2013). Multiplex genome engineering using CRISPR/Cas systems. Science.

[7] Mali P, Yang L, Esvelt KM, Aach J, Guell M, DiCarlo JE, Norville JE, Church GM (2013). RNA-guided human genome engineering via Cas9. Science.

[8] Makarova KS, Wolf YI, Alkhnbashi OS, Costa F, Shah SA, Saunders SJ, Barrangou R, Brouns SJ, Charpentier E, Haft DH, Horvath P, Moineau S, Mojica FJ, Terns RM, White MF, Yakunin AF, Garrett RA, van der Oost J, Backofen R, Koonin EV (2015). An updated evolutionary classification of CRISPR-Cas systems. Nat Rev Microbiol.

[9] Ran FA, Hsu PD, Wright J, Agarwala V, Scott DA, Zhang F (2013). Genome engineering using the CRISPR-Cas9 system. Nat Protoc.

[10] Hsu PD, Lander ES, Zhang F (2014). Development and applications of CRISPR-Cas9 for genome engineering. Cell.

[11] Mougiakos I, Bosma EF, de Vos WM, van Kranenburg R, van der Oost J (2016). Next generation prokaryotic engineering: the CRISPR-Cas toolkit. Trends Biotechnol.

[12] Selle K, Barrangou R (2015). Harnessing CRISPR–Cas systems for bacterial genome editing. Trends Microbiol.

[13] Suwanto A, Kaplan S (1989). Physical and genetic mapping of the *Rhodobacter sphaeroides* 2.4.1 genome: presence of two unique circular chromosomes. J Bacteriol..

[14] Zou RS, Li S, Zhang LL, Zhang C, Han YJ, Gao G, Sun X, Gong X (2019). Mutagenesis of *Rhodobacter sphaeroides* using atmospheric and room temperature plasma treatment for efficient production of coenzyme Q10. J Biosci Bioeng.

[15] Coomber SA, Chaudhri M, Connor A, Britton G, Hunter CN (1990). Localized transposon Tn5 mutagenesis of the photosynthetic gene cluster of *Rhodobacter sphaeroides*. Mol Microbiol.

[16] Schäfer A, Tauch A, Jager W, Kalinowski J, Thierbach G, Puhler A (1994). Small mobilizable multi-purpose cloning vectors derived from the *Escherichia coli* plasmids pK18 and pK19: selection of defined deletions in the chromosome of *Corynebacterium glutamicum*. Gene.

[17] Kontur WS, Schackwitz WS, Ivanova N, Martin J, Labutti K, Deshpande S, Tice HN, Pennacchio C, Sodergren E, Weinstock GM, Noguera DR, Donohue TJ (2012). Revised sequence and annotation of the *Rhodobacter sphaeroides* 2.4.1 genome. J Bacteriol..

[18] Komor AC, Kim YB, Packer MS, Zuris JA, Liu DR (2016). Programmable editing of a target base in genomic DNA without double-stranded DNA cleavage. Nature.

[19] Nishida K, Arazoe T, Yachie N, Banno S, Kakimoto M, Tabata M, Mochizuki M, Miyabe A, Araki M, Hara KY, Shimatani Z, Kondo A (2016). Targeted nucleotide editing using hybrid prokaryotic and vertebrate adaptive immune systems. Science..

[20] Esvelt KM, Carlson JC, Liu DR (2011). A system for the continuous directed evolution of biomolecules. Nature.

[21] Gaudelli NM, Komor AC, Rees HA, Packer MS, Badran AH, Bryson DI, Liu DR (2017). Programmable base editing of A·T to G·C in genomic DNA without DNA cleavage. Nature.

[22] Ind AC, Porter SL, Brown MT, Byles ED, de Beyer JA, Godfrey SA, Armitage JP (2009). Inducible-expression plasmid for *Rhodobacter sphaeroides* and *Paracoccus denitrificans*. Appl Environ Microbiol.

[23] Jiang W, Bikard D, Cox D, Zhang F, Marraffini LA (2013). RNA-guided editing of bacterial genomes using CRISPR-Cas systems. Nat Biotechnol.

[24] Vermeulen AJ, Bauer CE (2015). Members of the PpaA/AerR antirepressor family bind cobalamin. J Bacteriol.

[25] Serdyuk OP, Smolygina LD, Chekunova EM, Sannikova EP, Shirshikova GN, Khusnutdinova AN, Yartseva NV (2013). Direct transition of pGA482: ipt plasmid bearing the cytokinin biosynthesis gene into the cells of phototrophic purple bacteria *Rhodobacter sphaeroides* and *Rhodopseudomonas palustris* by electroporation. Doki Biochem Biophys..

[26] Masuda S, Bauer CE (2002). AppA is a blue light photoreceptor that antirepresses photosynthesis gene expression in *Rhodobacter sphaeroides*. Cell.

[27] Kim MS, Kim DH, Cha J, Lee JK (2012). Effect of carbon and nitrogen sources on photo-fermentative H_2_ production associated with nitrogenase, uptake hydrogenase activity, and PHB accumulation in *Rhodobacter sphaeroides* KD131. Bioresour Technol.

[28] Li C, Zong Y, Wang Y, Jin S, Zhang D, Song Q, Zhang R, Gao C (2018). Expanded base editing in rice and wheat using a Cas9-adenosine deaminase fusion. Genome Biol.

[29] Kang BC, Yun JY, Kim ST, Shin Y, Ryu J, Choi M, Woo JW, Kim JS (2018). Precision genome engineering through adenine base editing in plants. Nat Plants..

[30] Tong Y, Whitford CM, Robertsen HL, Blin K, Jørgensen TS, Klitgaard AK, Gren T, Jiang X, Weber T, Lee SY (2019). Highly efficient DSB-free base editing for *Streptomycetes* with CRISPR-BEST. Proc Natl Acad Sci USA.

[31] Xin X, Li J, Zhao D, Li S, Xie Q, Li Z, Fan F, Bi C, Zhang X (2019). Double-check base editing (DBE) for efficient A to G conversions. ACS Synth Biol..

[32] Nakamura Y, Gojobori T, Ikemura T (2000). Codon usage tabulated from the international DNA sequence databases: status for the year 2000. Nucleic Acids Res.

[33] Ogata H, Goto S, Sato K, Fujibuchi W, Bono H, Kanehisa M (1999). KEGG: Kyoto encyclopedia of genes and genomes. Nucleic Acids Res.

[34] Mougiakos I, Orsi E, Ghiffary MR, Post W, de Maria A, Adiego-perez B, Kengen SWM, Weusthuis RA, van der Oost J (2019). Efficient Cas9-based genome editing of *Rhodobacter sphaeroides* for metabolic engineering. Microb Cell Fact.

[35] Lu W, Ye L, Xu H, Xie W, Gu J, Yu H (2014). Enhanced production of Coenzyme Q10 by self-regulating the engineered MEP pathway in *Rhodobacter sphaeroides*. Biotechnol Bioeng.

[36] Lu W, Ye L, Lv X, Xie W, Gu J, Chen Z, Zhu Y, Li A, Yu H (2015). Identification and elimination of metabolic bottlenecks in the quinone modification pathway for enhanced coenzyme Q10 production in *Rhodobacter sphaeroides*. Metab Eng.

[37] Zhu Y, Ye L, Chen Z, Hu W, Shi Y, Chen J, Wang C, Li Y, Li W, Yu H (2017). Synergic regulation of redox potential and oxygen uptake to enhance production of coenzyme Q10 in *Rhodobacter sphaeroides*. Enzyme Microb Technol.

[38] Chen X, Jiang X, Xu M, Zhang M, Huang J, Qi F (2019). Co-production of farnesol and coenzyme Q10 from metabolically engineered *Rhodobacter sphaeroides*. Microb Cell Fact.

[39] Yang L, Briggs AW, Chew WL, Mali P, Guell M, Aach J, Goodman DB, Cox D, Kan Y, Lesha E, Soundararajan V, Zhang F, Church G (2016). Engineering and optimizing deaminase fusions for genome editing. Nat Commun..

[40] Banno S, Nishida K, Arazoe T, Mitsunobu H, Kondo A (2018). Deaminase-mediated multiplex genome editing in *Escherichia coli*. Nat Microbiol..

[41] Zheng K, Wang Y, Li N, Jiang FF, Wu CX, Liu F, Chen HC, Liu ZF (2018). Highly efficient base editing in bacteria using a Cas9-cytidine deaminase fusion. Commun Biol..

[42] Chen W, Zhang Y, Zhang Y, Pi Y, Gu T, Song L, Wang Y, Ji Q (2018). CRISPR/Cas9-based genome editing in *Pseudomonas aeruginosa* and cytidine deaminase-mediated base editing in *Pseudomonas* species. iScience.

[43] Gu T, Zhao S, Pi Y, Chen W, Chen C, Liu Q, Li M, Han D, Ji Q (2018). Highly efficient base editing in *Staphylococcus aureus* using an engineered CRISPR RNA-guided cytidine deaminase. Chem Sci..

[44] Wang Y, Liu Y, Liu J, Guo Y, Fan L, Ni X, Zheng X, Wang M, Zheng P, Sun J, Ma Y (2018). MACBETH: multiplex automated *Corynebacterium glutamicum* base editing method. Metab Eng.

[45] Wang Y, Wang S, Chen W, Song L, Zhang Y, Shen Z, Yu F, Li M, Ji Q (2018). CRISPR-Cas9 and CRISPR-assisted cytidine deaminase enable precise and efficient genome editing in *Klebsiella pneumoniae*. Appl Environ Microbiol.

[46] Li Q, Seys FM, Minton NP, Yang J, Jiang Y, Jiang W, Yang S (2019). CRISPR-Cas9D10A nickase-assisted base editing in the solvent producer *Clostridium beijerinckii*. Biotechnol Bioeng.

[47] Wang Y, Wang Z, Chen Y, Hua X, Yu Y, Ji Q (2019). A highly efficient CRISPR-Cas9-based genome engineering platform in *Acinetobacter baumannii* to understand the H_2_O_2_-sensing mechanism of OxyR. Cell Chem Biol..

[48] Gajula KS (2019). Designing an elusive C·G→G·C CRISPR base editor. Trends Biochem Sci.

