# Supplementary material for: CRISPR/Cas9-deaminase enables robust base editing in Rhodobacter sphaeroides 2.4.1
Source: Microb Cell Fact. 2020 Apr 25;19:93. doi: 10.1186/s12934-020-01345-w (PMC7183636; doi:10.1186/s12934-020-01345-w)
Supplement: Supplementary file 1 — Additional file 1. Additional figures and tables. [file 12934_2020_1345_MOESM1_ESM.docx]

**Additional file 1**

**CRISPR/Cas9-deaminase enables robust base editing in** ***Rhodobacter sphaeroides* 2.4.1**

Yufeng Luo^1^, Mei Ge^2^, Bolun Wang^3^, Changhong Sun^4^, Junyi Wang^3^, Yuyang Dong^5^, Jianzhong Jeff Xi^1,3*^

^1^ *State Key Laboratory of Biomembrane and Membrane Biotechnology, Institute of Molecular Medicine, Peking University, Beijing 100871, China.*

^2^ *Shanghai Laiyi Center for Biopharmaceutical R&D, 800 Dongchuan Road, Shanghai 200240, China*

^3^ *Department of Biomedical Engineering, State Key Laboratory of Natural and Biomimetic Drugs, College of Engineering, Peking University, Beijing 100871, China.*

^4^ *Beijing Viewsolid Biotech Co. Ltd, Beijing 100071, China.*

^5^ *Wallace H. Coulter Department of Biomedical Engineering, Georgia Institute of Technology and Emory University, 313 Ferst Drive NW, Atlanta, GA 30332, USA.*

* Correspondence: jzxi@pku.edu.cn


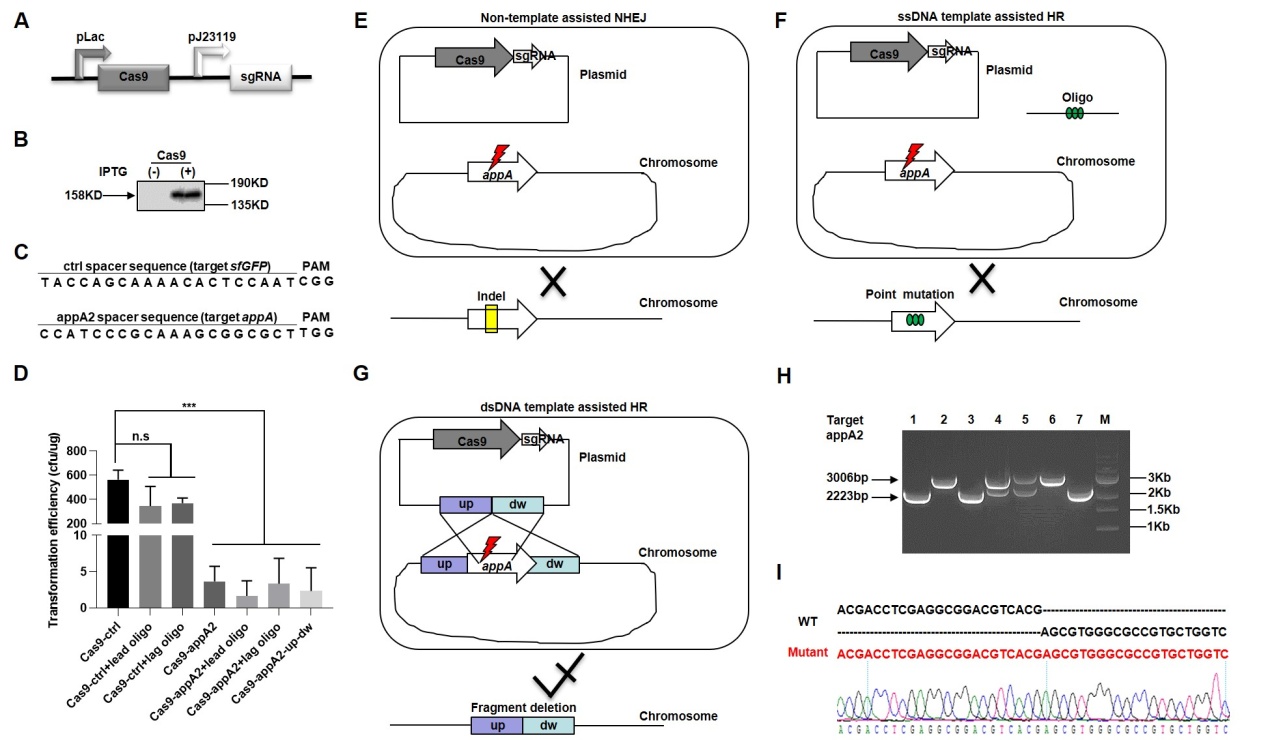


**Figure S1.** Cas9-sgRNA system mediated genome editing in *Rhodobacter sphaeroides* 2.4.1. **A** The diagrammatic sketch of main components in Cas9-sgRNA system. **B** The western blot of Cas9, with 0mM or 0.5mM IPTG induction, respectively. **C** The 20bp spacer sequence of the control sgRNA ctrl and the targeting sgRNA appA2. **D** The transformation efficiency of each experiment group (plasmid only, plasmid plus oligo, and plasmid containing homologous arms). The significant analysis of t-test was shown as (p<0.05 *; p<0.01 **; p<0.001 ***), and n.s meant no significance. **E** The non-template assisted NHEJ was failed. **F** The ssDNA oligo assisted HR was failed. **G** The dsDNA plasmid assisted HR was successful but with a fluctuation efficiency. **H** The colony PCR (25 cycles) for screening *appA* gene deletion mutants (appA2 target). Lane 1, Lane 3, and Lane 7 were pure mutant; Lane 4 and Lane 5 were mixture; Lane 2 and Lane 6 were WT. The size of amplification band for WT and mutant was 3006bp and 2223bp, respectively. M: 1Kb DNA Ladder marker. **I** The sequencing map of the WT and mutant.


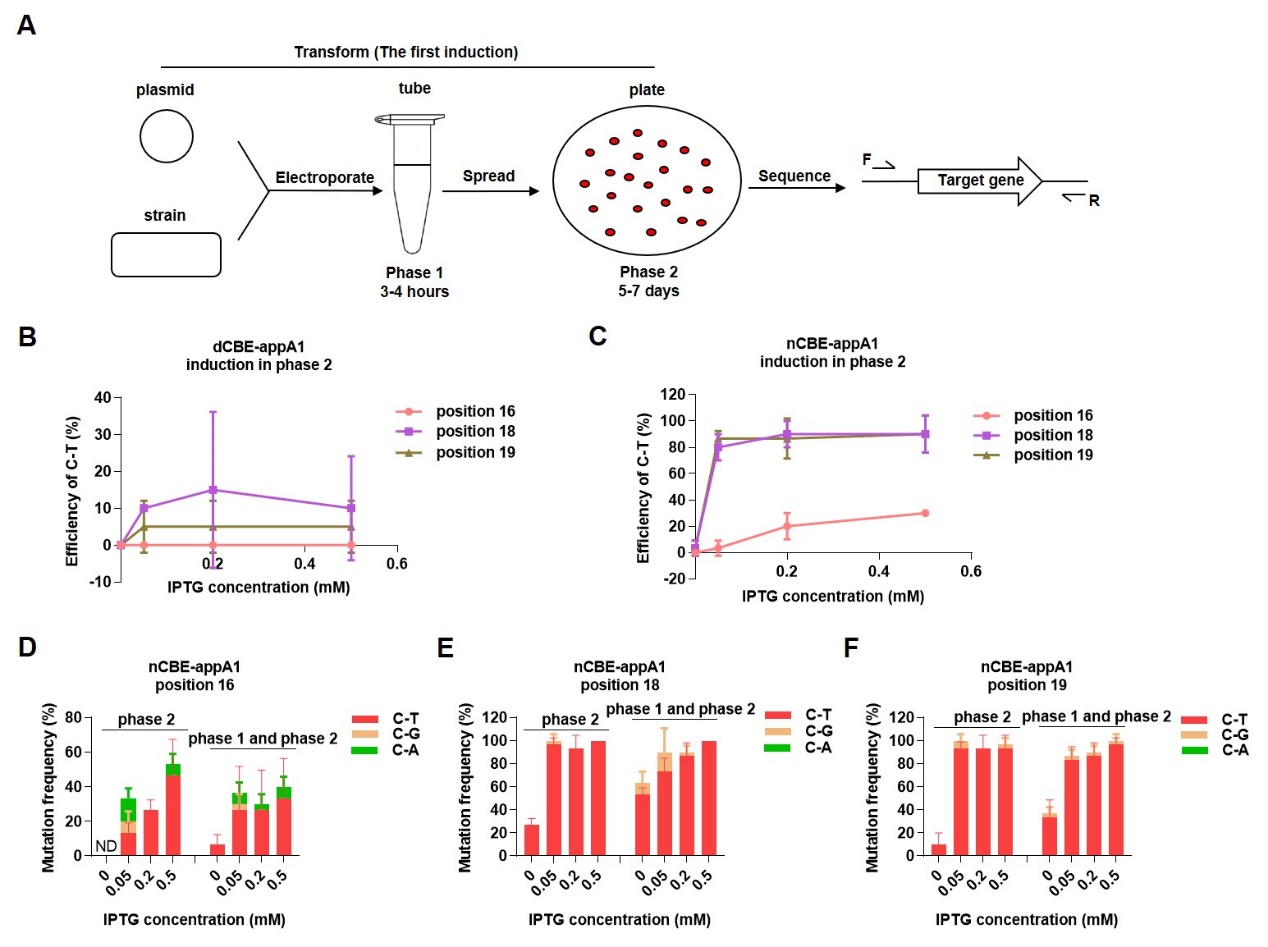


**Figure S2.** The protocol, efficiency, and non-specific mutation subtypes of CBEs. **A** The flow chart of electric-pulsed transform step. The plasmid was directly electroporated into the strain. The sequencing primer set was designed flanking the target gene. **B-C** After plasmid dCBE-appA1 or nCBE-appA1 was delivered, the efficiency of C-T for the Cs at position 16, 18, and 19 in appA1 target were individually calculated, with the IPTG induction of various concentration ranging from 0mM to 0.5mM in only phase 2. **D-F** After plasmid nCBE-appA1 was delivered, the mutation frequency for the Cs at position 16, 18, and 19 in appA1 target were calculated, with the IPTG induction of various concentration ranging from 0mM to 0.5mM in only phase 2; or with the IPTG induction of constant concentration 0.5mM in phase 1 and various concentration ranging from 0mM to 0.5mM in phase 2. ND meant none detected.


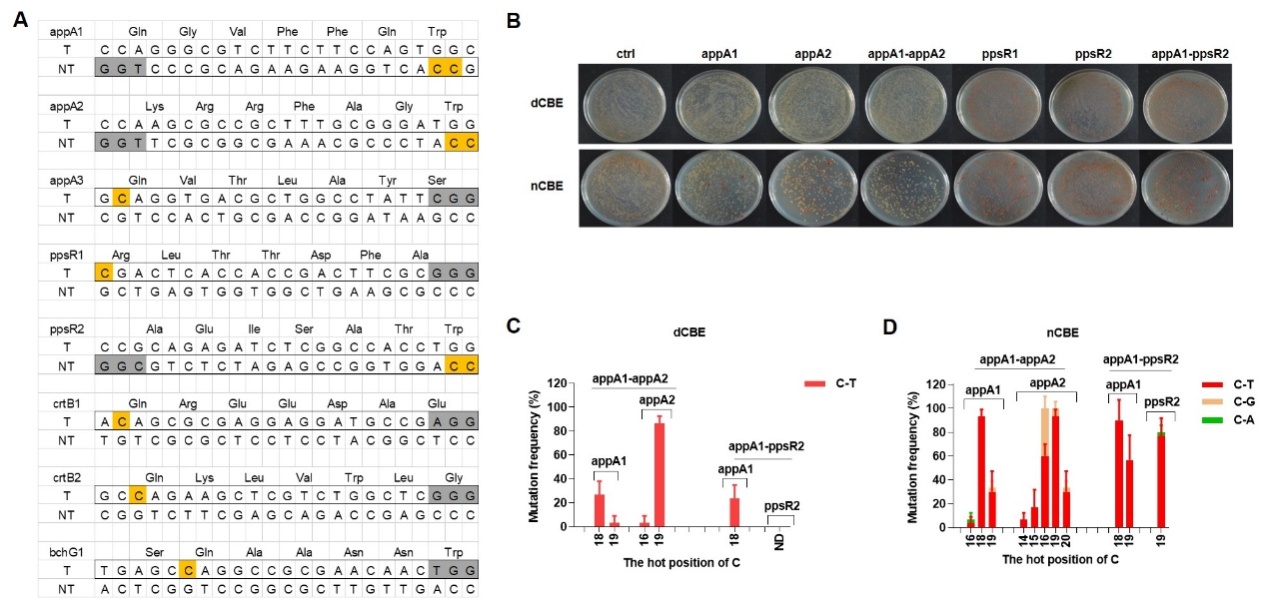


**Figure S3.** The other editing features of dCBE and nCBE series. **A** The target sequence designed for assessing C-T efficiency in this study. The T and NT meant template and non-template strand, respectively. The grey shadow part represented the PAM sequence, and the yellow shadow part indicated the key nucleotide that may be mutated to produce stop codon. **B** The PYG agar plates of dCBE and nCBE series targets. **C-D** The hot position of C and its mutation frequency for dCBE and nCBE series double targets (appA1-appA2 and appA1-ppsR2), respectively. ND meant none detected.


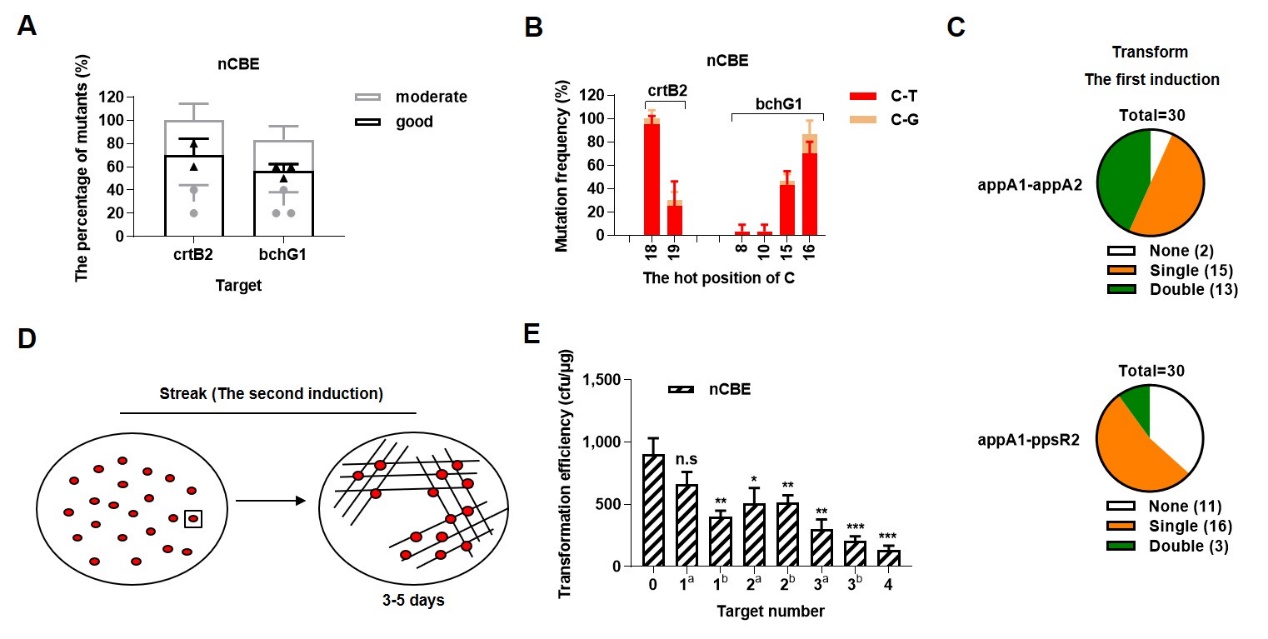


**Figure S4.** nCBE enabled at most triple target editing, which required the additional streak step. **A** The percentage of good and moderate mutants for nCBE series crtB2 and bchG1 targets. **B** The hot position of C and its mutation frequency for nCBE series crtB2 and bchG1 targets. **C** The pie charts of the screening results for editing nCBE series double target appA1-appA2 and appA1-ppsR2. Blank, orange, and green sector meant the none, single, and double target mutant, respectively. **D** The procedure of streak step, which required about 3-5 days’ incubation until the colonies were visible. **E** The transformation efficiency of nCBE series targets (ranging from zero to four). 0, 1^a^, 1^b^, 2^a^, 2^b^, 3^a^, 3^b^, and 4 represented ctrl, ppsR2, appA1, appA1-ppsR2, appA1-crtB2, appA1-appA2-appA3, appA3-ppsR1-crtB1, and appA3-ppsR1-crtB1-bchG1 target, respectively. The significant analysis of t-test was shown as (p<0.05 *; p<0.01 **; p<0.001 ***), and n.s meant no significance.


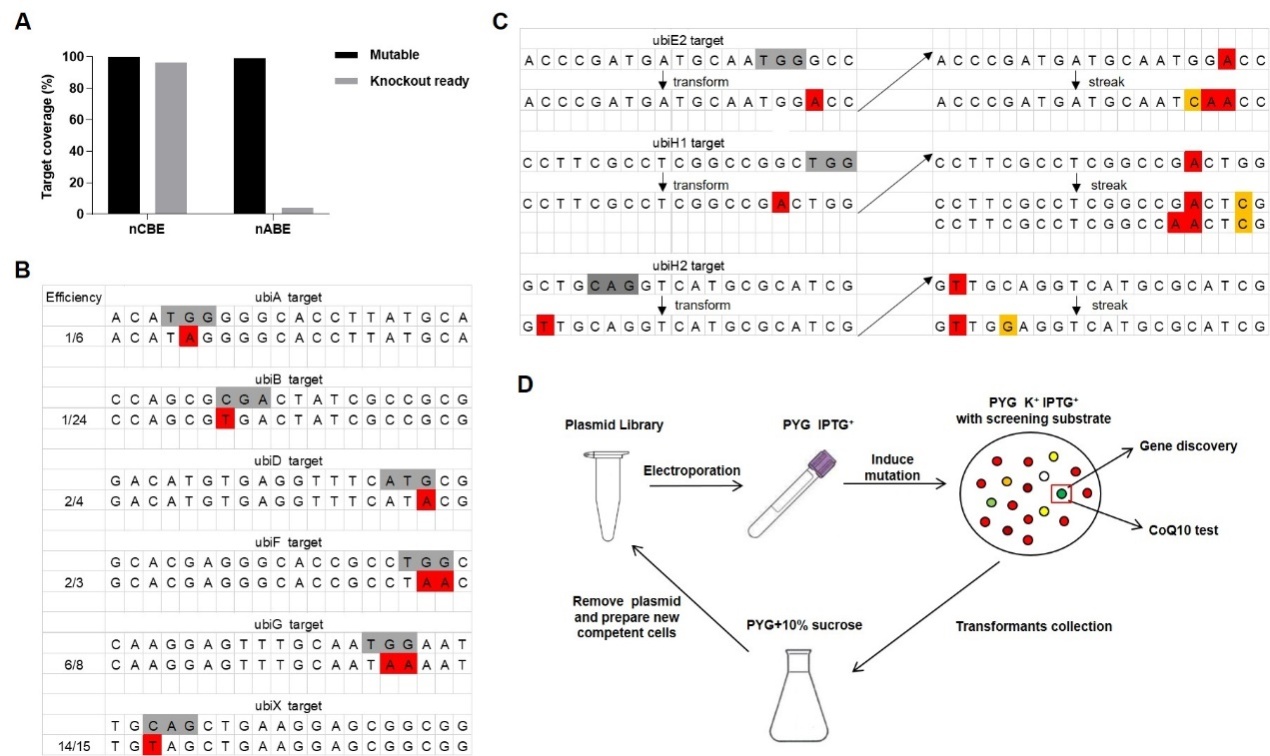


**Figure S5.** The target scope of CBEs and ABEs, genotype of ubiquinone series gene mutants, and the presumed high-throughput screening platform. **A** The genome-scale target coverage analysis of *Rhodobacter sphaeroides* 2.4.1 for nCBE and nABE series, respectively. **B** The genotype of *ubiA*, *ubiB*, *ubiD*, *ubiF*, *ubiG*, and *ubiX* KO mutants and its corresponding screening rate. The grey shadow represented the triplet codon that might become premature stop codon. **C** The genotype of *ubiE* and *ubiH* missense mutants, which was sequenced after the first induction (transform) and the second induction (streak), respectively. The grey shadow represented the triplet codon that might become premature stop codon, but the obtained mutants were all the escapers that bypassed the stop codon. **D** One round of iterative screening for the high-yield CoQ10 candidates. Plasmid library was delivered to the WT strain, and IPTG was added in order to induce the mutation. Under the pressure of screening substrates, the transformants were either tested for CoQ10 assay or prepared as a new batch of competent cell for the next round of screening.

Table S1 Primers used for plasmid construction

| Primer name | Sequence (5’-3’) (underlined letter represented the target sequence) |
| --- | --- |
| Cas9-F | GGAGAAATTAACCATGGTAACCGGTATGGATAAGAAATACTCAATAGGC |
| Cas9-R | TTATCCGCTCACAAGTCAAACTAGTTCAGTCACCTCCTAGCTGACTCAAATC |
| sgRNA-2F | ATACTTAGATTCTCACACAGTCGACTTGACAGCTAGCTCAGTCCTAGGTATAATACTAGT |
| appA2-1F | GCTCAGTCCTAGGTATAATACTAGTCCATCCCGCAAAGCGGCGCTGTTTTAGAGCTAGAAATAGC |
| ctrl-1F | GCTCAGTCCTAGGTATAATACTAGTTACCAGCAAAACACTCCAATGTTTTAGAGCTAGAAATAGC |
| sgRNA-R | AGCTCAGCTAATTAAGCTTAGATCTCTCGAGAAAAAAGCACCGACTC |
| appA-up-F | CGTTTTTTATTGGTGAGAATCCAAGGGGCCGCGAAGAAGGGCAGGC |
| appA-up-R | GACCAGCACGGCGCCCACGCTCGTGACGTCCGCCTCGAGGTC |
| appA-dw-F | GACCTCGAGGCGGACGTCACGAGCGTGGGCGCCGTGCTGGTC |
| appA-dw-R | CTTAGCTCCTGAAAATCTCGCCAAGGACCCAGGGCCCCACATGCGG |
| appA2-lead-88nt | G*G*C*GCCACAGCAACGTCGAGATCCTCGCAGAGGAACCGATCTGATAGCGCCGCTTTGCGGGATGGCACATGCAGCTCTCCTGCTC*G*G*A (* meant 5’ and 3’ phosphonothioate modified bond) |
| appA2-lag-88nt | T*C*C*GAGCAGGAGAGCTGCATGTGCCATCCCGCAAAGCGGCGCTATCAGATCGGTTCCTCTGCGAGGATCTCGACGTTGCTGTGGC*G*C*C (* meant 5’ and 3’ phosphonothioate modified bond) |
| MCS-1F | CATGGTAACCGGTGACTACTAGTTTGACTTGTGAGCGGATA |
| MCS-1R | TTGTTATCCGCTCACAAGTCAAACTAGTAGTCACCGGTTAC |
| MCS-2F | ACAATGATACTTAGATTCTCACACAGTCGACAGTCAGATCTA |
| MCS-2R | AGCTTAGATCTGACTGTCGACTGTGTGAGAATCTAAGTATCA |
| SacB-F | GGGCGAAGAAGTTGTCCATATTGGCACATATACCTGCCGTTCAC |
| SacB-R | TCACCAGTTTTGATTTAAACGTGGTCATTTGTTAACTGTTAATTGTC |
| CBE-dCas9(nCas9D10A)-F | GGAGAAATTAACCATGGTAACCGGTATGGATAAGAAATACTCAATAGGC |
| CBE-dCas9(nCas9D10A)-R | CACCTCCAGAACCTCCTCCACCGTCACCTCCTAGCTGACTCAAATC |
| Linker-F | GGTGGAGGAGGTTCTGGAGGTG |
| Linker-R | CCTAGACTTATCGTCATCGTC |
| CDA1-F | GACGATGACGATAAGTCTAGGATGACCGACGCTGAGTACGTG |
| CDA1-R | AACAGCAGGACTCTTAGTGGTG |
| UGI-F | CACCACTAAGAGTCCTGCTGTTTCTAGAATGACCAACCTTTCCGACATC |
| UGI-R | TTATCCGCTCACAAGTCAAACTAGTTCATGCAACCAGTCCTAGCATCTTG |
| CBE-sgRNA-2F | AAGATGCTAGGACTGGTTGCATGAACTAGTTTGACAGCTAGCTCAGTCCTAGGTATAATACTAGT |
| CBE-appA1-1F | GCTCAGTCCTAGGTATAATACTAGTGCCACTGGAAGAAGACGCCCGTTTTAGAGCTAGAAATAGC |
| CBE-appA2-1F | GCTCAGTCCTAGGTATAATACTAGTCCATCCCGCAAAGCGGCGCTGTTTTAGAGCTAGAAATAGC |
| CBE-appA3-1F | GCTCAGTCCTAGGTATAATACTAGTGCAGGTGACGCTGGCCTATTGTTTTAGAGCTAGAAATAGC |
| CBE-ppsR1-1F | GCTCAGTCCTAGGTATAATACTAGTCGACTCACCACCGACTTCGCGTTTTAGAGCTAGAAATAGC |
| CBE-ppsR2-1F | GCTCAGTCCTAGGTATAATACTAGTCCAGGTGGCCGAGATCTCTGGTTTTAGAGCTAGAAATAGC |
| CBE-crtB1-1F | GCTCAGTCCTAGGTATAATACTAGTACAGCGCGAGGAGGATGCCGGTTTTAGAGCTAGAAATAGC |
| CBE-crtB2-1F | GCTCAGTCCTAGGTATAATACTAGTGCCAGAAGCTCGTCTGGCTCGTTTTAGAGCTAGAAATAGC |
| CBE-bchG1-1F | GCTCAGTCCTAGGTATAATACTAGTTGAGCCAGGCCGCGAACAACGTTTTAGAGCTAGAAATAGC |
| CBE-ctrl-1F | GCTCAGTCCTAGGTATAATACTAGTTACCAGCAAAACACTCCAATGTTTTAGAGCTAGAAATAGC |
| CBE-BspQI-1F | GCTCAGTCCTAGGTATAATACTAGTTGAAGAGCAGCTGCTCTTCAGTTTTAGAGCTAGAAATAGC |
| CBE-ubiA-1F | GCTCAGTCCTAGGTATAATACTAGTTGCATAAGGTGCCCCCATGTGTTTTAGAGCTAGAAATAGC |
| CBE-ubiB-1F | GCTCAGTCCTAGGTATAATACTAGTCCAGCGCGACTATCGCCGCGGTTTTAGAGCTAGAAATAGC |
| CBE-ubiD-1F | GCTCAGTCCTAGGTATAATACTAGTCGCATGAAACCTCACATGTCGTTTTAGAGCTAGAAATAGC |
| CBE-ubiE1-1F | GCTCAGTCCTAGGTATAATACTAGTCGTGCAGGACGCGCTGAACGGTTTTAGAGCTAGAAATAGC |
| CBE-ubiE2-1F | GCTCAGTCCTAGGTATAATACTAGTGGCCCATTGCATCATCGGGTGTTTTAGAGCTAGAAATAGC |
| CBE-ubiE3-1F | GCTCAGTCCTAGGTATAATACTAGTCCAGAAGCTCCTCGATGTGGGTTTTAGAGCTAGAAATAGC |
| CBE-ubiF-1F | GCTCAGTCCTAGGTATAATACTAGTGCCAGGCGGTGCCCTCGTGCGTTTTAGAGCTAGAAATAGC |
| CBE-ubiG-1F | GCTCAGTCCTAGGTATAATACTAGTATTCCATTGCAAACTCCTTGGTTTTAGAGCTAGAAATAGC |
| CBE-ubiH1-1F | GCTCAGTCCTAGGTATAATACTAGTCCAGCCGGCCGAGGCGAAGGGTTTTAGAGCTAGAAATAGC |
| CBE-ubiH2-1F | GCTCAGTCCTAGGTATAATACTAGTGCTGCAGGTCATGCGCATCGGTTTTAGAGCTAGAAATAGC |
| CBE-ubiX-1F | GCTCAGTCCTAGGTATAATACTAGTTGCAGCTGAAGGAGCGGCGGGTTTTAGAGCTAGAAATAGC |
| CBE-sgRNA-R | AGCTAATTAAGCTTAGATCTGACTGTCGACCTCGAGAAAAAAGCACCGACTC |
| TadA-F | TTAAAGAGGAGAAATTAACCATGGCCGCAGTGTCTTGCGTCTCT |
| TadA-R | CAAGTCAAACTAGTAGTCACCGGTGTTGGCAGTGACTCCGTCTCT |
| TadA-dCas9(nCas9D10A)-F | CGGGCGGTTCGTCGGGTGGCTCTGATAAGAAATACTCAATAGGC |
| TadA-dCas9(nCas9D10A)-R | TTATCCGCTCACAAGTCAAACTAGTTCAGTGGTGATGATGGTGATGGTCACCTCCTAGCTGACTCAAAT |
| ABE-sgRNA-2F | TAGATTCTCACACAGTCGACAGTCATTGACAGCTAGCTCAGTCCTAGGTATAATACTAGT |
| ABE-appA0-1F | GCTCAGTCCTAGGTATAATACTAGTAAGGATGCAACACGACCTCGGTTTTAGAGCTAGAAATAGC |
| ABE-ppsR0-1F | GCTCAGTCCTAGGTATAATACTAGTGAAGAGACATGCTGGCCGGCGTTTTAGAGCTAGAAATAGC |
| ABE-crtB0-1F | GCTCAGTCCTAGGTATAATACTAGTTCATTCCGCGGCAAGCCTTTCAGCGTTTTAGAGCTAGAAATAGC |
| ABE-bchG0-1F | GCTCAGTCCTAGGTATAATACTAGTGGGCACGGAGCGGGGGTGTAGTTTTAGAGCTAGAAATAGC |
| ABE-ctrl-1F | GCTCAGTCCTAGGTATAATACTAGTTACCAGCAAAACACTCCAATGTTTTAGAGCTAGAAATAGC |
| ABE-sgRNA-R | GTCCAAGCTCAGCTAATTAAGCTTACTCGAGAAAAAAGCACCGACTC |

Table S2 Primers used for Sanger sequencing

| Plasmid | Target sequence (PAM is underlined) | Primer Sequence (5’-3’) |
| --- | --- | --- |
| pIND4-Cas9-appA2  pIND4-Cas9-appA2-up-dw | CCATCCCGCAAAGCGGCGCTTGG | appA-del-F AGGCCCACCGGCTCCAGAAC |
|  |  | appA-del-R GATGTAGGTCCGCCACTTCTC |
| d/nCBE-appA1 | GCCACTGGAAGAAGACGCCCTGG | appA-F GTCTCGTCCATCCGTCCCTCCCTT  appA-R GCCCAGCCTGTCTGTTCTCGTCTC |
| d/nCBE-appA2 | CCATCCCGCAAAGCGGCGCTTGG |  |
| nCBE-appA3 | GCAGGTGACGCTGGCCTATTCGG |  |
| d/nCBE-ppsR1 | CGACTCACCACCGACTTCGCGGG | ppsR-F GCCATAATGATTTCCCTTGCC  ppsR-R GGAACATGAAGGTGGCGTGGAT |
| d/nCBE-ppsR2 | CCAGGTGGCCGAGATCTCTGCGG |  |
| nCBE-crtB1 | ACAGCGCGAGGAGGATGCCGAGG | crtB-F GGGCTGTTCGTCTGGTATTTCGG  crtB-R GAAGAAGACCGGCGTCCAGAGC |
| nCBE-crtB2 | GCCAGAAGCTCGTCTGGCTCGGG |  |
| nCBE-bchG1 | TGAGCCAGGCCGCGAACAACTGG | bchG-F CGCCCGCTGTCATTCGAGATT  bchG-R GCGAGGATCGTGAGGACAAGG |
| nCBE-ubiA | TGCATAAGGTGCCCCCATGTCGG | ubiA-F CCCAGCGGCACGAAGAACCAC  ubiA-R GCGGATTCTCCCGTCGAAAGGTC |
| nCBE-ubiB | CCAGCGCGACTATCGCCGCGTGG | ubiB-F GTTCGCACCGCTGCCACCAT  ubiB-R GCAGCAGGTGAAGACCAACGAG |
| nCBE-ubiD | CGCATGAAACCTCACATGTCTGG | ubiD-F CAAAGCCGCAATAGACGGAATG  ubiD-R CGGAGCACGCCCGAGAAGGT |
| nCBE-ubiE1 | CGTGCAGGACGCGCTGAACGAGG | ubiE-F GCGGGATGGTCGTGGAAGAAC  ubiE-R GGAGGGAGACAGAAGCTCGATG |
| nCBE-ubiE2 | GGCCCATTGCATCATCGGGTTGG |  |
| nCBE-ubiE3 | CCAGAAGCTCCTCGATGTGGCGG |  |
| nCBE-ubiF | GCCAGGCGGTGCCCTCGTGCGGG | ubiF-F CGGCGCAAGCCCGAATGCCAC  ubiF-R CCCGAGGGTATTTGGGCAGAGTG |
| nCBE-ubiG | ATTCCATTGCAAACTCCTTGCGG | ubiG-F GATGTCCGAGACGAGGTGCCAG  ubiG-R ACCGATCAGTTCCCGCACAACC |
| nCBE-ubiH1 | CCAGCCGGCCGAGGCGAAGGCGG | ubiH-F GTCACCGACCTGCCGAACCAC  ubiH-R TCCGGGCGTTGTGGTGAGCT |
| nCBE-ubiH2 | GCTGCAGGTCATGCGCATCGTGG |  |
| nCBE-ubiX | TGCAGCTGAAGGAGCGGCGGCGG | ubiX-F CTACCGCATCGCCATCGTCTCG  ubiX-R GGCCAACGGCATCGTCATCG |
| d/nABE-appA0 | AAGGATGCAACACGACCTCGAGG | appA-F GTCTCGTCCATCCGTCCCTCCCTT  appA-R GCCCAGCCTGTCTGTTCTCGTCTC |
| d/nABE-ppsR0 | GAAGAGACATGCTGGCCGGCGGG | ppsR-F GCCATAATGATTTCCCTTGCC  ppsR-R GGAACATGAAGGTGGCGTGGAT |
| d/nABE-crtB0 | TCATTCCGCGGCAAGCCTTTCAGCAGG | crtB-F GGGCTGTTCGTCTGGTATTTCGG  crtB-R GAAGAAGACCGGCGTCCAGAGC |
| d/nABE-bchG0 | GGGCACGGAGCGGGGGTGTAAGG | bchG-F CGCCCGCTGTCATTCGAGATT  bchG-R GCGAGGATCGTGAGGACAAGG |
| All plasmids | Kana gene | Kana-cure-F ATCAGGTGCGACAATCTATC |
|  |  | Kana-cure-R AGCCGTTTCTGTAATGAAGGAG |
| All plasmids | Cas9 gene | Cas9-cure-F GGATGGTACTGAGGAATTATTG |
|  |  | Cas9-cure-R CAACCAGTATAACGGCGACG |
| All plasmids | rpoZ gene | rpoZ-cure-F ATGGCCCGCGTGACGGTTGAAG |
|  |  | rpoZ-cure-R TCAGTTCTGCCCCTGCGCTTC |

Table S3 IPTG-inducible CBEs in *Rhodobacter sphaeroides* 2.4.1

| dCBE-appA1 with IPTG induction in only phase 2 | | | | | |
| --- | --- | --- | --- | --- | --- |
| Plate replicate | Colony number | appA1 (0mM)  GCCACTGGAAGAAGACGCCCTGG | appA1 (0.05mM)  GCCACTGGAAGAAGACGCCCTGG | appA1 (0.2mM)  GCCACTGGAAGAAGACGCCCTGG | appA1 (0.5mM)  GCCACTGGAAGAAGACGCCCTGG |
| 1 | 1 | GCCACTGGAAGAAGACGCCC | G(T>C)(T>C)ACTGGAAGAAGACGCCC | GC(C>T)ACTGGAAGAAGACGCCC | G(C>T)(T>C)ACTGGAAGAAGACGCCC |
|  | 2 | GCCACTGGAAGAAGACGCCC | GC(T>C)ACTGGAAGAAGACGCCC | G(C>T)(T>C)ACTGGAAGAAGACGCCC | G(C>T)(T>C)ACTGGAAGAAGACGCCC |
|  | 3 | GCCACTGGAAGAAGACGCCC | GC(T=C)ACTGGAAGAAGACGCCC | G(C=T)(T>C)ACTGGAAGAAGACGCCC | G(C>T)(T>C)ACTGGAAGAAGACGCCC |
|  | 4 | GCCACTGGAAGAAGACGCCC | G(C>T)(T=C)ACTGGAAGAAGACGCCC | G(C>T)(T>C)ACTGGAAGAAGACGCCC | G(T>C)(T>C)ACTGGAAGAAGACGCCC |
|  | 5 | GCCACTGGAAGAAGACGCCC | GTTACTGGAAGAAGACGCCC | G(T>C)(T>C)ACTGGAAGAAGACGCCC | G(C>T)(T>C)ACTGGAAGAAGACGCCC |
|  | 6 | GCCACTGGAAGAAGACGCCC | GC(T=C)ACTGGAAGAAGACGCCC | G(C>T)(T>C)ACTGGAAGAAGACGCCC | G(C>T)TACTGGAAGAAGACGCCC |
|  | 7 | GCCACTGGAAGAAGACGCCC | G(T>C)(T>C)ACTGGAAGAAGACGCCC | G(C>T)(T>C)ACTGGAAGAAGACGCCC | G(C>T)(T>C)ACTGGAAGAAGACGCCC |
|  | 8 | GCCACTGGAAGAAGACGCCC | G(C>T)(T>C)ACTGGAAGAAGACGCCC | G(C>T)(T>C)ACTGGAAGAAGACGCCC | GTTACTGGAAGAAGACGCCC |
|  | 9 | GCCACTGGAAGAAGACGCCC | GCCACTGGAAGAAGACGCCC | G(C>T)(C>T)ACTGGAAGAAGACGCCC | G(C>T)(C>T)ACTGGAAGAAGACGCCC |
|  | 10 | GCCACTGGAAGAAGACGCCC | G(T>C)(T>C)ACTGGAAGAAGACGCCC | G(C=T)(T>C)ACTGGAAGAAGACGCCC | G(C=T)(C=T)ACTGGAAGAAGACGCCC |
| 2 | 11 | GCCACTGGAAGAAGACGCCC | G(T>C)(T>C)ACTGGAAGAAGACGCCC | G(T>C)TACTGGAAGAAGACGCCC | G(C>T)(T>C)ACTGGAAGAAGACGCCC |
|  | 12 | GCCACTGGAAGAAGACGCCC | GC(C>T)ACTGGAAGAAGACGCCC | G(C>T)(T=C)ACTGGAAGAAGACGCCC | G(T>C)(T>C)ACTGGAAGAAGACGCCC |
|  | 13 | GCCACTGGAAGAAGACGCCC | G(C>T)(T>C)ACTGGAAGAAGACGCCC | G(T=C)(T>C)ACTGGAAGAAGACGCCC | G(C>T)(T=C)ACTGGAAGAAGACGCCC |
|  | 14 | GCCACTGGAAGAAGACGCCC | G(T=C)(T>C)ACTGGAAGAAGACGCCC | G(T>C)TACTGGAAGAAGACGCCC | G(T=C)(T>C)ACTGGAAGAAGACGCCC |
|  | 15 | GCCACTGGAAGAAGACGCCC | G(C>T)(T=C)ACTGGAAGAAGACGCCC | G(T>C)(T>C)ACTGGAAGAAGACGCCC | G(T=C)(T>C)ACTGGAAGAAGACGCCC |
|  | 16 | GCCACTGGAAGAAGACGCCC | G(T=C)(T>C)ACTGGAAGAAGACGCCC | G(C>T)(T=C)ACTGGAAGAAGACGCCC | G(C>T)(T>C)ACTGGAAGAAGACGCCC |
|  | 17 | GCCACTGGAAGAAGACGCCC | G(T>C)TACTGGAAGAAGACGCCC | G(T=C)(T>C)ACTGGAAGAAGACGCCC | G(C>T)(T>C)ACTGGAAGAAGACGCCC |
|  | 18 | GCCACTGGAAGAAGACGCCC | G(T=C)(C>T)ACTGGAAGAAGACGCCC | G(T>C)(T>C)ACTGGAAGAAGACGCCC | G(T=C)(T>C)ACTGGAAGAAGACGCCC |
|  | 19 | G(T>C)(T=C)ACTGGAAGAAGACGCCC | G(C>T)(T>C)ACTGGAAGAAGACGCCC | GTTACTGGAAGAAGACGCCC | G(T>C)(T>C)ACTGGAAGAAGACGCCC |
|  | 20 | GCCACTGGAAGAAGACGCCC | G(T>C)(T>C)ACTGGAAGAAGACGCCC | G(T>C)(T>C)ACTGGAAGAAGACGCCC | G(T=C)(T>C)ACTGGAAGAAGACGCCC |
| dCBE-appA1 with IPTG induction in both phase 1 and phase 2 | | | | | |
| Plate replicate | Colony number | appA1 (0mM)  GCCACTGGAAGAAGACGCCCTGG | appA1 (0.05mM)  GCCACTGGAAGAAGACGCCCTGG | appA1 (0.2mM)  GCCACTGGAAGAAGACGCCCTGG | appA1 (0.5mM)  GCCACTGGAAGAAGACGCCCTGG |
| 1 | 1 | GC(C>T)ACTGGAAGAAGACGCCC | G(T>C)(T>C)ACTGGAAGAAGACGCCC | G(C>T)TACTGGAAGAAGACGCCC | G(C>T)(T>C)ACTGGAAGAAGACGCCC |
|  | 2 | GCCACTGGAAGAAGACGCCC | G(C>T)(T>C)ACTGGAAGAAGACGCCC | GC(C>T)ACTGGAAGAAGACGCCC | G(C>T)(T>C)ACTGGAAGAAGACGCCC |
|  | 3 | GC(C>T)ACTGGAAGAAGACGCCC | G(C>T)(T>C)ACTGGAAGAAGACGCCC | GTTACTGGAAGAAGACGCCC | G(C>T)(C>T)ACTGGAAGAAGACGCCC |
|  | 4 | GCCACTGGAAGAAGACGCCC | G(C>T)(T>C)ACTGGAAGAAGACGCCC | G(C>T)(T>C)ACTGGAAGAAGACGCCC | G(C>T)(T>C)ACTGGAAGAAGACGCCC |
|  | 5 | GCCACTGGAAGAAGACGCCC | G(C>T)(T>C)ACTGGAAGAAGACGCCC | G(T>C)(T>C)ACTGGAAGAAGACGCCC | G(C>T)TACTGGAAGAAGACGCCC |
|  | 6 | GCCACTGGAAGAAGACGCCC | G(C>T)(T>C)ACTGGAAGAAGACGCCC | G(C>T)(T>C)ACTGGAAGAAGACGCCC | G(C>T)(T>C)ACTGGAAGAAGACGCCC |
|  | 7 | GC(C>T)ACTGGAAGAAGACGCCC | GC(T>C)ACTGGAAGAAGACGCCC | G(T>C)(T>C)ACTGGAAGAAGACGCCC | GC(T>C)ACTGGAAGAAGACGCCC |
|  | 8 | GCCACTGGAAGAAGACGCCC | G(C>T)(T>C)ACTGGAAGAAGACGCCC | G(C>T)TACTGGAAGAAGACGCCC | G(C>T)(T>C)ACTGGAAGAAGACGCCC |
|  | 9 | GCCACTGGAAGAAGACGCCC | G(C>T)(T>C)ACTGGAAGAAGACGCCC | G(T>C)TACTGGAAGAAGACGCCC | GC(T>C)ACTGGAAGAAGACGCCC |
|  | 10 | GCCACTGGAAGAAGACGCCC | GCCACTGGAAGAAGACGCCC | G(T>C)(T>C)ACTGGAAGAAGACGCCC | G(C>T)(T>C)ACTGGAAGAAGACGCCC |
| 2 | 11 | G(C>T)(C>T)ACTGGAAGAAGACGCCC | G(T=C)(T>C)ACTGGAAGAAGACGCCC | G(C>T)(T=C)ACTGGAAGAAGACGCCC | G(T>C)(T>C)ACTGGAAGAAGACGCCC |
|  | 12 | GC(C>T)ACTGGAAGAAGACGCCC | G(T=C)TACTGGAAGAAGACGCCC | G(C>T)(T>C)ACTGGAAGAAGACGCCC | GTTACTGGAAGAAGACGCCC |
|  | 13 | GC(C>T)ACTGGAAGAAGACGCCC | G(C>T)(T>C)ACTGGAAGAAGACGCCC | G(T>C)(T>C)ACTGGAAGAAGACGCCC | G(C>T)(T=C)ACTGGAAGAAGACGCCC |
|  | 14 | G(T>C)(T>C)ACTGGAAGAAGACGCCC | G(C>T)(T>C)ACTGGAAGAAGACGCCC | G(T=C)(T>C)ACTGGAAGAAGACGCCC | G(T>C)(T>C)ACTGGAAGAAGACGCCC |
|  | 15 | G(T>C)(T=C)ACTGGAAGAAGACGCCC | G(C>T)(T=C)ACTGGAAGAAGACGCCC | G(T>C)(T>C)ACTGGAAGAAGACGCCC | G(C>T)(C=T)ACTGGAAGAAGACGCCC |
|  | 16 | GC(T=C)ACTGGAAGAAGACGCCC | G(C>T)(T=C)ACTGGAAGAAGACGCCC | G(T>C)(T>C)ACTGGAAGAAGACGCCC | G(C>T)(T>C)ACTGGAAGAAGACGCCC |
|  | 17 | GT(T>C)ACTGGAAGAAGACGCCC | GC>T)TACTGGAAGAAGACGCCC | G(C>T)(T>C)ACTGGAAGAAGACGCCC | G(T=C)(T>C)ACTGGAAGAAGACGCCC |
|  | 18 | GCCACTGGAAGAAGACGCCC | G(C>T)(T>C)ACTGGAAGAAGACGCCC | G(C>T)(T=C)ACTGGAAGAAGACGCCC | GTTATTGGAAGAAGACGCCC |
|  | 19 | GC(C>T)ACTGGAAGAAGACGCCC | G(C=T)(T>C)ACTGGAAGAAGACGCCC | G(T>C)(T>C)ACTGGAAGAAGACGCCC | G(T=C)(T>C)ACTGGAAGAAGACGCCC |
|  | 20 | GC(T=C)ACTGGAAGAAGACGCCC | G(C>T)(T>C)ACTGGAAGAAGACGCCC | GC(T=C)ACTGGAAGAAGACGCCC | G(C>T)(T>C)ACTGGAAGAAGACGCCC |
| nCBE-appA1 with IPTG induction in only phase 2 | | | | | |
| Plate replicate | Colony number | appA1 (0mM)  GCCACTGGAAGAAGACGCCCTGG | appA1 (0.05mM)  GCCACTGGAAGAAGACGCCCTGG | appA1 (0.2mM)  GCCACTGGAAGAAGACGCCCTGG | appA1 (0.5mM)  GCCACTGGAAGAAGACGCCCTGG |
| 1 | 1 | GC(T>C)ACTGGAAGAAGACGCCC | GTTA(T>C)TGGAAGAAGACGCCC | GTTATTGGAAGAAGACGCCC | GTTA(T>C)TGGAAGAAGACGCCC |
|  | 2 | GC(C>T)ACTGGAAGAAGACGCCC | GTTACTGGAAGAAGACGCCC | GTTA(C>T)TGGAAGAAGACGCCC | GTTATTGGAAGAAGACGCCC |
|  | 3 | GC(T>C)ACTGGAAGAAGACGCCC | GTTACTGGAAGAAGACGCCC | G(G>T=C)TACTGGAAGAAGACGCCC | GTTATTGGAAGAAGACGCCC |
|  | 4 | GCCACTGGAAGAAGACGCCC | GTTACTGGAAGAAGACGCCC | G(T>C)(T>C)ACTGGAAGAAGACGCCC | GTTATTGGAAGAAGACGCCC |
|  | 5 | GCTACTGGAAGAAGACGCCC | G(T>C)(T>C)ACTGGAAGAAGACGCCC | GTTA(C>T)TGGAAGAAGACGCCC | G(T>C)TACTGGAAGAAGACGCCC |
|  | 6 | GCCACTGGAAGAAGACGCCC | GTTACTGGAAGAAGACGCCC | GTTA(C>A)TGGAAGAAGACGCCC | GTTACTGGAAGAAGACGCCC |
|  | 7 | GCCACTGGAAGAAGACGCCC | GTTA(G>C)TGGAAGAAGACGCCC | GTTATTGGAAGAAGACGCCC | G(T>G)TACTGGAAGAAGACGCCC |
|  | 8 | GCCACTGGAAGAAGACGCCC | GT(T>C)ACTGGAAGAAGACGCCC | GTTATTGGAAGAAGACGCCC | GTTAATGGAAGAAGACGCCC |
|  | 9 | GC(C>T)ACTGGAAGAAGACGCCC | GGTA(A=C)TGGAAGAAGACGCCC | GGTACTGGAAGAAGACGCCC | GTTACTGGAAGAAGACGCCC |
|  | 10 | GCCACTGGAAGAAGACGCCC | GTTAATGGAAGAAGACGCCC | GTTA(C>A)TGGAAGAAGACGCCC | GTTA(C>A)TGGAAGAAGACGCCC |
| 2 | 11 | GCCACTGGAAGAAGACGCCC | GTTA(C>T)TGGAAGAAGACGCCC | GTTA(T>C)TGGAAGAAGACGCCC | GTTA(T>C)TGGAAGAAGACGCCC |
|  | 12 | GCCACTGGAAGAAGACGCCC | G(T>G)(T>G)ACTGGAAGAAGACGCCC | GTTA(T=C)TGGAAGAAGACGCCC | GT(T>G)A(T>C)TGGAAGAAGACGCCC |
|  | 13 | G(T>C)(T>C)ACTGGAAGAAGACGCCC | GTTACTGGAAGAAGACGCCC | G(T>C)TACTGGAAGAAGACGCCC | GTTACTGGAAGAAGACGCCC |
|  | 14 | GC(C>T)ACTGGAAGAAGACGCCC | GTTAATGGAAGAAGACGCCC | GTTACTGGAAGAAGACGCCC | GTTA(T>C)TGGAAGAAGACGCCC |
|  | 15 | GCCACTGGAAGAAGACGCCC | GT(T>G)ACTGGAAGAAGACGCCC | GTTACTGGAAGAAGACGCCC | GTTATTGGAAGAAGACGCCC |
|  | 16 | GC(T>C)ACTGGAAGAAGACGCCC | GT(T>G)ACTGGAAGAAGACGCCC | GTTA(C>T)TGGAAGAAGACGCCC | GTTACTGGAAGAAGACGCCC |
|  | 17 | GC(T=C)ACTGGAAGAAGACGCCC | GTTATTGGAAGAAGACGCCC | GTTACTGGAAGAAGACGCCC | GT(T>C)A(T>C)TGGAAGAAGACGCCC |
|  | 18 | GC(C>T)ACTGGAAGAAGACGCCC | GTTA(T=C)TGGAAGAAGACGCCC | GTTACTGGAAGAAGACGCCC | GTTA(A>C)TGGAAGAAGACGCCC |
|  | 19 | GC(C>T)ACTGGAAGAAGACGCCC | GTTACTGGAAGAAGACGCCC | GTTA(C>T)TGGAAGAAGACGCCC | GTTATTGGAAGAAGACGCCC |
|  | 20 | GCCACTGGAAGAAGACGCCC | GTTA(C>A)TGGAAGAAGACGCCC | GTTATTGGAAGAAGACGCCC | GTTATTGGAAGAAGACGCCC |
| 3 | 21 | GC(T>C)ACTGGAAGAAGACGCCC | GTTA(C>A)TGGAAGAAGACGCCC | GTTACTGGAAGAAGACGCCC | GTTATTGGAAGAAGACGCCC |
|  | 22 | G(T>C)(C>T)A(C>T)TGGAAGAAGACGCCC | GTTA(A>C=T)TGGAAGAAGACGCCC | GTTACTGGAAGAAGACGCCC | G(T>C)TACTGGAAGAAGACGCCC |
|  | 23 | GT(T>C)ACTGGAAGAAGACGCCC | GTGACTGGAAGAAGACGCCC | GTTATTGGAAGAAGACGCCC | GTTACTGGAAGAAGACGCCC |
|  | 24 | GCCACTGGAAGAAGACGCCC | GTTACTGGAAGAAGACGCCC | GTTACTGGAAGAAGACGCCC | G(T>C)TACTGGAAGAAGACGCCC |
|  | 25 | GC(C>T)ACTGGAAGAAGACGCCC | GTTA(C>T)TGGAAGAAGACGCCC | GTTACTGGAAGAAGACGCCC | G(G>C)(T>C)ACTGGAAGAAGACGCCC |
|  | 26 | G(C>T)(C>T)ACTGGAAGAAGACGCCC | GGTA(G=A>C) TGGAAGAAGACGCCC | GTTA(C>T)TGGAAGAAGACGCCC | GCTACTGGAAGAAGACGCCC |
|  | 27 | GCCACTGGAAGAAGACGCCC | GTTA(C=T)TGGAAGAAGACGCCC | GTTATTGGAAGAAGACGCCC | G(T>C)(T>C)ACTGGAAGAAGACGCCC |
|  | 28 | GC(C>T)ACTGGAAGAAGACGCCC | GTTACTGGAAGAAGACGCCC | GTTA(C>A)TGGAAGAAGACGCCC | GTTACTGGAAGAAGACGCCC |
|  | 29 | GCCACTGGAAGAAGACGCCC | GTTA(C>A)TGGAAGAAGACGCCC | GT(T=G)ACTGGAAGAAGACGCCC | GTTA(T>C)TGGAAGAAGACGCCC |
|  | 30 | GCCACTGGAAGAAGACGCCC | GTTA(C>T)TGGAAGAAGACGCCC | GT(G=T)ACTGGAAGAAGACGCCC | GTTATTGGAAGAAGACGCCC |
| nCBE-appA1 with IPTG induction in both phase 1 and phase 2 | | | | | |
| Plate replicate | Colony number | appA1 (0mM)  GCCACTGGAAGAAGACGCCCTGG | appA1 (0.05mM)  GCCACTGGAAGAAGACGCCCTGG | appA1 (0.2mM)  GCCACTGGAAGAAGACGCCCTGG | appA1 (0.5mM)  GCCACTGGAAGAAGACGCCCTGG |
| 1 | 1 | GCTACTGGAAGAAGACGCCC | GTTACTGGAAGAAGACGCCC | GTTACTGGAAGAAGACGCCC | GTTATTGGAAGAAGACGCCC |
|  | 2 | G(T>C)TACTGGAAGAAGACGCCC | GTTA(A>C)TGGAAGAAGACGCCC | GTTACTGGAAGAAGACGCCC | GTTACTGGAAGAAGACGCCC |
|  | 3 | GTTATTGGAAGAAGACGCCC | GTTATTGGAAGAAGACGCCC | GTTATTGGAAGAAGACGCCC | GTTA(A>C)TGGAAGAAGACGCCC |
|  | 4 | GTTACTGGAAGAAGACGCCC | GTTA(C>T)TGGAAGAAGACGCCC | GTTA(C>T)TGGAAGAAGACGCCC | GTTA(T=C)TGGAAGAAGACGCCC |
|  | 5 | GTTACTGGAAGAAGACGCCC | G(T>C)GACTGGAAGAAGACGCCC | GTTACTGGAAGAAGACGCCC | GTTATTGGAAGAAGACGCCC |
|  | 6 | G(C>T)(C>T)ACTGGAAGAAGACGCCC | G(C>T)(C>T)ACTGGAAGAAGACGCCC | GTTA(C>A)TGGAAGAAGACGCCC | GTTATTGGAAGAAGACGCCC |
|  | 7 | G(T>C)(C=G)ACTGGAAGAAGACGCCC | GTTACTGGAAGAAGACGCCC | GTTATTGGAAGAAGACGCCC | GTTACTGGAAGAAGACGCCC |
|  | 8 | GGGACTGGAAGAAGACGCCC | GTTATTGGAAGAAGACGCCC | GTTATTGGAAGAAGACGCCC | GTTATTGGAAGAAGACGCCC |
|  | 9 | GCTACTGGAAGAAGACGCCC | GTTATTGGAAGAAGACGCCC | GTTATTGGAAGAAGACGCCC | GTTATTGGAAGAAGACGCCC |
|  | 10 | GC(C>T)ACTGGAAGAAGACGCCC | GT(T>C)A(C>A)TGGAAGAAGACGCCC | GTTACTGGAAGAAGACGCCC | GTTA(C>A)TGGAAGAAGACGCCC |
| 2 | 11 | GTTACTGGAAGAAGACGCCC | GT(T>C)ACTGGAAGAAGACGCCC | G(T>C)(T>C)ACTGGAAGAAGACGCCC | GTTATTGGAAGAAGACGCCC |
|  | 12 | GC(C>T)ACTGGAAGAAGACGCCC | GTTA(C>T)TGGAAGAAGACGCCC | GTTACTGGAAGAAGACGCCC | GTTACTGGAAGAAGACGCCC |
|  | 13 | GCTACTGGAAGAAGACGCCC | GTTACTGGAAGAAGACGCCC | GCCACTGGAAGAAGACGCCC | GTTA(A>C)TGGAAGAAGACGCCC |
|  | 14 | G(C>T)(C>T)ACTGGAAGAAGACGCCC | GT(G>T)ACTGGAAGAAGACGCCC | GT(T>C)A(C>T)TGGAAGAAGACGCCC | GTTACTGGAAGAAGACGCCC |
|  | 15 | G(C>T)(C=G>T)ACTGGAAGAAGACGCCC | GT(G>T)ACTGGAAGAAGACGCCC | G(T>C)(T>C)ACTGGAAGAAGACGCCC | GTTACTGGAAGAAGACGCCC |
|  | 16 | GT(T>C)ACTGGAAGAAGACGCCC | GGTAATGGAAGAAGACGCCC | GTTA(C>A)TGGAAGAAGACGCCC | GTTACTGGAAGAAGACGCCC |
|  | 17 | G(C>T)(C>T)ACTGGAAGAAGACGCCC | GTGA(C>A)TGGAAGAAGACGCCC | GT(T>C)A(C>A)TGGAAGAAGACGCCC | G(T>C)(T>C)ACTGGAAGAAGACGCCC |
|  | 18 | GCTACTGGAAGAAGACGCCC | GTTA(C>T)TGGAAGAAGACGCCC | GTTACTGGAAGAAGACGCCC | GTTA(T>C)TGGAAGAAGACGCCC |
|  | 19 | GCTACTGGAAGAAGACGCCC | GTGA(G=C)TGGAAGAAGACGCCC | GCCACTGGAAGAAGACGCCC | GTTATTGGAAGAAGACGCCC |
|  | 20 | G(C>T)(C>T)ACTGGAAGAAGACGCCC | G(T>G)TA(C>T)TGGAAGAAGACGCCC | GT(T>G)ACTGGAAGAAGACGCCC | GTTACTGGAAGAAGACGCCC |
| 3 | 21 | G(C>T)(C>T)ACTGGAAGAAGACGCCC | GCCACTGGAAGAAGACGCCC | G(C>T)(C>T)ACTGGAAGAAGACGCCC | GTTATTGGAAGAAGACGCCC |
|  | 22 | GC(C>T)ACTGGAAGAAGACGCCC | GTTATTGGAAGAAGACGCCC | G(G>T)TA(C>T)TGGAAGAAGACGCCC | G(T>C)TACTGGAAGAAGACGCCC |
|  | 23 | GTTATTGGAAGAAGACGCCC | G(C>T)(C>T)ACTGGAAGAAGACGCCC | GTTACTGGAAGAAGACGCCC | GTTACTGGAAGAAGACGCCC |
|  | 24 | GC(T>C)ACTGGAAGAAGACGCCC | GTTA(C>T)TGGAAGAAGACGCCC | GTTAATGGAAGAAGACGCCC | G(T>C)TACTGGAAGAAGACGCCC |
|  | 25 | GC(C>T)ACTGGAAGAAGACGCCC | GTTATTGGAAGAAGACGCCC | GTTATTGGAAGAAGACGCCC | G(G>C)(T>C)ACTGGAAGAAGACGCCC |
|  | 26 | GTTACTGGAAGAAGACGCCC | GTTATTGGAAGAAGACGCCC | GTTATTGGAAGAAGACGCCC | GCTACTGGAAGAAGACGCCC |
|  | 27 | GC(T>C)ACTGGAAGAAGACGCCC | G(C>T)(C=T)ACTGGAAGAAGACGCCC | GTGA(C>T)TGGAAGAAGACGCCC | G(T>C)(T>C)ACTGGAAGAAGACGCCC |
|  | 28 | GTTACTGGAAGAAGACGCCC | GTTATTGGAAGAAGACGCCC | GTTA(C>A)TGGAAGAAGACGCCC | GTTACTGGAAGAAGACGCCC |
|  | 29 | G(C>T)(C>T)ACTGGAAGAAGACGCCC | GTTACTGGAAGAAGACGCCC | GTTATTGGAAGAAGACGCCC | GTTA(T>C)TGGAAGAAGACGCCC |
|  | 30 | G(C>T)(C>T)ACTGGAAGAAGACGCCC | GTTATTGGAAGAAGACGCCC | GTTATTGGAAGAAGACGCCC | GTTATTGGAAGAAGACGCCC |

Table S4 CBEs mediated single and double target editing in *Rhodobacter sphaeroides* 2.4.1

| The sequence data of dCBE series single target (After the first induction) | | | | | |
| --- | --- | --- | --- | --- | --- |
| Plate replicate | Colony number | appA1  GCCACTGGAAGAAGACGCCCTGG | appA2  CCATCCCGCAAAGCGGCGCTTGG | ppsR1  CGACTCACCACCGACTTCGCGGG | ppsR2  CCAGGTGGCCGAGATCTCTGCGG |
| 1 | 1 | GC(T>C)ACTGGAAGAAGACGCCC | (C>T)(T>C)AT(C>T>G)CCGCAAAGCGGCGCT | CGA(T>C)T(C>T)ACCACCGACTTCGC | C(C>T)AGGTGGCCGAGATCTCTG |
|  | 2 | G(C>T)(T>C)ACTGGAAGAAGACGCCC | C(C>T)ATCCCGCAAAGCGGCGCT | CGA(C>T)TCACCACCGACTTCGC | C(C>T)AGGTGGCCGAGATCTCTG |
|  | 3 | G(C>T)(T>C)ACTGGAAGAAGACGCCC | C(T=C)AT(C>T)CCGCAAAGCGGCGCT | CGACTCACCACCGACTTCGC | C(C>T)AGGTGGCCGAGATCTCTG |
|  | 4 | G(C>T)TACTGGAAGAAGACGCCC | C(T>G>C)AT(T>C)CCGCAAAGCGGCGCT | CGA(C>T)TCACCACCGACTTCGC | C(C>T)AGGTGGCCGAGATCTCTG |
|  | 5 | G(C>T)(T>C)ACTGGAAGAAGACGCCC | C(T>C)AT(T>C>G)CCGCAAAGCGGCGCT | CGA(C>T)TCACCACCGACTTCGC | C(C>T)AGGTGGCCGAGATCTCTG |
|  | 6 | GC(T>C)ACTGGAAGAAGACGCCC | (C>T)(T>C)AT(C>T)CCGCAAAGCGGCGCT | CGA(C>T)T(C>T)ACCACCGACTTCGC | C(C>T)AGGTGGCCGAGATCTCTG |
|  | 7 | GC(T>C)ACTGGAAGAAGACGCCC | C(C>T)ATCCCGCAAAGCGGCGCT | CGA(C>T)TCACCACCGACTTCGC | (C>T)(C>T)AGGTGGCCGAGATCTCTG |
|  | 8 | GC(C>T)ACTGGAAGAAGACGCCC | (C>T)TAT(T>C)(T>C)CGCAAAGCGGCGCT | CGA(C=T)TCACCACCGACTTCGC | C(C>T)AGGTGGCCGAGATCTCTG |
|  | 9 | GCTACTGGAAGAAGACGCCC | C(T=C)ATCCCGCAAAGCGGCGCT | (T>C)GA(T>C)TCACCACCGACTTCGC | CCAGGTGGCCGAGATCTCTG |
|  | 10 | GC(T>C)ACTGGAAGAAGACGCCC | C(T>C)AT(C>T)CCGCAAAGCGGCGCT | CGA(C>T)TCACCACCGACTTCGC | (C>T)(C>T)AGGTGGCCGAGATCTCTG |
| 2 | 11 | G(C=T)(T>C)ACTGGAAGAAGACGCCC | C(T>C)AT(C>T)CCGCAAAGCGGCGCT | CGA(C=T)TCACCACCGACTTCGC | (C>T)(C>T)AGGTGGCCGAGATCTCTG |
|  | 12 | GC(T>C)ACTGGAAGAAGACGCCC | CTAT(C>T)CCGCAAAGCGGCGCT | CGA(C>T)TCACCACCGACTTCGC | CCAGGTGGCCGAGATCTCTG |
|  | 13 | GC(T>C)ACTGGAAGAAGACGCCC | C(T=C)AT(C>T)CCGCAAAGCGGCGCT | CGA(C>T)TCACCACCGACTTCGC | (C>T)(C>T)AGGTGGCCGAGATCTCTG |
|  | 14 | GC(T>C)ACTGGAAGAAGACGCCC | C(T>C)AT(C>T)CCGCAAAGCGGCGCT | CGA(C>T)TCACCACCGACTTCGC | C(C>T)AGGTGGCCGAGATCTCTG |
|  | 15 | GC(T>C)ACTGGAAGAAGACGCCC | (T>C)(T>C)AT(T>C)CCGCAAAGCGGCGCT | CGA(C>T)TCACCACCGACTTCGC | (C>T)(C>T)AGGTGGCCGAGATCTCTG |
|  | 16 | G(C>T)(T>C)ACTGGAAGAAGACGCCC | C(T>C)AT(C>T)CCGCAAAGCGGCGCT | CGA(C=T)TCACCACCGACTTCGC | C(C>T)AGGTGGCCGAGATCTCTG |
|  | 17 | GC(T>C)ACTGGAAGAAGACGCCC | (G>T)TAT(T>C)CCGCAAAGCGGCGCT | CGA(C>T)TCACCACCGACTTCGC | C(C>T)AGGTGGCCGAGATCTCTG |
|  | 18 | GC(C>T)ACTGGAAGAAGACGCCC | C(T>C)AT(T=C)CCGCAAAGCGGCGCT | CGA(C>T)TCACCACCGACTTCGC | C(C>T)AGGTGGCCGAGATCTCTG |
|  | 19 | GCTACTGGAAGAAGACGCCC | CCATCCCGCAAAGCGGCGCT | CGA(C>T)TCACCACCGACTTCGC | (C>T)(C>T)AGGTGGCCGAGATCTCTG |
|  | 20 | GC(T>C)ACTGGAAGAAGACGCCC | CTAT(C>T)CCGCAAAGCGGCGCT | CGA(C=T)TCACCACCGACTTCGC | C(T=C)AGGTGGCCGAGATCTCTG |
| 3 | 21 | G(T>C)(T>C)ACTGGAAGAAGACGCCC | (C>T)(T=C)AT(C>T)CCGCAAAGCGGCGCT | CGA(C=T)TCACCACCGACTTCGC | C(C>T)AGGTGGCCGAGATCTCTG |
|  | 22 | G(T>C)TACTGGAAGAAGACGCCC | C(T>C)AT(C>T)CCGCAAAGCGGCGCT | CGA(C=T)T(C>T)ACCACCGACTTCGC | C(C>T)AGGTGGCCGAGATCTCTG |
|  | 23 | GCTACTGGAAGAAGACGCCC | C(C>T)ATCCCGCAAAGCGGCGCT | CGA(C>T)TCACCACCGACTTCGC | C(C>T)AGGTGGCCGAGATCTCTG |
|  | 24 | G(C>T)(T>C)ACTGGAAGAAGACGCCC | C(T>C)AT(C>T)CCGCAAAGCGGCGCT | CGA(C>T)TCACCACCGACTTCGC | C(C>T)AGGTGGCCGAGATCTCTG |
|  | 25 | GC(T>C)ACTGGAAGAAGACGCCC | CCATCCCGCAAAGCGGCGCT | CGA(C>T)TCACCACCGACTTCGC | C(C>T)AGGTGGCCGAGATCTCTG |
|  | 26 | G(C>T)(T>C)ACTGGAAGAAGACGCCC | C(T>C)AT(C>T)CCGCAAAGCGGCGCT | CGA(C>T)T(C>T)ACCACCGACTTCGC | C(C>T)AGGTGGCCGAGATCTCTG |
|  | 27 | G(C>T)(T>C)ACTGGAAGAAGACGCCC | C(T>C)AT(C>T)CCGCAAAGCGGCGCT | CGA(C=T)T(C>T)ACCACCGACTTCGC | C(C>T)AGGTGGCCGAGATCTCTG |
|  | 28 | G(T>C)(T>C)ACTGGAAGAAGACGCCC | C(T=C)ATCCCGCAAAGCGGCGCT | CGA(C=T)TCACCACCGACTTCGC | C(C>T)AGGTGGCCGAGATCTCTG |
|  | 29 | G(C>T)(T>C)ACTGGAAGAAGACGCCC | C(C=T)AT(C>T)CCGCAAAGCGGCGCT | CGA(C>T)TCACCACCGACTTCGC | C(C>T)AGGTGGCCGAGATCTCTG |
|  | 30 | GC(T>C)ACTGGAAGAAGACGCCC | CTATTCCGCAAAGCGGCGCT | CGA(C>T)T(C>T)ACCACCGACTTCGC | (C>T)(C>T)AGGTGGCCGAGATCTCTG |
| The sequence data of dCBE series double target (After the first induction) | | | | | |
| Plate replicate | Colony number | appA1-appA2 | | appA1-ppsR2 | |
|  |  | appA1  GCCACTGGAAGAAGACGCCCTGG | appA2  CCATCCCGCAAAGCGGCGCTTGG | appA1  GCCACTGGAAGAAGACGCCCTGG | ppsR2  CCAGGTGGCCGAGATCTCTGCGG |
| 1 | 1 | GC(C>T)ACTGGAAGAAGACGCCC | C(T>C)AT(C>T)CCGCAAAGCGGCGCT | GC(C>T)ACTGGAAGAAGACGCCC | C(C>T)AGGTGGCCGAGATCTCTG |
|  | 2 | GC(T>C)ACTGGAAGAAGACGCCC | C(T>C)AT(C>T)CCGCAAAGCGGCGCT | GC(C>T)ACTGGAAGAAGACGCCC | C(C>T)AGGTGGCCGAGATCTCTG |
|  | 3 | GC(C>T)ACTGGAAGAAGACGCCC | C(T>C)AT(C>T)CCGCAAAGCGGCGCT | GC(C=T)ACTGGAAGAAGACGCCC | C(C>T)AGGTGGCCGAGATCTCTG |
|  | 4 | GC(C>T)ACTGGAAGAAGACGCCC | C(T>C)AT(C>T)CCGCAAAGCGGCGCT | GC(C>T)ACTGGAAGAAGACGCCC | (C>T)(C>T)AGGTGGCCGAGATCTCTG |
|  | 5 | G(T>C)(T>C)ACTGGAAGAAGACGCCC | (C>T)(T>C)AT(T>C)CCGCAAAGCGGCGCT | GC(C>T)ACTGGAAGAAGACGCCC | CCAGGTGGCCGAGATCTCTG |
|  | 6 | GC(C>T)ACTGGAAGAAGACGCCC | C(T>C)AT(C>T)CCGCAAAGCGGCGCT | GC(C>T)ACTGGAAGAAGACGCCC | C(C>T)AGGTGGCCGAGATCTCTG |
|  | 7 | GC(C>T)ACTGGAAGAAGACGCCC | C(T>C)AT(C>T)CCGCAAAGCGGCGCT | G(C>T)(C>T)ACTGGAAGAAGACGCCC | (C>T)(C>T)AGGTGGCCGAGATCTCTG |
|  | 8 | GC(C>T)ACTGGAAGAAGACGCCC | C(T=C)AT(C>T)CCGCAAAGCGGCGCT | GC(C>T)ACTGGAAGAAGACGCCC | C(C>T)AGGTGGCCGAGATCTCTG |
|  | 9 | GC(C>T)ACTGGAAGAAGACGCCC | (C>T)(C>T)AT(C>T)CCGCAAAGCGGCGCT | GC(C>T)ACTGGAAGAAGACGCCC | C(C>T)AGGTGGCCGAGATCTCTG |
|  | 10 | GC(C>T)ACTGGAAGAAGACGCCC | C(T>C)ATCCCGCAAAGCGGCGCT | GC(C>T)ACTGGAAGAAGACGCCC | (C>T)(C>T)AGGTGGCCGAGATCTCTG |
| 2 | 11 | G(C>T)(T>C)ACTGGAAGAAGACGCCC | CCATCCCGCAAAGCGGCGCT | GC(C>T)ACTGGAAGAAGACGCCC | C(C>T)AGGTGGCCGAGATCTCTG |
|  | 12 | GC(T>C)ACTGGAAGAAGACGCCC | C(T>C)AT(C>T)CCGCAAAGCGGCGCT | GC(C>T)ACTGGAAGAAGACGCCC | C(C>T)AGGTGGCCGAGATCTCTG |
|  | 13 | GC(T=C)ACTGGAAGAAGACGCCC | C(T>C)AT(C>T)CCGCAAAGCGGCGCT | GC(C>T)ACTGGAAGAAGACGCCC | C(C>T)AGGTGGCCGAGATCTCTG |
|  | 14 | G(C>T)(C>T)ACTGGAAGAAGACGCCC | C(C>T)AT(C>T)CCGCAAAGCGGCGCT | GC(C>T)ACTGGAAGAAGACGCCC | (C>T)(C>T)AGGTGGCCGAGATCTCTG |
|  | 15 | GC(T>C)ACTGGAAGAAGACGCCC | C(T>C)AT(C>T)CCGCAAAGCGGCGCT | G(C>T)(C=T)ACTGGAAGAAGACGCCC | C(C>T)AGGTGGCCGAGATCTCTG |
|  | 16 | GC(C>T)ACTGGAAGAAGACGCCC | C(T>C)ATCCCGCAAAGCGGCGCT | G(C>T)(T>C)ACTGGAAGAAGACGCCC | C(C>T)AGGTGGCCGAGATCTCTG |
|  | 17 | GC(C>T)ACTGGAAGAAGACGCCC | C(T>C)AT(C>T)CCGCAAAGCGGCGCT | GC(C>T)ACTGGAAGAAGACGCCC | C(C>T)AGGTGGCCGAGATCTCTG |
|  | 18 | GC(C>T)ACTGGAAGAAGACGCCC | C(T>C)AT(C>T)CCGCAAAGCGGCGCT | GC(C>T)ACTGGAAGAAGACGCCC | C(C>T)AGGTGGCCGAGATCTCTG |
|  | 19 | GC(C>T)ACTGGAAGAAGACGCCC | C(T=C)ATCCCGCAAAGCGGCGCT | G(C>T)(T>C)ACTGGAAGAAGACGCCC | C(C>T)AGGTGGCCGAGATCTCTG |
|  | 20 | G(C>T)(C>T)ACTGGAAGAAGACGCCC | C(T>C)AT(C>T)CCGCAAAGCGGCGCT | GC(C>T)ACTGGAAGAAGACGCCC | C(C>T)AGGTGGCCGAGATCTCTG |
| 3 | 21 | GC(T>C)ACTGGAAGAAGACGCCC | C(T>C)AT(C>T)CCGCAAAGCGGCGCT | GC(C>T)ACTGGAAGAAGACGCCC | C(C>T)AGGTGGCCGAGATCTCTG |
|  | 22 | GC(C>T)ACTGGAAGAAGACGCCC | C(T>C)AT(C>T)CCGCAAAGCGGCGCT | G(C>T)(T=C)ACTGGAAGAAGACGCCC | C(C>T)AGGTGGCCGAGATCTCTG |
|  | 23 | GC(C>T)ACTGGAAGAAGACGCCC | C(T=C)AT(C>T)CCGCAAAGCGGCGCT | GC(C>T)ACTGGAAGAAGACGCCC | (C>T)(C>T)AGGTGGCCGAGATCTCTG |
|  | 24 | GC(C>T)ACTGGAAGAAGACGCCC | C(T>C)ATCCCGCAAAGCGGCGCT | GC(T>C)ACTGGAAGAAGACGCCC | C(C>T)AGGTGGCCGAGATCTCTG |
|  | 25 | G(C>T)(C>T)ACTGGAAGAAGACGCCC | C(T=C)ATCCCGCAAAGCGGCGCT | GC(C>T)ACTGGAAGAAGACGCCC | (C>T)(C>T)AGGTGGCCGAGATCTCTG |
|  | 26 | GC(C>T)ACTGGAAGAAGACGCCC | CTATCCCGCAAAGCGGCGCT | GC(C>T)ACTGGAAGAAGACGCCC | C(C>T)AGGTGGCCGAGATCTCTG |
|  | 27 | GC(T>C)ACTGGAAGAAGACGCCC | CCATCCCGCAAAGCGGCGCT | GC(C>T)ACTGGAAGAAGACGCCC | C(C>T)AGGTGGCCGAGATCTCTG |
|  | 28 | GC(C>T)ACTGGAAGAAGACGCCC | C(T>C)AT(C>T)CCGCAAAGCGGCGCT | GC(C>T)ACTGGAAGAAGACGCCC | (C>T)(C>T)AGGTGGCCGAGATCTCTG |
|  | 29 | GC(C>T)ACTGGAAGAAGACGCCC | C(T>C)AT(C>T)CCGCAAAGCGGCGCT | GC(C>T)ACTGGAAGAAGACGCCC | C(C>T)AGGTGGCCGAGATCTCTG |
|  | 30 | GC(C>T)ACTGGAAGAAGACGCCC | C(T>C)AT(C>T)CCGCAAAGCGGCGCT | GC(T>C)ACTGGAAGAAGACGCCC | C(C>T)AGGTGGCCGAGATCTCTG |
| The sequence data of nCBE series single target (After the first induction) | | | | | |
| Plate replicate | Colony number | appA1  GCCACTGGAAGAAGACGCCCTGG | appA2  CCATCCCGCAAAGCGGCGCTTGG | ppsR1  CGACTCACCACCGACTTCGCGGG | ppsR2  CCAGGTGGCCGAGATCTCTGCGG |
| 1 | 1 | GTTATTGGAAGAAGACGCCC | (C>T)TATT(C>T)CGCAAAGCGGCGCT | (C>T)GATT(C>T)ACCACCGACTTCGC | C(T>C)AGGTGGCCGAGATCTCTG |
|  | 2 | GTTACTGGAAGAAGACGCCC | CTATGCCGCAAAGCGGCGCT | CGATTTACCACCGACTTCGC | C(T>C)AGGTGGCCGAGATCTCTG |
|  | 3 | GTTA(A>C)TGGAAGAAGACGCCC | TTATTCCGCAAAGCGGCGCT | CGATT(C>T)ACCACCGACTTCGC | C(T=C)AGGTGGCCGAGATCTCTG |
|  | 4 | GTTACTGGAAGAAGACGCCC | CTATGCCGCAAAGCGGCGCT | CGATT(T>C)ACCACCGACTTCGC | C(T=C)AGGTGGCCGAGATCTCTG |
|  | 5 | GTTACTGGAAGAAGACGCCC | CTATTCCGCAAAGCGGCGCT | CGATT(C>T)ACCACCGACTTCGC | C(C>T)AGGTGGCCGAGATCTCTG |
|  | 6 | GTTACTGGAAGAAGACGCCC | CTATGCCGCAAAGCGGCGCT | CGATT(C>T)ACCACCGACTTCGC | C(T>C)AGGTGGCCGAGATCTCTG |
|  | 7 | G(T>C)(T>C)ACTGGAAGAAGACGCCC | (T>C)TATT(T>C)CGCAAAGCGGCGCT | CGA(T>G)TTACCACCGACTTCGC | C(C>T)AGGTGGCCGAGATCTCTG |
|  | 8 | GTTA(T>C)TGGAAGAAGACGCCC | CTATT(C>T)TGCAAAGCGGCGCT | CGACTCACCACCGACTTCGC | C(T>C)AGGTGGCCGAGATCTCTG |
|  | 9 | GTTATTGGAAGAAGACGCCC | C(T>G)AT(T>G)CCGCAAAGCGGCGCT | CGATT(C=T)ACCACCGACTTCGC | C(T>C)AGGTGGCCGAGATCTCTG |
|  | 10 | GTTACTGGAAGAAGACGCCC | CTATAC(T>C)GCAAAGCGGCGCT | TGATT(T>C)ACCACCGACTTCGC | C(T>C)AGGTGGCCGAGATCTCTG |
| 2 | 11 | GTTATTGGAAGAAGACGCCCTGG | C(T>G)ATT(C>T)CGCAAAGCGGCGCT | CGATTTA(T>C)CACCGACTTCGC | C(T>C)AGGTGGCCGAGATCTCTG |
|  | 12 | GTTACTGGAAGAAGACGCCCTGG | TTATA(C>T)(C>G)GCAAAGCGGCGCT | TGATTTA(C>T)CA(C=T)CGACTTCGC | (C>T)(T>C)AGGTGGCCGAGATCTCTG |
|  | 13 | GTTA(A>C)TGGAAGAAGACGCCCTGG | CTATGCCGCAAAGCGGCGCT | CGATT(C>T)AC(C>G)ACCGACTTCGC | (C>T)(T>C)AGGTGGCCGAGATCTCTG |
|  | 14 | GTTA(T=C)TGGAAGAAGACGCCCTGG | TTATTCCGCAAAGCGGCGCT | CGAATCACCACCGACTTCGC | (C>T)TAGGTGGCCGAGATCTCTG |
|  | 15 | GTTATTGGAAGAAGACGCCCTGG | (T>C)TATTCCGCAAAGCGGCGCT | CGACTCACCACCGACTTCGC | (C>T)TAGGTGGCCGAGATCTCTG |
|  | 16 | GTTATTGGAAGAAGACGCCCTGG | CTATTCCGCAAAGCGGCGCT | CGATT(T>C)ACCACCGACTTCGC | C(T>C)AGGTGGCCGAGATCTCTG |
|  | 17 | GTTACTGGAAGAAGACGCCCTGG | TTAT(G=T)CCGCAAAGCGGCGCT | CGA(G>T)T(G>T)AC(C>T)ACCGACTTCGC | (C>T)(T>C)AGGTGGCCGAGATCTCTG |
|  | 18 | GTTATTGGAAGAAGACGCCCTGG | CTATGCCGCAAAGCGGCGCT | (C>G)GATT(C>T)ACCACCGACTTCGC | (C>T)TAGGTGGCCGAGATCTCTG |
|  | 19 | GTTATTGGAAGAAGACGCCCTGG | CTATTT(T>C)GCAAAGCGGCGCT | CGATT(C>T)ACCACCGACTTCGC | (C>T)(T>C)AGGTGGCCGAGATCTCTG |
|  | 20 | GTTA(C>A)TGGAAGAAGACGCCCTGG | (T>C)TATT(C>G)CGCAAAGCGGCGCT | CGATT(C>T)ACCACCGACTTCGC | (C>T)(T>C)AGGTGGCCGAGATCTCTG |
| 3 | 21 | GTTATTGGAAGAAGACGCCC | CTATTCCGCAAAGCGGCGCT | CGAGT(C>G)ACCACCGACTTCGC | C(T=C)AGGTGGCCGAGATCTCTG |
|  | 22 | G(T>C)TACTGGAAGAAGACGCCC | CTATT(C>T)(T>C)GCAAAGCGGCGCT | CGA(G>T)T(C=T)ACCACCGACTTCGC | C(T>C)AGGTGGCCGAGATCTCTG |
|  | 23 | GTTACTGGAAGAAGACGCCC | CTATTCCGCAAAGCGGCGCT | TGATT(C>T)ACCACCGACTTCGC | C(T=C)AGGTGGCCGAGATCTCTG |
|  | 24 | G(T>C)TACTGGAAGAAGACGCCC | CTATTTTGCAAAGCGGCGCT | (C>T)GATT(C>T)ACCACCGACTTCGC | C(T>C)AGGTGGCCGAGATCTCTG |
|  | 25 | G(G>C)(T>C)ACTGGAAGAAGACGCCC | TTATGCCGCAAAGCGGCGCT | CGATT(T>C)ACCACCGACTTCGC | C(C>T)AGGTGGCCGAGATCTCTG |
|  | 26 | GCTACTGGAAGAAGACGCCC | CTATT(C>T)CGCAAAGCGGCGCT | CGA(G>T)T(C>T)AC(C>G)ACCGACTTCGC | C(C>T)AGGTGGCCGAGATCTCTG |
|  | 27 | G(T>C)(T>C)ACTGGAAGAAGACGCCC | (C>T)TATTCCGCAAAGCGGCGCT | CGATT(T>C)ACCACCGACTTCGC | C(T>C)AGGTGGCCGAGATCTCTG |
|  | 28 | GTTACTGGAAGAAGACGCCC | CTATGCCGCAAAGCGGCGCT | CGA(G>T)T(C>T)ACCACCGACTTCGC | C(T=C)AGGTGGCCGAGATCTCTG |
|  | 29 | GTTA(T>C)TGGAAGAAGACGCCC | TTATT(T>C)CGCAAAGCGGCGCT | CGATT(C>T)ACCACCGACTTCGC | C(T=C)AGGTGGCCGAGATCTCTG |
|  | 30 | GTTATTGGAAGAAGACGCCC | (C>T)TAT(T>G)(C>T)CGCAAAGCGGCGCT | CGATT(T>C)ACCACCGACTTCGC | C(T>C)AGGTGGCCGAGATCTCTG |
| The sequence data of nCBE series single target (After the first induction) | | | | | |
| Plate replicate | Colony number | crtB2  GCCAGAAGCTCGTCTGGCTCGGG | bchG1  TGAGCCAGGCCGCGAACAACTGG |  |  |
| 1 | 1 | GCTAGAAGCTCGTCTGGCTCGGG | TGAGT(C>T)AGGCCGCGAACAACTGG |  |  |
|  | 2 | GCTAGAAGCTCGTCTGGCTCGGG | TGAG(C>G)TAGGCCGCGAACAACTGG |  |  |
|  | 3 | GC(T>C)AGAAGCTCGTCTGGCTCGGG | TGAGGCAGGCCGCGAACAACTGG |  |  |
|  | 4 | GT(T>C)AGAAGCTCGTCTGGCTCGGG | TGAGT(T>C)AGGCCGCGAACAACTGG |  |  |
|  | 5 | G(C>T)(T>C)AGAAGCTCGTCTGGCTCGGG | TGAG(C=T)CAGGCCGCGAACAACTGG |  |  |
|  | 6 | GTGAGAAGCTCGTCTGGCTCGGG | TGAGTCAGGCCGTGAACAACTGG |  |  |
|  | 7 | G(T>C)TAGAAGCTCGTCTGGCTCGGG | TGAGTTAGGCCGCGAACAACTGG |  |  |
|  | 8 | GTTAGAAGCTCGTCTGGCTCGGG | TGAG(C>T)CAGGCCGCGAACAACTGG |  |  |
|  | 9 | GCTAGAAGCTCGTCTGGCTCGGG | TGAGT(G>C)AGGCCGCGAACAACTGG |  |  |
|  | 10 | GCTAGAAGCTCGTCTGGCTCGGG | TGAG(C=T)CAGGCCGCGAACAACTGG |  |  |
| 2 | 11 | G(C>G)TAGAAGCTCGTCTGGCTCGGG | TGAGG(T>C)AGGCCGCGAACAACTGG |  |  |
|  | 12 | G(G>C)TAGAAGCTCGTCTGGCTCGGG | TGAGGCAGGCCGCGAACAACTGG |  |  |
|  | 13 | G(C>G)(T>C)AGAAGCTCGTCTGGCTCGGG | TGAGT(T>C)AGGCCGCGAACAACTGG |  |  |
|  | 14 | GCTAGAAGCTCGTCTGGCTCGGG | TGAGGTAGGCCGCGAACAACTGG |  |  |
|  | 15 | GC(T>C)AGAAGCTCGTCTGGCTCGGG | TGAG(C>T)CAGGCCGCGAACAACTGG |  |  |
|  | 16 | G(C>T)(T>G)AGAAGCTCGTCTGGCTCGGG | TGAGTTAGGCCGCGAACAACTGG |  |  |
|  | 17 | G(C>T)TAGAAGCTCGTCTGGCTCGGG | TGAGTTAGGCCGCGAACAACTGG |  |  |
|  | 18 | G(C>T)(T>G)AGAAGCTCGTCTGGCTCGGG | TGAGTCAGGCCGCGAACAACTGG |  |  |
|  | 19 | GT(T>C)AGAAGCTCGTCTGGCTCGGG | TGAGTCAGGCCGCGAACAACTGG |  |  |
|  | 20 | GCTAGAAGCTCGTCTGGCTCGGG | TGAG(T>G)(C>T)AGGCCGCGAACAACTGG |  |  |
| 3 | 21 |  | TGAG(T>C)(C>T)AGGCCGCGAACAACTGG |  |  |
|  | 22 |  | TGAG(C=T)CAGGCCGCGAACAACTGG |  |  |
|  | 23 |  | TGAG(G>T)(T>C)AGGCCGCGAACAACTGG |  |  |
|  | 24 |  | TGAG(T>C)CAGGCCGCGAACAACTGG |  |  |
|  | 25 |  | TGAGTTAGGCCGCGAACAACTGG |  |  |
|  | 26 |  | TGAGT(T>C)AGGCCGCGAACAACTGG |  |  |
|  | 27 |  | TGAGCCAGGCCGCGAACAACTGG |  |  |
|  | 28 |  | TGAGTTAGGCCGCGAACAACTGG |  |  |
|  | 29 |  | TGAGT(T>C)AGGCCGCGAACAACTGG |  |  |
|  | 30 |  | TGAGTCAGGCTGCGAACAACTGG |  |  |
| The sequence data of nCBE series double target (After the first induction) | | | | | |
| Plate replicate | Colony number | appA1-appA2 | | appA1-ppsR2 | |
|  |  | appA1  GCCACTGGAAGAAGACGCCCTGG | appA2  CCATCCCGCAAAGCGGCGCTTGG | appA1  GCCACTGGAAGAAGACGCCCTGG | ppsR2  CCAGGTGGCCGAGATCTCTGCGG |
| 1 | 1 | GCTACTGGAAGAAGACGCCC | CTATGCCGCAAAGCGGCGCT | GTTACTGGAAGAAGACGCCC | C(T>C)AGGTGGCCGAGATCTCTG |
|  | 2 | G(T>C)(T>C)ACTGGAAGAAGACGCCC | CTATTCCGCAAAGCGGCGCT | G(C>T)TACTGGAAGAAGACGCCC | C(C>T)AGGTGGCCGAGATCTCTG |
|  | 3 | G(T>C)(T>C)A(C>T)TGGAAGAAGACGCCC | CTATT(C>T)(C>T)GCAAAGCGGCGCT | G(C>T)TACTGGAAGAAGACGCCC | C(T>C)AGGTGGCCGAGATCTCTG |
|  | 4 | G(T>C)TACTGGAAGAAGACGCCC | TTATGCCGCAAAGCGGCGCT | G(T=C)(T>C)ACTGGAAGAAGACGCCC | C(T>C)AGGTGGCCGAGATCTCTG |
|  | 5 | G(C>T)(T>C)ACTGGAAGAAGACGCCC | TTATTCCGCAAAGCGGCGCT | G(C>T)TACTGGAAGAAGACGCCC | C(T=C)AGGTGGCCGAGATCTCTG |
|  | 6 | G(C>T)(T>C)ACTGGAAGAAGACGCCC | TTATGCCGCAAAGCGGCGCT | G(T>C)TACTGGAAGAAGACGCCC | C(T>C)AGGTGGCCGAGATCTCTG |
|  | 7 | GCTACTGGAAGAAGACGCCC | CTATGCCGCAAAGCGGCGCT | G(C>T)(T>C)ACTGGAAGAAGACGCCC | C(T>C)AGGTGGCCGAGATCTCTG |
|  | 8 | G(C>T)(T>C)ACTGGAAGAAGACGCCC | (T>C)TATGCCGCAAAGCGGCGCT | G(T>C)TACTGGAAGAAGACGCCC | C(C>T)AGGTGGCCGAGATCTCTG |
|  | 9 | GC(T>C)ACTGGAAGAAGACGCCC | (C>T)(T>G)AT(T>G)CCGCAAAGCGGCGCT | G(C>T)TACTGGAAGAAGACGCCC | C(T=C)AGGTGGCCGAGATCTCTG |
|  | 10 | GTTACTGGAAGAAGACGCCC | CGATTCCGCAAAGCGGCGCT | G(C>T)TACTGGAAGAAGACGCCC | C(T>C)AGGTGGCCGAGATCTCTG |
| 2 | 11 | GC(T>C)ACTGGAAGAAGACGCCC | CTAT(T>G)CCGCAAAGCGGCGCT | G(C>T)TACTGGAAGAAGACGCCC | C(T>C)AGGTGGCCGAGATCTCTG |
|  | 12 | G(T>C)(T>C)ACTGGAAGAAGACGCCC | (T>C)TAT(G=T)CCGCAAAGCGGCGCT | G(T>C)(T>C)ACTGGAAGAAGACGCCC | C(T>C)AGGTGGCCGAGATCTCTG |
|  | 13 | GTTAATGGAAGAAGACGCCC | CTATGCCGCAAAGCGGCGCT | G(T>C)(T>C)ACTGGAAGAAGACGCCC | C(T>C)AGGTGGCCGAGATCTCTG |
|  | 14 | GC(C>T)ACTGGAAGAAGACGCCC | CTATTCCGCAAAGCGGCGCT | GTTACTGGAAGAAGACGCCC | C(T>C)AGGTGGCCGAGATCTCTG |
|  | 15 | GTTACTGGAAGAAGACGCCC | GTATTTTGCAAAGCGGCGCT | GTTACTGGAAGAAGACGCCC | C(T>C)AGGTGGCCGAGATCTCTG |
|  | 16 | GCTACTGGAAGAAGACGCCC | CTATTCCGCAAAGCGGCGCT | G(T>C)(T>C)ACTGGAAGAAGACGCCC | C(T=C)AGGTGGCCGAGATCTCTG |
|  | 17 | G(C>T)(T>C)ACTGGAAGAAGACGCCC | CTATGCCGCAAAGCGGCGCT | G(T>C)TACTGGAAGAAGACGCCC | C(T>C)AGGTGGCCGAGATCTCTG |
|  | 18 | G(T>C)(T>C)ACTGGAAGAAGACGCCC | CTATTTCGCAAAGCGGCGCT | G(C>T)(T>C)ACTGGAAGAAGACGCCC | C(C>T)AGGTGGCCGAGATCTCTG |
|  | 19 | GCTACTGGAAGAAGACGCCC | CTATTTCGCAAAGCGGCGCT | GTTACTGGAAGAAGACGCCC | CTAGGTGGCCGAGATCTCTG |
|  | 20 | GC(T>C)ACTGGAAGAAGACGCCC | CTATT(C>T)CGCAAAGCGGCGCT | GTTACTGGAAGAAGACGCCC | CTAGGTGGCCGAGATCTCTG |
| 3 | 21 | G(C>T)TACTGGAAGAAGACGCCC | (T>C)TATT(T>C)(C>T)GCAAAGCGGCGCT | GCCACTGGAAGAAGACGCCC | C(T>C)AGGTGGCCGAGATCTCTG |
|  | 22 | GCTACTGGAAGAAGACGCCC | CTATGCCGCAAAGCGGCGCT | GTTA(T>C)TGGAAGAAGACGCCC | C(T>C)AGGTGGCCGAGATCTCTG |
|  | 23 | G(C>T)(T>C)ACTGGAAGAAGACGCCC | (C>T)(G>T)AT(G=T)CCGCAAAGCGGCGCT | GC(C>T)ACTGGAAGAAGACGCCC | CCAGGTGGCCGAGATCTCTG |
|  | 24 | G(G>C)TA(C>T)TGGAAGAAGACGCCC | CTAT(T>G)(C>T)CGCAAAGCGGCGCT | G(T>C)TACTGGAAGAAGACGCCC | (C>T)(A>C>T)AGGTGGCCGAGATCTCTG |
|  | 25 | G(C>T)(T>C)ACTGGAAGAAGACGCCC | TTATT(T>C)(C=T)GCAAAGCGGCGCT | G(T=C)(T>C>G)ACTGGAAGAAGACGCCC | C(T>C)AGGTGGCCGAGATCTCTG |
|  | 26 | GCTACTGGAAGAAGACGCCC | TTATT(C>T)CGCAAAGCGGCGCT | G(T=C)TACTGGAAGAAGACGCCC | CTAGGTGGCCGAGATCTCTG |
|  | 27 | GCTACTGGAAGAAGACGCCC | CTATGCCGCAAAGCGGCGCT | G(T>C)TACTGGAAGAAGACGCCC | C(T=C)AGGTGGCCGAGATCTCTG |
|  | 28 | GC(T>C)ACTGGAAGAAGACGCCC | CTATG(C>T)CGCAAAGCGGCGCT | G(C>T)TACTGGAAGAAGACGCCC | C(T=C)AGGTGGCCGAGATCTCTG |
|  | 29 | G(T>C)(T>C)A(T>C)TGGAAGAAGACGCCC | CTATTCCGCAAAGCGGCGCT | G(C>T)(T>G)ACTGGAAGAAGACGCCC | C(C>T)AGGTGGCCGAGATCTCTG |
|  | 30 | G(C>T)(C>T)ACTGGAAGAAGACGCCC | TTATT(C>T)CGCAAAGCGGCGCT | GC(C>T)ACTGGAAGAAGACGCCC | C(C>T)AGGTGGCCGAGATCTCTG |

Notes:

For each target editing, we conducted at least two plates.

From each plate, we selected 10 colonies for Sanger sequencing (without considering the colony’s colour).

The C-T, C-G, and C-A mutation were highlighted by red, yellow, and green, respectively.

For each target, the green shadow highlighted colonies represented the knockout (KO) mutants.

Table S5 CBEs enabled single and double target editing in *Rhodobacter sphaeroides* KD131

| dCBE series | | nCBE series | |
| --- | --- | --- | --- |
| appA1  CCAGGGCGTCTTCTTCCAGTGGC  CCAGGGCGTCTTCTTCCAGTAAC (2/3)  CCAGGGCGTCTTCTTCCAGTGGC (1/3) | | appA1  CCAGGGCGTCTTCTTCCAGTGGC  CCAGGGCGTCTTCTTCCAGTAAC (3/5)  CCAGGGCGTCTTCTTCCAGTAGC (2/5) | |
| appA2  CCAAGCGCCGCTTTGCGGGATGG  CCAAGCGCCGCTTTGCGGAATAG (2/3)  CCAAGCGCCGCTTTGCGGGATGG (1/3) | | appA2  CCAAGCGCCGCTTTGCGGGATGG  CCAAGCGCCGCTTTGCGAAATAG (1/2)  CCAAGCGCCGCTTTACGGAATAA (1/2) | |
| ppsR1  CGACTCACCACCGACTTCGCGGG  CGATTCACCACCGACTTCGCGGG (6/9)  CGATTTACCACCGACTTCGCGGG (1/9)  TGATTTACCACCGACTTCGCGGG (1/9)  CGACTCACCACCGACTTCGCGGG (1/9) | | ppsR1  CGACTCACCACCGACTTCGCGGG  CGATTCACCACCGACTTCGCGGG (14/27)  CGATTTACCACCGACTTCGCGGG (6/27)  CGATTCACCACTGACTTCGCGGG (2/27)  TGATTCACCACCGACTTCGCGGG (1/27)  TGATTTACCACCGACTTCGCGGG (1/27)  TGACTCACTACCGACTTCGCGGG (1/27)  CGATTTACTACCGACTTCGCGGG (1/27)  CGACTCACCACCGACTTCGCGGG (1/27) | |
| ppsR2  CCGCAGAGATCTCGGCCACCTGG  CCGCAGAGATCTCGGCCACCTGG (3/5)  CCGCAGAGATCTCGGCCACCTAG (1/5)  CCGCAGAGATCTCGGCCACCTAA (1/5) | | ppsR2  CCGCAGAGATCTCGGCCACCTGG  CCGCAGAGATCTCGGCCACCTAG (5/5) | |
| appA1-appA2 | | appA1-appA2 | |
| appA1  CCAGGGCGTCTTCTTCCAGTGGC | appA2  CCAAGCGCCGCTTTGCGGGATGG | appA1  CCAGGGCGTCTTCTTCCAGTGGC | appA2  CCAAGCGCCGCTTTGCGGGATGG |
| CCAGGGCGTCTTCTTCCAGTAAC | CCAAGCGCCGCTTTGCGGGATAG | CCAGGGCGTCTTCTTCCAGTAGC | CCAAGCGCCGCTTTGCGGGATAA |
| CCAGGGCGTCTTCTTCCAGT(A>G)GC | CCAAGCGCCGCTTTGCGGGATAG | CCAGGGCGTCTTCTTCCAGTAGC | CCAAGCGCCGCTTTGCGGGATAG |
| CCAGGGCGTCTTCTTCCAGT(A>G)GC | CCAAGCGCCGCTTTGCGGGA(A>G)G | CCAGGGCGTCTTCTTCCAGTAGC | CCAAGCGCCGCTTTGCGGGATGA |
| CCAGGGCGTCTTCTTCCAGT(A<G)GC | CCAAGCGCCGCTTTGCGGGA(A>G)G | CCAGGGCGTCTTCTTCCAGT(A<G)GC | CCAAGCGCCGCTTTGCGGGATAA |
| CCAGGGCGTCTTCTTCCAGT(A<G)GC | CCAAGCGCCGCTTTGCGGGA(A=G)G | CCAGGGCGTCTTCTTCCAGT(A=G)GC | CCAAGCGCCGCTTTGCGGGA(A>G)G |
| CCAGGGCGTCTTCTTCCAGT(A<G)GC | CCAAGCGCCGCTTTGCGGGA(A=G)G | CCAGGGCGTCTTCTTCCAGT(A<G)GC | CCAAGCGCCGCTTTGCGGGA(A>G)G |
| appA1-ppsR2 | | appA1-ppsR2 | |
| appA1  CCAGGGCGTCTTCTTCCAGTGGC | ppsR2  CCGCAGAGATCTCGGCCACCTGG | appA1  CCAGGGCGTCTTCTTCCAGTGGC | ppsR2  CCGCAGAGATCTCGGCCACCTGG |
| CCAGGGCGTCTTCTTCCAGTAGC | CCGCAGAGATCTCGGCCACCT(A=G)G | CCAGGGCGTCTTCTTCCAGTAAC | CCGCAGAGATCTCGGCCACCTAG |
| CCAGGGCGTCTTCTTCCAGT(A>G)GC | CCGCAGAGATCTCGGCCACCTGG | CCAGGGCGTCTTCTTCCAGTAAC | CCGCAGAGATCTCGGCCACCT(A=G)G |
| CCAGGGCGTCTTCTTCCAGT(A=G)GC | CCGCAGAGATCTCGGCCACCTGG | CCAGGGCGTCTTCTTCCAGTAGC | CCGCAGAGATCTCGGCCACCTGG |
| CCAGGGCGTCTTCTTCCAGT(A=G)GC | CCGCAGAGATCTCGGCCACCTGG | CCAGGGCGTCTTCTTCCAGTAGC | CCGCAGAGATCTCGGCCACCTGG |
| CCAGGGCGTCTTCTTCCAGT(A<G)GC | CCGCAGAGATCTCGGCCACCTGG | CCAGGGCGTCTTCTTCCAGTAGC | CCGCAGAGATCTCGGCCACCTGG |
| CCAGGGCGTCTTCTTCCAGT(A<G)GC | CCGCAGAGATCTCGGCCACCTGG | CCAGGGCGTCTTCTTCCAGT(A>G)GC | CCGCAGAGATCTCGGCCACCTGG |

Notes:

For *Rhodobacter sphaeroides* KD131, we simply tested single and double target editing.

The red colour highlighted colonies meant the mutants.

Table S6 nCBE mediated triple and quadruple target editing in *Rhodobacter sphaeroides* 2.4.1

| The sequence data of nCBE series multiplex target (After the first induction) | | | | | |
| --- | --- | --- | --- | --- | --- |
| Plate replicate | Colony number | appA1-appA2-appA3 | | |  |
|  |  | appA1  GCCACTGGAAGAAGACGCCCTGG | appA2  CCATCCCGCAAAGCGGCGCTTGG | appA3  GCAGGTGACGCTGGCCTATTCGG |  |
| 1 | 1 | GCCACTGGAAGAAGACGCCC | CCATCCCGCAAAGCGGCGCT | GTAGGTGACGCTGGCCTATT |  |
|  | 2 | GCCACTGGAAGAAGACGCCC | CCATCCCGCAAAGCGGCGCT | GTAGGTGACGCTGGCCTATT |  |
|  | 3 | GC(C>T)ACTGGAAGAAGACGCCC | CTATTCCGCAAAGCGGCGCT | GTAGGTGACGCTGGCCTATT |  |
|  | 4 | GCCACTGGAAGAAGACGCCC | CCATCCCGCAAAGCGGCGCT | GTAGGTGACGCTGGCCTATT |  |
|  | 5 | GCCACTGGAAGAAGACGCCC | C(C>T)AT(T=C)CCGCAAAGCGGCGCT | GTAGGTGACGCTGGCCTATT |  |
|  | 6 | GCCACTGGAAGAAGACGCCC | CCATCCCGCAAAGCGGCGCT | GTAGGTGACGCTGGCCTATT |  |
|  | 7 | GCCACTGGAAGAAGACGCCC | CCATCCCGCAAAGCGGCGCT | GTAGGTGACGCTGGCCTATT |  |
|  | 8 | GCCACTGGAAGAAGACGCCC | CCATCCCGCAAAGCGGCGCT | GTAGGTGACGCTGGCCTATT |  |
|  | 9 | GCCACTGGAAGAAGACGCCC | CCATCCCGCAAAGCGGCGCT | GTAGGTGACGCTGGCCTATT |  |
|  | 10 | GCCACTGGAAGAAGACGCCC | CCATCCCGCAAAGCGGCGCT | GTAGGTGACGCTGGCCTATT |  |
| 2 | 11 | G(C>T)(C>T)ACTGGAAGAAGACGCCC | CCATCCCGCAAAGCGGCGCT | GTAGGTGACGCTGGCCTATT |  |
|  | 12 | GC(T>C)ACTGGAAGAAGACGCCC | CCATCCCGCAAAGCGGCGCT | GTAGGTGACGCTGGCCTATT |  |
|  | 13 | GCCACTGGAAGAAGACGCCC | CCATCCCGCAAAGCGGCGCT | GTAGGTGACGCTGGCCTATT |  |
|  | 14 | GCCACTGGAAGAAGACGCCC | CCATCCCGCAAAGCGGCGCT | GTAGGTGACGCTGGCCTATT |  |
|  | 15 | GCCACTGGAAGAAGACGCCC | C(C>T)AT(T=C)CCGCAAAGCGGCGCT | GTAGGTGACGCTGGCCTATT |  |
|  | 16 | GCCACTGGAAGAAGACGCCC | CCATCCCGCAAAGCGGCGCT | GTAGGTGACGCTGGCCTATT |  |
|  | 17 | GCCACTGGAAGAAGACGCCC | CCATCCCGCAAAGCGGCGCT | GTAGGTGACGCTGGCCTATT |  |
|  | 18 | GCCACTGGAAGAAGACGCCC | CCATCCCGCAAAGCGGCGCT | GTAGGTGACGCTGGCCTATT |  |
|  | 19 | GCCACTGGAAGAAGACGCCC | CCATCCCGCAAAGCGGCGCT | GTAGGTGACGCTGGCCTATT |  |
|  | 20 | GCCACTGGAAGAAGACGCCC | CCATCCCGCAAAGCGGCGCT | GTAGGTGACGCTGGCCTATT |  |
| 3 | 21 | GC(C>T)ACTGGAAGAAGACGCCC | CCATCCCGCAAAGCGGCGCT | GTAGGTGACGCTGGCCTATT |  |
|  | 22 | GCCACTGGAAGAAGACGCCC | CCATCCCGCAAAGCGGCGCT | GTAGGTGACGCTGGCCTATT |  |
|  | 23 | GCCACTGGAAGAAGACGCCC | C(C>T)ATCCCGCAAAGCGGCGCT | GTAGGTGACGCTGGCCTATT |  |
|  | 24 | GC(C>T)ACTGGAAGAAGACGCCC | C(C>T)AT(C>T)CCGCAAAGCGGCGCT | GTAGGTGACGCTGGCCTATT |  |
|  | 25 | GCCACTGGAAGAAGACGCCC | CCATCCCGCAAAGCGGCGCT | GTAGGTGACGCTGGCCTATT |  |
|  | 26 | GCCACTGGAAGAAGACGCCC | C(C>T)ATCCCGCAAAGCGGCGCT | GTAGGTGACGCTGGCCTATT |  |
|  | 27 | GCCACTGGAAGAAGACGCCC | CCATCCCGCAAAGCGGCGCT | GTAGGTGACGCTGGCCTATT |  |
|  | 28 | GCCACTGGAAGAAGACGCCC | CCATCCCGCAAAGCGGCGCT | GTAGGTGACGCTGGCCTATT |  |
|  | 29 | GCCACTGGAAGAAGACGCCC | CCATCCCGCAAAGCGGCGCT | GTAGGTGACGCTGGCCTATT |  |
|  | 30 | GCCACTGGAAGAAGACGCCC | CCATCCCGCAAAGCGGCGCT | GTAGGTGACGCTGGCCTATT |  |
| Plate replicate | Colony number | appA3-ppsR1-crtB1 | | |  |
|  |  | appA3  GCAGGTGACGCTGGCCTATTCGG | ppsR1  CGACTCACCACCGACTTCGCGGG | crtB1  ACAGCGCGAGGAGGATGCCGAGG |  |
| 1 | 1 | GCAGGTGACGCTGGCCTATT | CGACTCACCACCGACTTCGC | ACAGCGCGAGGAGGATGCCG |  |
|  | 2 | GCAGGTGACGCTGGCCTATT | CGATTCACCACCGACTTCGC | ATAGTGCGAGGAGGATGCCG |  |
|  | 3 | GCAGGTGACGCTGGCCTATT | CGACTCACCACCGACTTCGC | ACAGCGCGAGGAGGATGCCG |  |
|  | 4 | GCAGGTGACGCTGGCCTATT | CGACTCACCACCGACTTCGC | ACAGCGCGAGGAGGATGCCG |  |
|  | 5 | GCAGGTGACGCTGGCCTATT | CGACTCACCACCGACTTCGC | ACAGCGCGAGGAGGATGCCG |  |
|  | 6 | GCAGGTGACGCTGGCCTATT | CGACTCACCACCGACTTCGC | ATAGCGCGAGGAGGATGCCG |  |
|  | 7 | GCAGGTGACGCTGGCCTATT | CGATTCACCACCGACTTCGC | ACAGCGCGAGGAGGATGCCG |  |
|  | 8 | GCAGGTGACGCTGGCCTATT | CGACTCACCACCGACTTCGC | ATAGCGCGAGGAGGATGCCG |  |
|  | 9 | GCAGGTGACGCTGGCCTATT | CGACTCACCACCGACTTCGC | A(C>T)AGCGCGAGGAGGATGCCG |  |
|  | 10 | GCAGGTGACGCTGGCCTATT | CGACTCACCACCGACTTCGC | A(T>C)AG(T>C)GCGAGGAGGATGCCG |  |
| 2 | 11 | GCAGGTGACGCTGGCCTATT | CGACTCACCACCGACTTCGC | A(T>C)AGCGCGAGGAGGATGCCG |  |
|  | 12 | GCAGGTGACGCTGGCCTATT | CGACTCACCACCGACTTCGC | ATAGCGCGAGGAGGATGCCG |  |
|  | 13 | GCAGGTGACGCTGGCCTATT | CGACTCACCACCGACTTCGC | A(C>T)AG(C=T)GCGAGGAGGATGCCG |  |
|  | 14 | GCAGGTGACGCTGGCCTATT | CGACTCACCACCGACTTCGC | ACAGTGCGAGGAGGATGCCG |  |
|  | 15 | GTAGGTGACGCTGGCCTATT | CGACTCACCACCGACTTCGC | ACAGCGCGAGGAGGATGCCG |  |
|  | 16 | GCAGGTGACGCTGGCCTATT | CGACTCACCACCGACTTCGC | ACAGCGCGAGGAGGATGCCG |  |
|  | 17 | GCAGGTGACGCTGGCCTATT | CGACTCACCACCGACTTCGC | ACAGCGCGAGGAGGATGCCG |  |
|  | 18 | GCAGGTGACGCTGGCCTATT | CGA(T>C)TCACCACCGACTTCGC | ACAGCGCGAGGAGGATGCCG |  |
|  | 19 | GCAGGTGACGCTGGCCTATT | CGACTCACCACCGACTTCGC | ATAGCGCGAGGAGGATGCCG |  |
|  | 20 | GCAGGTGACGCTGGCCTATT | CGACTCACCACCGACTTCGC | ACAGCGCGAGGAGGATGCCG |  |
| 3 | 21 | GTAGGTGACGCTGGCCTATT | CGACTCACCACCGACTTCGC | ACAGCGCGAGGAGGATGCCG |  |
|  | 22 | GCAGGTGACGCTGGCCTATT | CGACTCACCACCGACTTCGC | ACAGCGCGAGGAGGATGCCG |  |
|  | 23 | GCAGGTGACGCTGGCCTATT | CGACTCACCACCGACTTCGC | ACAGCGCGAGGAGGATGCCG |  |
|  | 24 | GCAGGTGACGCTGGCCTATT | CGACTCACCACCGACTTCGC | A(C>T)AGCGCGAGGAGGATGCCG |  |
|  | 25 | GCAGGTGACGCTGGCCTATT | CGACTCACCACCGACTTCGC | ACAGCGCGAGGAGGATGCCG |  |
|  | 26 | GCAGGTGACGCTGGCCTATT | CGACTCACCACCGACTTCGC | ACAGCGCGAGGAGGATGCCG |  |
|  | 27 | GCAGGTGACGCTGGCCTATT | CGACTCACCACCGACTTCGC | ACAGCGCGAGGAGGATGCCG |  |
|  | 28 | GCAGGTGACGCTGGCCTATT | CGACTCACCACCGACTTCGC | ACAGCGCGAGGAGGATGCCG |  |
|  | 29 | GCAGGTGACGCTGGCCTATT | CGACTCACCACCGACTTCGC | A(C>T)AGCGCGAGGAGGATGCCG |  |
|  | 30 | GCAGGTGACGCTGGCCTATT | CGATTCACCACCGACTTCGC | ACAGCGCGAGGAGGATGCCG |  |
| Plate replicate | Colony number | appA3-ppsR1-crtB1-bchG1 | | | |
|  |  | appA3  GCAGGTGACGCTGGCCTATTCGG | ppsR1  CGACTCACCACCGACTTCGCGGG | crtB1  ACAGCGCGAGGAGGATGCCGAGG | bchG1  TGAGCCAGGCCGCGAACAACTGG |
| 1 | 1 | GCAGGTGACGCTGGCCTATT | CGATTCACCACCGACTTCGC | A(C>T)AGCGCGAGGAGGATGCCG | TGAGCCAGGCCGCGAACAAC |
|  | 2 | GCAGGTGACGCTGGCCTATT | CGATTTACCACCGACTTCGC | ACAGCGCGAGGAGGATGCCG | TGAGACAGGCCGCGAACAAC |
|  | 3 | G(C>T)AGGTGACGCTGGCCTATT | CGATTCACCACCGACTTCGC | ACAGCGCGAGGAGGATGCCG | TGAGCCAGGCCGCGAACAAC |
|  | 4 | GTAGGTGACGCTGGCCTATT | CGACTCACCACCGACTTCGC | ACAGCGCGAGGAGGATGCCG | TGAG(C>T)CAGGCCGCGAACAAC |
|  | 5 | G(C>T)AGGTGACGCTGGCCTATT | CGA(T>C)TCACCACCGACTTCGC | ACAGCGCGAGGAGGATGCCG | TGAGCCAGGCCGCGAACAAC |
|  | 6 | GTAGGTGACGCTGGCCTATT | CGACTCACCACCGACTTCGC | ACAGCGCGAGGAGGATGCCG | TGAGCCAGGCCGCGAACAAC |
|  | 7 | G(T=C)AGGTGACGCTGGCCTATT | CGATTCACCACCGACTTCGC | ACAGCGCGAGGAGGATGCCG | TGAGCCAGGCCGCGAACAAC |
|  | 8 | GCAGGTGACGCTGGCCTATT | CGA(C>T)TCACCACCGACTTCGC | ACAGCGCGAGGAGGATGCCG | TGAG(C=T)CAGGCCGCGAACAAC |
|  | 9 | GTAGGTGACGCTGGCCTATT | CGACTCACCACCGACTTCGC | ACAGCGCGAGGAGGATGCCG | TGAGCCAGGCCGCGAACAAC |
|  | 10 | G(T>C)AGGTGACGCTGGCCTATT | CGATTCACCACCGACTTCGC | ACAG(C>T)GCGAGGAGGATGCCG | TGAGCCAGGCCGCGAACAAC |
| 2 | 11 | GCAGGTGACGCTGGCCTATT | CGATTCACCACCGACTTCGC | ACAGCGCGAGGAGGATGCCG | TGAGCCAGGCCGCGAACAAC |
|  | 12 | GCAGGTGACGCTGGCCTATT | CGAATCACCACCGACTTCGC | ACAGCGCGAGGAGGATGCCG | TGAGCCAGGCCGCGAACAAC |
|  | 13 | G(C>T)AGGTGACGCTGGCCTATT | CGATTCACCACCGACTTCGC | ACAGCGCGAGGAGGATGCCG | TGAG(C>T)CAGGCCGCGAACAAC |
|  | 14 | GCAGGTGACGCTGGCCTATT | CGA(G>T)TCACCACCGACTTCGC | ACAGCGCGAGGAGGATGCCG | TGAG(T=C)CAGGCCGCGAACAAC |
|  | 15 | GTAGGTGACGCTGGCCTATT | CGACTCACCACCGACTTCGC | ACAGCGCGAGGAGGATGCCG | TGAGCCAGGCCGCGAACAAC |
|  | 16 | G(T=C)AGGTGACGCTGGCCTATT | CGATTCACCACCGACTTCGC | ACAGCGCGAGGAGGATGCCG | TGAGCCAGGCCGCGAACAAC |
|  | 17 | G(T>C)AGGTGACGCTGGCCTATT | CGATTCACCACCGACTTCGC | ACAGCGCGAGGAGGATGCCG | TGAGCCAGGCCGCGAACAAC |
|  | 18 | G(T>C)AGGTGACGCTGGCCTATT | CGATTCACCACCGACTTCGC | ACAGCGCGAGGAGGATGCCG | TGAGCCAGGCCGCGAACAAC |
|  | 19 | GCAGGTGACGCTGGCCTATT | CGATTCACCACCGACTTCGC | ACAGCGCGAGGAGGATGCCG | TGAGCCAGGCCGCGAACAAC |
|  | 20 | GTAGGTGACGCTGGCCTATT | CGATTTACCACCGACTTCGC | ACAGCGCGAGGAGGATGCCG | TGAGCCAGGCCGCGAACAAC |
| 3 | 21 | GTAGGTGACGCTGGCCTATT | CGA(T=C)TCACCACCGACTTCGC | ACAGCGCGAGGAGGATGCCG | TGAGCCAGGCCGCGAACAAC |
|  | 22 | GCAGGTGACGCTGGCCTATT | CGA(G=C)TCACCACCGACTTCGC | ACAGCGCGAGGAGGATGCCG | TGAGCCAGGCCGCGAACAAC |
|  | 23 | GCAGGTGACGCTGGCCTATT | CGA(T>C)TCACCACCGACTTCGC | ATAGCGCGAGGAGGATGCCG | TGAGCCAGGCCGCGAACAAC |
|  | 24 | GTAGGTGACGCTGGCCTATT | CGACTCACCACCGACTTCGC | ACAGCGCGAGGAGGATGCCG | TGAGCCAGGCCGCGAACAAC |
|  | 25 | GCAGGTGACGCTGGCCTATT | CGACTCACCACCGACTTCGC | A(C>T)AGCGCGAGGAGGATGCCG | TGAGCCAGGCCGCGAACAAC |
|  | 26 | GCAGGTGACGCTGGCCTATT | CGATTCACCACCGACTTCGC | ACAGCGCGAGGAGGATGCCG | TGAGCCAGGCCGCGAACAAC |
|  | 27 | GTAGGTGACGCTGGCCTATT | TGATTCACCA(C>T)CGACTTCGC | ACAGCGCGAGGAGGATGCCG | TGAGCCAGGCCGCGAACAAC |
|  | 28 | GCAGGTGACGCTGGCCTATT | CGACTCACCACCGACTTCGC | ACAGCGCGAGGAGGATGCCG | TGAGCCAGGCCGCGAACAAC |
|  | 29 | GCAGGTGACGCTGGCCTATT | CGATTCACCACCGACTTCGC | ACAGCGCGAGGAGGATGCCG | TGAGCCAGGCCGCGAACAAC |
|  | 30 | GCAGGTGACGCTGGCCTATT | CGATTCACCACCGACTTCGC | ACAGCGCGAGGAGGATGCCG | TGAGCCAGGCCGCGAACAAC |
| The sequence data of nCBE series multiplex target (After the second induction) | | | | | |
| Streak from the first induction clone (grey shadow highlight) | Screening  colony  number | appA1-appA2-appA3 | | |  |
|  |  | appA1  GCCACTGGAAGAAGACGCCCTGG | appA2  CCATCCCGCAAAGCGGCGCTTGG | appA3  GCAGGTGACGCTGGCCTATTCGG |  |
| Plate 1  NO.3 clone | 1 | GCTACTGGAAGAAGACGCCC | CTATTCCGCAAAGCGGCGCT | GTAGGTGACGCTGGCCTATT |  |
|  | 2 | G(C>T)(T>C)ACTGGAAGAAGACGCCC | CTATTCCGCAAAGCGGCGCT | GTAGGTGACGCTGGCCTATT |  |
|  | 3 | GCTACTGGAAGAAGACGCCC | CTATTCCGCAAAGCGGCGCT | GTAGGTGACGCTGGCCTATT |  |
|  | 4 | GCTACTGGAAGAAGACGCCC | CTATTCCGCAAAGCGGCGCT | GTAGGTGACGCTGGCCTATT |  |
|  | 5 | GCTACTGGAAGAAGACGCCC | CTATTCCGCAAAGCGGCGCT | GTAGGTGACGCTGGCCTATT |  |
| Plate 2  NO.12 clone | 1 | GCTACTGGAAGAAGACGCCC | TTATGCCGCAAAGCGGCGCT | GTAGGTGACGCTGGCCTATT |  |
|  | 2 | GCTACTGGAAGAAGACGCCC | CTAT(C>T)CCGCAAAGCGGCGCT | GTAGGTGACGCTGGCCTATT |  |
|  | 3 | GCTACTGGAAGAAGACGCCC | (C>T)(T>C)AT(T>C)CCGCAAAGCGGCGCT | GTAGGTGACGCTGGCCTATT |  |
|  | 4 | G(C>G)TACTGGAAGAAGACGCCC | C(T>C)AT(C=G)CCGCAAAGCGGCGCT | GTAGGTGACGCTGGCCTATT |  |
|  | 5 | GCTACTGGAAGAAGACGCCC | CTATGCCGCAAAGCGGCGCT | GTAGGTGACGCTGGCCTATT |  |
| Plate 3  NO.21 clone | 1 | GCGACTGGAAGAAGACGCCC | CGATCCCGCAAAGCGGCGCT | GTAGGTGACGCTGGCCTATT |  |
|  | 2 | GCCACTGGAAGAAGACGCCC | CCATCCCGCAAAGCGGCGCT | GTAGGTGACGCTGGCCTATT |  |
|  | 3 | GCCACTGGAAGAAGACGCCC | CCATCCCGCAAAGCGGCGCT | GTAGGTGACGCTGGCCTATT |  |
|  | 4 | GCCACTGGAAGAAGACGCCC | CCATCCCGCAAAGCGGCGCT | GTAGGTGACGCTGGCCTATT |  |
|  | 5 | GC(T>C)ACTGGAAGAAGACGCCC | C(C>T)ATCCCGCAAAGCGGCGCT | GTAGGTGACGCTGGCCTATT |  |
| Streak from the first induction clone (grey shadow highlight) | Screening  colony  number | appA3-ppsR1-crtB1 | | |  |
|  |  | appA3  GCAGGTGACGCTGGCCTATTCGG | ppsR1  CGACTCACCACCGACTTCGCGGG | crtB1  ACAGCGCGAGGAGGATGCCGAGG |  |
| Plate 1  NO.2 clone | 1 | GCAGGTGACGCTGGCCTATT | CGATTCACCACCGACTTCGC | ATAGCGCGAGGAGGATGCCG |  |
|  | 2 | GCAGGTGACGCTGGCCTATT | CGATTCACCACCGACTTCGC | ATAGCGCGAGGAGGATGCCG |  |
|  | 3 | GCAGGTGACGCTGGCCTATT | CGATTCACCACCGACTTCGC | ATAGCGCGAGGAGGATGCCG |  |
|  | 4 | GCAGGTGACGCTGGCCTATT | CGATTCACCACCGACTTCGC | ATAGCGCGAGGAGGATGCCG |  |
|  | 5 | GCAGGTGACGCTGGCCTATT | CGATTCACCACCGACTTCGC | ATAGCGCGAGGAGGATGCCG |  |
| Plate 2  NO.15 clone | 1 | GGAGGTGACGCTGGCCTATT | CGACTCACCACCGACTTCGC | ACAGCGCGAGGAGGATGCCG |  |
|  | 2 | GTAGGTGACGCTGGCCTATT | CGACTCACCACCGACTTCGC | ACAGCGCGAGGAGGATGCCG |  |
|  | 3 | GTAGGTGACGCTGGCCTATT | CGACTCACCACCGACTTCGC | ACAGCGCGAGGAGGATGCCG |  |
|  | 4 | GTAGGTGACGCTGGCCTATT | CGACTCACCACCGACTTCGC | ATAGTGCGAGGAGGATGCCG |  |
|  | 5 | GTAGGTGACGCTGGCCTATT | CGACTCACCACCGACTTCGC | ATAGTGGGAGGAGGATGCCG |  |
| Plate 3  NO.30 clone | 1 | GCAGGTGACGCTGGCCTATT | CGATTCACCACCGACTTCGC | ATAGTGCGAGGAGGATGCCG |  |
|  | 2 | GCAGGTGACGCTGGCCTATT | CGATTCACCACCGACTTCGC | ATAGTGCGAGGAGGATGCCG |  |
|  | 3 | GTAGGTGACGCTGGCCTATT | CGATTCACCACCGACTTCGC | ATAGTGCGAGGAGGATGCCG |  |
|  | 4 | GCAGGTGACGCTGGCCTATT | CGATTCACCACCGACTTCGC | ACAGCGCGAGGAGGATGCCG |  |
|  | 5 | GCAGGTGACGCTGGCCTATT | CGATTCACCACCGACTTCGC | ACAGCGCGAGGAGGATGCCG |  |
| Streak from the first induction clone (grey shadow highlight) | Screening  Colony  number | appA3-ppsR1-crtB1-bchG1 | | | |
|  |  | appA3  GCAGGTGACGCTGGCCTATTCGG | ppsR1  CGACTCACCACCGACTTCGCGGG | crtB1  ACAGCGCGAGGAGGATGCCGAGG | bchG1  TGAGCCAGGCCGCGAACAACTGG |
| Plate 1  NO.2 clone | 1 | GTAGGTGACGCTGGCCTATT | CGATTTACCACCGACTTCGC | ACAGCGCGAGGAGGATGCCG | TGAGACAGGCCGCGAACAAC |
|  | 2 | GGAGGTGACGCTGGCCTATT | CGATTTACCACCGACTTCGC | ACAGCGCGAGGAGGATGCCG | TGAGACAGGCCGCGAACAAC |
|  | 3 | GCAGGTGACGCTGGCCTATT | CGATTTACCACCGACTTCGC | ACAGGGCGAGGAGGATGCCG | TGAGACAGGCCGCGAACAAC |
|  | 4 | GCAGGTGACGCTGGCCTATT | CGATTTACCACCGACTTCGC | ACAGCGCGAGGAGGATGCCG | TGAGACAGGCCGCGAACAAC |
|  | 5 | GTAGGTGACGCTGGCCTATT | CGATTTACCACCGACTTCGC | ACAGCGCGAGGAGGATGCCG | TGAGACAGGCCGCGAACAAC |
| Plate 2  NO.14 clone | 1 | GTAGGTGACGCTGGCCTATT | CGAGTCACCACCGACTTCGC | ATAGCGCGAGGAGGATGCCG | TGAGCCAGGCCGCGAACAAC |
|  | 2 | GCAGGTGACGCTGGCCTATT | CGAGTCACCACCGACTTCGC | ACAGCGCGAGGAGGATGCCG | TGAGTCAGGCCGCGAACAAC |
|  | 3 | GTAGGTGACGCTGGCCTATT | CGAGTCACCACCGACTTCGC | ACAGCGCGAGGAGGATGCCG | TGAGTCAGGCCGCGAACAAC |
|  | 4 | GTAGGTGACGCTGGCCTATT | CGAGTCACCACCGACTTCGC | ATAGCGCGAGGAGGATGCCG | TGAGCCAGGCCGCGAACAAC |
|  | 5 | GTAGGTGACGCTGGCCTATT | CGAGTCACCACCGACTTCGC | ATAGCGCGAGGAGGATGCCG | TGAGCCAGGCCGCGAACAAC |
| Plate 3  NO.23 clone | 1 | GCAGGTGACGCTGGCCTATT | CGATTCACCACCGACTTCGC | ACAGCGCGAGGAGGATGCCG | TGAGCCAGGCCGCGAACAAC |
|  | 2 | GCAGGTGACGCTGGCCTATT | CGACTCACCACCGACTTCGC | ATAGCGCGAGGAGGATGCCG | TGAGCCAGGCCGCGAACAAC |
|  | 3 | GCAGGTGACGCTGGCCTATT | CGATTCACCACCGACTTCGC | ACAGCGCGAGGAGGATGCCG | TGAGCCAGGCCGCGAACAAC |
|  | 4 | GCAGGTGACGCTGGCCTATT | CGACTCACCACCGACTTCGC | ATAGCGCGAGGAGGATGCCG | TGAGCCAGGCCGCGAACAAC |
|  | 5 | GCAGGTGACGCTGGCCTATT | CGACTCACCACCGACTTCGC | ATAGCGCGAGGAGGATGCCG | TGAGCCAGGCCGCGAACAAC |

Notes:

The C-T, C-G, and C-A mutation were highlighted by red, yellow, and green, respectively.

The grey shadow highlighted colonies represented the colonies choosing from the first induction plate, which were then streaked onto the new plate for the second induction.

The green shadow highlighted colonies meant the knockout (KO) mutants.

Table S7 ABEs mediated single target editing in *Rhodobacter sphaeroides* 2.4.1

| The sequence data of dABE series single target (After the first induction) | | | | | |
| --- | --- | --- | --- | --- | --- |
| Plate replicate | Colony number | appA0  AAGGATGCAACACGACCTCGAGG | ppsR0  GAAGAGACATGCTGGCCGGCGGG | crtB0  TCATTCCGCGGCAAGCCTTTCAGCAGG | bchG0  GGGCACGGAGCGGGGGTGTAAGG |
| Total | 1 | AAGGATGCAACACGACCTCGAGG | GAAGAGACATGCTGGCCGGCGGG | TCATTCCGCGGCAAGCCTTTCAGCAGG | GGGCACGGAGCGGGGGTGTAAGG |
|  | 2 | AAGGATGCAACACGACCTCGAGG | GAAGAGACATGCTGGCCGGCGGG | TCATTCCGCGGCAAGCCTTTCAGCAGG | GGGCACGGAGCGGGGGTGTAAGG |
|  | 3 | AAGGATGCAACACGACCTCGAGG | GAAGAGACATGCTGGCCGGCGGG | TCATTCCGCGGCAAGCCTTTCAGCAGG | GGGCACGGAGCGGGGGTGTAAGG |
|  | 4 | AAGGATGCAACACGACCTCGAGG | GAAGAGACATGCTGGCCGGCGGG | TCATTCCGCGGCAAGCCTTTCAGCAGG | GGGCACGGAGCGGGGGTGTAAGG |
|  | 5 | AAGGATGCAACACGACCTCGAGG | GAAGAGACATGCTGGCCGGCGGG | TCATTCCGCGGCAAGCCTTTCAGCAGG | GGGCACGGAGCGGGGGTGTAAGG |
|  | 6 | AAGGATGCAACACGACCTCGAGG | GAAGAGACATGCTGGCCGGCGGG | TCATTCCGCGGCAAGCCTTTCAGCAGG | GGGCACGGAGCGGGGGTGTAAGG |
|  | 7 | AAGGATGCAACACGACCTCGAGG | GAAGAGACATGCTGGCCGGCGGG | TCATTCCGCGGCAAGCCTTTCAGCAGG | GGGCACGGAGCGGGGGTGTAAGG |
|  | 8 | AAGGATGCAACACGACCTCGAGG | GAAGAGACATGCTGGCCGGCGGG | TCATTCCGCGGCAAGCCTTTCAGCAGG | GGGCACGGAGCGGGGGTGTAAGG |
| The sequence data of dABE series single target (After the second induction) | | | | | |
| Plate replicate | Colony number | appA0  AAGGATGCAACACGACCTCGAGG | ppsR0  GAAGAGACATGCTGGCCGGCGGG | crtB0  TCATTCCGCGGCAAGCCTTTCAGCAGG | bchG0  GGGCACGGAGCGGGGGTGTAAGG |
| 1 | 1 | AAGGATGCAACACGACCTCGAGG | GAAGAGACATGCTGGCCGGCGGG | TCATTCCGCGGCAAGCCTTTCAGCAGG | GGGCACGGAGCGGGGGTGTAAGG |
|  | 2 | AAGGATGCAACACGACCTCGAGG | GAAGAGACATGCTGGCCGGCGGG | TCATTCCGCGGCAAGCCTTTCAGCAGG | GGGCACGGAGCGGGGGTGTAAGG |
|  | 3 | AAGGATGCAACACGACCTCGAGG | GAAGAGACATGCTGGCCGGCGGG | TCATTCCGCGGCAAGCCTTTCAGCAGG | GGGCACGGAGCGGGGGTGTAAGG |
|  | 4 | AAGGATGCAACACGACCTCGAGG | GAAGAGACATGCTGGCCGGCGGG | TCATTCCGCGGCAAGCCTTTCAGCAGG | GGGCACGGAGCGGGGGTGTAAGG |
|  | 5 | AAGGATGCAACACGACCTCGAGG | GAAGAGACATGCTGGCCGGCGGG | TCATTCCGCGGCAAGCCTTTCAGCAGG | GGGCACGGAGCGGGGGTGTAAGG |
|  | 6 | AAGGGTGCAACACGACCTCGAGG | GAAGAGACATGCTGGCCGGCGGG | TCATTCCGCGGCAAGCCTTTCAGCAGG | GGGCACGGAGCGGGGGTGTAAGG |
|  | 7 | AAGGATGCAACACGACCTCGAGG | GAAGAGACATGCTGGCCGGCGGG | TCATTCCGCGGCAAGCCTTTCAGCAGG | GGGCACGGAGCGGGGGTGTAAGG |
|  | 8 | AAGGATGCAACACGACCTCGAGG | GAAGAGACATGCTGGCCGGCGGG | TCATTCCGCGGCAAGCCTTTCAGCAGG | GGGCACGGAGCGGGGGTGTAAGG |
|  | 9 | AAGGATGCAACACGACCTCGAGG | GAAGAGACATGCTGGCCGGCGGG | TCATTCCGCGGCAAGCCTTTCAGCAGG | GGGCACGGAGCGGGGGTGTAAGG |
|  | 10 | AAGGATGCAACACGACCTCGAGG | GAAGAGACATGCTGGCCGGCGGG | TCATTCCGCGGCAAGCCTTTCAGCAGG | GGGCACGGAGCGGGGGTGTAAGG |
| 2 | 11 | AAGGATGCAACACGACCTCGAGG | GAAGAGACATGCTGGCCGGCGGG | TCATTCCGCGGCAAGCCTTTCAGCAGG | GGGCACGGAGCGGGGGTGTAAGG |
|  | 12 | AAGGATGCAACACGACCTCGAGG | GAAGAGACATGCTGGCCGGCGGG | TCATTCCGCGGCAAGCCTTTCAGCAGG | GGGCACGGAGCGGGGGTGTAAGG |
|  | 13 | AAGGATGCAACACGACCTCGAGG | GAAGAGACATGCTGGCCGGCGGG | TCATTCCGCGGCAAGCCTTTCAGCAGG | GGGCACGGAGCGGGGGTGTAAGG |
|  | 14 | AAGGATGCAACACGACCTCGAGG | GAAGAGACATGCTGGCCGGCGGG | TCATTCCGCGGCAAGCCTTTCAGCAGG | GGGCACGGAGCGGGGGTGTAAGG |
|  | 15 | AAGGATGCAACACGACCTCGAGG | GAAGAGACATGCTGGCCGGCGGG | TCATTCCGCGGCAAGCCTTTCAGCAGG | GGGCACGGAGCGGGGGTGTAAGG |
|  | 16 | AAGGATGCAACACGACCTCGAGG | GAAGAGACATGCTGGCCGGCGGG | TCATTCCGCGGCAAGCCTTTCAGCAGG | GGGCACGGAGCGGGGGTGTAAGG |
|  | 17 | AAGGATGCAACACGACCTCGAGG | GAAGAGACATGCTGGCCGGCGGG | TCATTCCGCGGCAAGCCTTTCAGCAGG | GGGCACGGAGCGGGGGTGTAAGG |
|  | 18 | AAGGATGCAACACGACCTCGAGG | GAAGAGACATGCTGGCCGGCGGG | TCATTCCGCGGCAAGCCTTTCAGCAGG | GGGCACGGAGCGGGGGTGTAAGG |
|  | 19 | AAGGATGCAACACGACCTCGAGG | GAAGAGACATGCTGGCCGGCGGG | TCATTCCGCGGCAAGCCTTTCAGCAGG | GGGCACGGAGCGGGGGTGTAAGG |
|  | 20 | AAGGATGCAACACGACCTCGAGG | GAAGAGACATGCTGGCCGGCGGG | TCATTCCGCGGCAAGCCTTTCAGCAGG | GGGCACGGAGCGGGGGTGTAAGG |
| The sequence data of nABE series single target (After the first induction) | | | | | |
| Plate replicate | Colony number | appA0  AAGGATGCAACACGACCTCGAGG | ppsR0  GAAGAGACATGCTGGCCGGCGGG | crtB0  TCATTCCGCGGCAAGCCTTTCAGCAGG | bchG0  GGGCACGGAGCGGGGGTGTAAGG |
| Total | 1 | AAGG(A<G)TGCAACACGACCTCGAGG | GAAGAGACATGCTGGCCGGCGGG | TCATTCCGCGGCAAGCCTTTCAGCAGG | GGGCACGGAGCGGGGGTGTAAGG |
|  | 2 | AAGGATGCAACACGACCTCGAGG | GAAGAGACATGCTGGCCGGCGGG | TCATTCCGCGGCAAGCCTTTCAGCAGG | GGGC(A<G)CGGAGCGGGGGTGTAAGG |
|  | 3 | AAGGATGCAACACGACCTCGAGG | GAAGAGACATGCTGGCCGGCGGG | TCATTCCGCGGCAAGCCTTTCAGCAGG | GGGCACGGAGCGGGGGTGTAAGG |
|  | 4 | AAGGATGCAACACGACCTCGAGG | GAAG(A<G)GACATGCTGGCCGGCGGG | TCATTCCGCGGCAAGCCTTTCAGCAGG | GGGCACGGAGCGGGGGTGTAAGG |
|  | 5 | AAGG(A<G)TGCAACACGACCTCGAGG | GAAGAGACATGCTGGCCGGCGGG | TCATTCCGCGGCAAGCCTTTCAGCAGG | GGGCACGGAGCGGGGGTGTAAGG |
|  | 6 | AAGGATGCAACACGACCTCGAGG | GAAGAGACATGCTGGCCGGCGGG | TCATTCCGCGGCAAGCCTTTCAGCAGG | GGGCACGGAGCGGGGGTGTAAGG |
|  | 7 | AAGG(A<G)TGCAACACGACCTCGAGG | GAAGAGACATGCTGGCCGGCGGG | TCATTCCGCGGCAAGCCTTTCAGCAGG | GGGCACGGAGCGGGGGTGTAAGG |
|  | 8 | AAGGATGCAACACGACCTCGAGG | GAAGAGACATGCTGGCCGGCGGG | TCATTCCGCGGCAAGCCTTTCAGCAGG | GGGCACGGAGCGGGGGTGTAAGG |
| The sequence data of nABE series single target (After the second induction) | | | | | |
| Plate replicate | Colony number | appA0  AAGGATGCAACACGACCTCGAGG | ppsR0  GAAGAGACATGCTGGCCGGCGGG | crtB0  TCATTCCGCGGCAAGCCTTTCAGCAGG | bchG0  GGGCACGGAGCGGGGGTGTAAGG |
| 1 | 1 | AAGGGTGCAACACGACCTCGAGG | GAAGAGACATGCTGGCCGGCGGG | TCATTCCGCGGCAAGCCTTTCAGCAGG | GGGCACGGAGCGGGGGTGTAAGG |
|  | 2 | AAGGATGCAACACGACCTCGAGG | GAAGAGACATGCTGGCCGGCGGG | TCATTCCGCGGCAAGCCTTTCAGCAGG | GGGCACGGAGCGGGGGTGTAAGG |
|  | 3 | AAGGATGCAACACGACCTCGAGG | GAAGAGGCATGCTGGCCGGCGGG | TCATTCCGCGGCAAGCCTTTCAGCAGG | GGGCACGGAGCGGGGGTGTAAGG |
|  | 4 | AAGGATGCAACACGACCTCGAGG | GAAGAGACATGCTGGCCGGCGGG | TCATTCCGCGGCAAGCCTTTCAGCAGG | GGGCACGGAGCGGGGGTGTAAGG |
|  | 5 | AAGGATGCAACACGACCTCGAGG | GAAGAGGCATGCTGGCCGGCGGG | TCATTCCGCGGCAAGCCTTTCAGCAGG | GGGCACGGAGCGGGGGTGTAAGG |
|  | 6 | AAGGGTGCAACACGACCTCGAGG | GAAGAGACATGCTGGCCGGCGGG | TCATTCCGCGGCAAGCCTTTCAGCAGG | GGGCACGGAGCGGGGGTGTAAGG |
|  | 7 | AAGGATGCAACACGACCTCGAGG | GAAGGGACATGCTGGCCGGCGGG | TCATTCCGCGGCAAGCCTTTCAGCAGG | GGGCACGGAGCGGGGGTGTAAGG |
|  | 8 | AAGG(A>G)TGCAACACGACCTCGAGG | GAAGAGACATGCTGGCCGGCGGG | TCATTCCGCGGCAAGCCTTTCAGCAGG | GGGCACGGAGCGGGGGTGTAAGG |
|  | 9 | AAGG(A>G)TGCAACACGACCTCGAGG | GAAGAGACATGCTGGCCGGCGGG | TCATTCCGCGGCAAGCCTTTCAGCAGG | GGGCACGGAGCGGGGGTGTAAGG |
|  | 10 | AAGG(A>G)TGCAACACGACCTCGAGG | GAAGGGACATGCTGGCCGGCGGG | TCATTCCGCGGCAAGCCTTTCAGCAGG | GGGC(A=G)CGGAGCGGGGGTGTAAGG |
| 2 | 11 | AAGGGTGCAACACGACCTCGAGG | GAAGAGACATGCTGGCCGGCGGG | TCATTCCGCGGCAAGCCTTTCAGCAGG | GGGC(A>G)CGGAGCGGGGGTGTAAGG |
|  | 12 | AAGGGTGCAACACGACCTCGAGG | GAAGGGGCATGCTGGCCGGCGGG | TCATTCCGCGGCAAGCCTTTCAGCAGG | GGGCACGGAGCGGGGGTGTAAGG |
|  | 13 | AAGGGTGCAACACGACCTCGAGG | GAAGAGACATGCTGGCCGGCGGG | TCATTCCGCGGCAAGCCTTTCAGCAGG | GGGCACGGAGCGGGGGTGTAAGG |
|  | 14 | AAGG(A>G)TGCAACACGACCTCGAGG | GAAGAGACATGCTGGCCGGCGGG | TCATTCCGCGGCAAGCCTTTCAGCAGG | GGGCACGGAGCGGGGGTGTAAGG |
|  | 15 | AAGGGTGCAACACGACCTCGAGG | GAAGAGACATGCTGGCCGGCGGG | TCATTCCGCGGCAAGCCTTTCAGCAGG | GGGCACGGAGCGGGGGTGTAAGG |
|  | 16 | AAGGATGCAACACGACCTCGAGG | GAAG(A>G)GACATGCTGGCCGGCGGG | TCATTCCGCGGCAAGCCTTTCAGCAGG | GGGCACGGAGCGGGGGTGTAAGG |
|  | 17 | AAGG(A>G)TGCAACACGACCTCGAGG | GAAGAGACATGCTGGCCGGCGGG | TCATTCCGCGGCAAGCCTTTCAGCAGG | GGGC(A>G)CGGAGCGGGGGTGTAAGG |
|  | 18 | AAGGATGCAACACGACCTCGAGG | GAAG(A>G)G(A>G)CATGCTGGCCGGCGGG | TCATTCCGCGGCAAGCCTTTCAGCAGG | GGGCACGGAGCGGGGGTGTAAGG |
|  | 19 | AAGGATGCAACACGACCTCGAGG | GAAGAGACATGCTGGCCGGCGGG | TCATTCCGCGGCAAGCCTTTCAGCAGG | GGGCACGGAGCGGGGGTGTAAGG |
|  | 20 | AAGGATGCAACACGACCTCGAGG | GAAGAGACATGCTGGCCGGCGGG | TCATTCCGCGGCAAGCCTTTCAGCAGG | GGGCACGGAGCGGGGGTGTAAGG |
| 3 | 21 |  | GAAG(A>G)G(A>G)CATGCTGGCCGGCGGG |  |  |
|  | 22 |  | GAAGAGACATGCTGGCCGGCGGG |  |  |
|  | 23 |  | GAAGGGGCATGCTGGCCGGCGGG |  |  |
|  | 24 |  | GAAGAGACATGCTGGCCGGCGGG |  |  |
|  | 25 |  | GAAGAGACATGCTGGCCGGCGGG |  |  |
|  | 26 |  | GAAG(A>G)GACATGCTGGCCGGCGGG |  |  |
|  | 27 |  | GAAGAGACATGCTGGCCGGCGGG |  |  |
|  | 28 |  | GAAGAG(G>A)CATGCTGGCCGGCGGG |  |  |
|  | 29 |  | GAAGAGACATGCTGGCCGGCGGG |  |  |
|  | 30 |  | GAAG(G=A)GACATGCTGGCCGGCGGG |  |  |

Notes:

We chose 8 colonies from the first induction plates (total three plates).

We selected two colonies from the first induction plates, and streaked onto the new plate for the second induction.

The A-G mutation were highlighted by yellow.

Table S8 CoQ10 fermentation assay of ubiquinone series KO mutants and control strains

| Sample | Peak area of CoQ10 | Tested CoQ10 concentration (mg/L) | Practical CoQ10 concentration (mg/L) | PH | Packed cell volume (%) | CoQ10 content (mg/L PCV) |
| --- | --- | --- | --- | --- | --- | --- |
| Standard | 4663.01 | 233.15 |  | | | |
| △ubiA | 89.17 | 4.46 | 13.38 | 6.84 | 12% | 1.12 |
|  | 94.11 | 4.71 | 14.12 | 6.89 | 11% | 1.28 |
|  | 80.96 | 4.05 | 12.14 | 6.81 | 12% | 1.01 |
| △ubiB | 557.42 | 27.87 | 83.61 | 6.85 | 12% | 6.97 |
|  | 544.38 | 27.22 | 81.66 | 6.86 | 12% | 6.81 |
|  | 581.72 | 29.09 | 87.26 | 6.70 | 12% | 7.27 |
| △ubiD | 541.89 | 27.09 | 81.28 | 7.07 | 10% | 8.13 |
|  | 534.37 | 26.72 | 80.16 | 7.14 | 10% | 8.02 |
|  | 532.90 | 26.65 | 79.94 | 7.09 | 11% | 7.27 |
| △ubiF | 0.00 | 0.00 | 0.00 | 8.05 | 5% | 0 |
|  | 0.00 | 0.00 | 0.00 | 8.11 | 7% | 0 |
|  | 0.00 | 0.00 | 0.00 | 7.93 | 6% | 0 |
| △ubiG | 248.80 | 12.44 | 37.32 | 6.69 | 8% | 4.67 |
|  | 232.55 | 11.63 | 34.88 | 6.70 | 8% | 4.36 |
|  | 238.25 | 11.91 | 35.74 | 6.68 | 7% | 5.11 |
| △ubiX | 284.99 | 14.25 | 42.75 | 7.72 | 10% | 4.28 |
|  | 326.58 | 16.33 | 48.99 | 7.62 | 11% | 4.45 |
|  | 371.58 | 18.58 | 55.74 | 7.61 | 10% | 5.57 |
| △crtB | 672.02 | 33.60 | 100.80 | 7.33 | 12% | 8.40 |
|  | 661.91 | 33.10 | 99.29 | 7.24 | 11% | 9.03 |
|  | 669.29 | 33.46 | 100.39 | 7.28 | 11% | 9.13 |
| △crtB-  △ppsR | 713.22 | 35.66 | 106.98 | 7.39 | 10% | 10.70 |
|  | 836.46 | 41.82 | 125.47 | 7.06 | 10% | 12.55 |
|  | 762.66 | 38.13 | 114.40 | 7.17 | 10% | 11.44 |
| WT | 553.93 | 27.70 | 83.09 | 7.26 | 10% | 8.31 |
|  | 559.46 | 27.97 | 83.92 | 6.97 | 10% | 8.39 |
|  | 553.47 | 27.67 | 83.02 | 6.98 | 10% | 8.30 |
